# Impact of *Wolbachia-mediated* incompatible and sterile insect techniques on dengue incidence: a large-scale cluster randomised controlled trial

**DOI:** 10.1016/j.ebiom.2026.106476

**Published:** 2026-09-10

**Authors:** Chia-chen Chang, Jue Tao Lim, Chee Seng Chong, Diyar Mailepessov, Borame Dickens, Yichen Zhai, Lu Deng, Caleb Lee, Li Yun Tan, Grace Chain, Muhammad Faizal Zulkifli, Jonathan Wee Kent Liew, Kathryn Vasquez, Man Ling Chau, Youming Ng, Vernon Lee, Shuzhen Sim, Cheong Huat Tan, Lee Ching Ng

**Affiliations:** a Environmental Health Institute, National Environment Agency, Singapore; b Department of Biological Sciences, National University of Singapore, Singapore; c Lee Kong Chian School of Medicine, Nanyang Technological University, Singapore; d Saw Swee Hock School of Public Health, National University of Singapore and National University Health System, Singapore; e Communicable Diseases Agency, Singapore; f School of Biological Sciences, Nanyang Technological University, Singapore

**Keywords:** RCT, Dengue control, *Wolbachia*, IIT-SIT, *Aedes aegypti*

## Abstract

**Background:**

Wild-type female *Aedes aegypti* which mate with *Wolbachia*-infected male *Aedes aegypti* (*Wolbachia-Aedes*) produce non-viable eggs due to cytoplasmic incompatibility. Sustained releases of *Wolbachia*-infected male *Aedes aegypti* can suppress wild-type mosquito populations, but robust evidence of its efficacy in reducing dengue incidence is needed.

**Methods:**

We conducted a large-scale cluster-randomised controlled trial involving releases of male *w*AlbB-infected *Ae. aegypti* mosquitoes (*w*AlbB-SG) for dengue control in Singapore. Mosquitoes were irradiated to sterilise potential residual females. Fifteen clusters were randomised: 8 clusters received deployments of male *w*AlbB-SG infected *Ae. aegypti* (intervention clusters, n = 393,236) and 7 clusters received no deployments (control clusters, n = 331,192). All clusters practised baseline vector control measures. We assessed the protective efficacy of *Wolbachia-Aedes* to reduce dengue incidence using a differences-in-differences framework. The trial was registered with ClinicalTrials.gov (Identifier: NCT05505682) on 2022-08-18.

**Findings:**

In the pre-intervention period, dengue incidence per 100,000 was similar across control (18.63) and intervention (18.14) arms. During the intervention period, reported dengue incidence was 11.31 and 36.57 cases per 100,000 residents in intervention and control arms, respectively. Six months after releases began, intervention clusters showed reductions in total dengue incidence, ranging from 27.6% (95% CI: −35.4% to 61.3%) to 83.8% (95% CI: 50.4%–94.7%). This protective effect varied across time, with some attenuation observed during the 2023 inter-epidemic period. We estimated that 2351 cases (95% CI: 1968–2735) were averted over the period of the trial, relative to 1037 dengue cases reported in the intervention arm. Reductions in epidemiologically-linked dengue incidence were greater, ranging from 60.9% (95% CI: 21.3%–80.6%) to 99.8% (95% CI: 99.2%–99.9%).

**Interpretation:**

Our results demonstrate the high efficacy of *Wolbachia-Aedes* in reducing dengue incidence.

**Funding:**

Ministry of Finance, Singapore.

**Research in context**

**Evidence before this study**

We searched PubMed from inception up to April 13, 2026 for published randomised controlled trials of *Wolbachia*-infected *Aedes* mosquitoes for dengue control, using search term “(wolbachia) AND (trial) AND (dengue) AND (male) AND (Aedes)”. The search identified 44 studies, of which two articles reported epidemiological results from randomised controlled trials. One was a cluster-randomised controlled trial in Indonesia using *Wolbachia* introgression approach, which reported 77.1% protective efficacy against virologically confirmed dengue. The other was a cluster-randomised controlled trial in Singapore evaluating release of male-only *Wolbachia*-infected mosquitoes, which reported 71%–72% of reduction in the dengue risk.

**Added value of this study**

Although previous randomised controlled trials of *Wolbachia*-infected mosquitoes have shown successful reductions in dengue transmission, evidence on the efficacy of releasing male-only *Wolbachia*-infected *Ae. aegypti* remains scarce. This study provides the randomised controlled trial evidence that release of male-only *Wolbachia*-infected *Ae. aegypti* can substantially reduce reported dengue incidence and, consequently, healthcare utilisation stemming from dengue.

**Implications of all the available evidence**

Our study provides supportive evidence that the release of male-only *Wolbachia*-infected *Ae. aegypti* reduces dengue incidence and can be a complimentary tool for vector control.

## Introduction

Dengue is an *Aedes*-borne acute viral syndrome caused by the dengue virus. An estimated 50 million to 100 million symptomatic cases occur globally each year,^1^ with outbreaks causing a surge in case numbers and resulting in considerable pressure on healthcare systems. A promising approach to vector control is targeting the adult mosquito population through *Wolbachia*-mediated incompatible insect technique-sterile insect technique (IIT-SIT). This involves the release of only male *Wolbachia*-infected *Aedes aegypti* mosquitoes treated with irradiation (hereafter referred to as *Wolbachia-Aedes*).^2^ In Singapore, past phases of the field trials have comprehensively described and evaluated the behaviour, efficacy to induce cytoplasmic incompatibility, competitiveness, and fitness of *Wolbachia-Aedes* or *w*AlbB-SG, a localised *Aedes aegypti* line stably infected with *Wolbachia w*AlbB strain under field conditions.^3^ Production and release strategies for IIT-SIT have also been optimised to enable consistent releases over high-rise, densely populated environments. Effectiveness to reduce *Aedes aegypti* abundance and dengue risk/incidence has also been previously demonstrated in a long-standing field trial in 4 townships through retrospective assessment.^3^, ^4^, ^5^, ^6^, ^7^

This manuscript complements our first primary endpoint recently published,^8^ which showed 71%–72% of protective efficacy of the intervention on dengue risk. Here, we report the results of the epidemiological efficacy of *Wolbachia-Aedes* deployments using IIT-SIT to reduce dengue incidence, in the same cluster randomised controlled trial (cRCT). By building upon prior work in the same study setting, this trial provides estimates of the epidemiological efficacy of IIT-SIT.^3^, ^4^, ^5^, ^6^^,^^9^^,^^10^ These two endpoints together provide complementary perspectives. The first primary endpoint focuses on the test positive rate of suspect dengue, quantifying individual level of dengue risk; the current manuscript examines the reduction in reported dengue incidence—reflecting healthcare utilisation—and distinguishes the impact on epidemiologically linked (local transmission) vs sporadic cases.

## Methods

### Trial design

This is an unblinded, two-arm, cluster-randomised controlled trial, and was led by the Environmental Health Institute, National Environment Agency, Singapore. The protocol was previously published.^9^^,^^10^ This study reports the efficacy of *Wolbachia-Aedes* deployment on dengue incidence.

The trial had two primary endpoints, each planned as independent, conceptually distinct outcomes using different datasets. The first primary endpoint measures individual-level dengue risk, measuring the odds ratio of exposure to *Wolbachia-Aedes* release among residents with confirmed dengue compared to dengue negative controls. Evaluation of the first primary endpoint relied on a nationally representative dataset of residents with suspected dengue illness who were tested for dengue, collated from all major diagnostic laboratories through primary care clinics and hospitals.^8^ The second primary endpoint measures cluster-level dengue incidence, contrasted between the *Wolbachia* intervention arm with the control arm with no *Wolbachia* intervention.

The second primary endpoint of dengue incidence is reported here. Evaluation of this endpoint relied on the national dengue surveillance database. As all suspected and laboratory-confirmed cases of dengue are legally notifiable to the Communicable Diseases Agency (CDA),^11^ the registry comprised all reported cases of dengue over the trial duration ascertained via clinical diagnoses or test confirmation. Confirmatory diagnostic testing for dengue is widely available across healthcare settings in Singapore, with results requested at notification. For dengue, NS1 tests are widely utilised in primary care^12^ with additional tests (e.g., PCR) widely available in laboratories.^13^ This enabled systematic capture of dengue cases among those who present to the healthcare system with symptoms. Among all notified dengue cases, around 85% are confirmed using an internally controlled RT-qPCR assay or dengue non-structural protein 1 (NS1) IgM duokit as diagnostic assays,^12^^,^^13^ both of which have been validated for high sensitivity and specificity in the Singapore setting,^12^^,^^14^ with good positive detection rates from the 1st to 15th day of fever onset.^15^ The sensitivity of Singapore's national dengue surveillance system is further evidenced by a reporting rate of 1 in 6, as derived from local seroprevalence data,^16^ despite majority of dengue infections being asymptomatic. Sex and age data of individuals were obtained from government records.

### Ethics

This trial was approved by the Ministry of Health and the National Environment Agency, Singapore. The National Environment Agency Bioethics Review Committee (i.e., Institutional Review Board) has reviewed this trial protocol and approved the exemption of the trial from formal bioethics review (IRB reference number: IRB024), as it is not considered human biological research. The data is routinely collected as part of routine dengue surveillance under the Infectious Disease Act, which exempts the need for informed consent. The trial is registered on ClinicalTrials.gov (NCT05505682, Date of registration: 2022-08-18).

### Clusters characterisation

Trial clusters consisted of urban areas of 12.0 km^2^ comprising high-rise public housing apartments with a population of approximately 724,428 residents. The trial sites comprised 15 clusters of approximately 0.8 km^2^ in size each with historically high dengue incidence. Each cluster had geographic borders that would minimise mosquito dispersal between clusters, using geographic borders such as major roads/highways/water bodies to form cluster boundaries. These borders limit wildtype mosquito migration into designated clusters as well as migration of *Wolbachia-Aedes* from intervention to non-intervention areas. In the absence of geographic borders, adjacent areas to intervention clusters within a 300-m radius were designated as buffer release areas, where male *Wolbachia-Aedes* were also released. Buffer release areas were not considered as part of intervention area and were excluded from both the study population and computation of dengue case counts. All clusters (inclusive of buffer areas) were kept at least 700 m apart.

### Randomisation

Constrained randomisation was conducted to alleviate chance imbalance in historical dengue incidence between intervention and control clusters (See Supplementary Information). This was conducted by only randomly selecting one allocation of intervention and control arm clusters among all possible allocations of clusters to intervention and control arms which were balanced in historical dengue risk.

### *Wolbachia-Aedes* deployment and monitoring *Aedes* population suppression

1–6 male *Wolbachia Aedes* were released twice weekly (weekdays, 0630–1100 h) per resident in intervention clusters on the ground, mid, and high floors of housing estates. Within designated buffer areas, male *Wolbachia-Aedes* were also released twice a week on the ground floors of housing estates. Where releases in buffer areas could not be performed (e.g., schools), releases were conducted at the boundary between such areas and the intervention clusters. Production of male *Wolbachia*-*Aedes* comprised sex-separation of male mosquitoes for release, with SIT adopted using low-dose irradiation to sterilise potential residual females during releases of *Wolbachia*-*Aedes* males to reduce the possibility of releasing fertile females (See Supplementary Information for full details on production and release). Intervention clusters received *Wolbachia-Aedes* releases since Epidemiological Week (EW) 30 2022 to EW37 2022. The trial concluded in EW37 2024.

### Endpoint

The endpoint considered here was monthly reported dengue cases in each cluster as measured using the national dengue surveillance database (monthly dengue incidence). The unit of analysis was monthly, cluster-level dengue incidence with the population size included as an offset in the statistical analysis. The population size—estimated as the number of dwelling units multiplied by four—is fixed at the start of the intervention (year 2022). Comparisons were made between intervention versus control clusters. All laboratory confirmed cases notified to the national dengue surveillance system were included in the analysis. As post-hoc exploratory analyses, we further analysed linked dengue incidence, where epidemiologically-linked dengue cases were defined based on local operational criteria, and were cases which have onset within 14 days and are located within 150 m of each other based on residential and workplace addresses.^17^ Sporadic cases are cases which are not linked dengue cases.

### Statistics

The statistical analysis plan was published previously and described in full detail in the protocol (See Supplementary Information). Formal sample size determination was performed for the first primary endpoint, with an estimated 1600 test-negative controls and 400 dengue-positive cases needed to detect a 50% reduction in dengue risk with 80% power. The protocol states that data collection will continue for at least 24 months or longer if needed to reach the minimum sample size required for intention-to-treat analysis for the first primary endpoint. The study duration of the second primary endpoint followed power calculations based on the first primary endpoint (24 months). The second primary endpoint relied on the routine dengue surveillance data (reported cases), rather than the enrolment of individual participants, inclusion and exclusion criteria were not applicable. The study was non-blinded. Interim analyses were done after 12 months of intervention. The baseline characteristics were well-balanced between study arms (Table 1), so the weighted differences-in-differences was not implemented. We used a differences-in-differences framework,^18^ which can appropriately account for the dynamic impact of intervention (i.e., time taken for *Wolbachia-Aedes* to induce effect on dengue incidence, calendar-time differences in intervention-efficacy due to epidemic/inter-epidemic periods of transmission), as well as the staggered adoption of intervention in clusters randomised to intervention arm. Briefly, we estimated the cohort-specific average treatment effect on the treated *δ*_*e*,*l*_ (i.e., the impact of *Wolbachia-Aedes* treatment on dengue incidence rates in intervention clusters, in some time-interval). Cohorts here pertain to the group of clusters adopting treatment at same calendar time. The following specification was used:

$$\log\left(E\left(Y_{i,t}|.\right)\right)=\alpha_{i}+\lambda_{t}+\sum_{e\notin C}\sum_{l\neq -1}\delta_{e,l}\left(1\left\{E_{i}=e\right\}\cdot \;D_{i,t}^{l}\right)+\log\left(\text{populatio}n_{i}\right)+\epsilon_{i,t}$$

where *Y*_*i*,*t*_ denotes monthly dengue incidence at site *i* at calendar time *t*, *α*_*i*_ and *λ*_*t*_ the site and time fixed effects respectively, *e* denotes the time which a group of clusters adopt treatment, while *C* is the set of intervention adoption times for the control group (i.e., *C* = {*∞*}). *E*_*i*_ is the time for some site *i* to initially receive *Wolbachia-Aedes,* $D_{i,t}^{l}$ denotes an indicator variable for the site *i* to be *l* periods away from the initial treatment date at some calendar time *t*. Treatment effects are estimated for all periods except *l* = −1, that is, the latest period prior to the start of the intervention adoption date. *Log*(population_*i*_) is the offset to account for differences in at-risk population size in each cluster. *ϵ*_*i*,*t*_ is an error term. The quasipoisson distribution was assumed due to the count nature of incidence data with overdispersion, with the log-link function employed. We estimated sample weights *Pr*⁡{*E*_*i*_ = *e*|*E*_*i*_∈[−*l*,*T* − *l*]} which denote the proportion of clusters in each treatment cohort which experiences at least *l* periods relative to *Wolbachia-Aedes* treatment. Following that, we form the interaction-weighted estimator using estimates from the two former steps:

$${\hat{v}}_{g}=\frac{1}{|g|}\;\sum_{l\in g}\sum_{e}{\hat{\delta }}_{e,l}\hat{\mathrm{Pr}}\;\left\{E_{i}=e|E_{i}\in \left[-l,T-l\right]\right\}$$

**Table 1:** Summary statistics for intervention/control clusters with mean and 95% confidence interval.

Arm	Control		Intervention	
	Pre-intervention	During intervention	Pre-intervention	During intervention
Mean dengue incidence (per 100,000)	18.63 (16.06, 21.19)	36.57 (28.20, 44.93)	18.14 (16.06, 20.22)	11.31 (8.51, 14.12)
Mean Temperature (°C)	27.97 (27.92, 28.02)	28.03 (27.89, 28.17)	28.07 (28.02, 28.12)	28.11 (27.98, 28.25)
Mean wind speed (km/h)	8.14 (7.99, 8.29)	8.21 (7.88, 8.54)	8.85 (8.70, 9.01)	8.04 (7.78, 8.30)
Mean Normalised Difference Vegetation Index	0.32 (0.32, 0.32)	0.32 (0.32, 0.32)	0.32 (0.32, 0.32)	0.32 (0.32, 0.32)
% of area within 300 m of waterbody	0.31 (0.29, 0.34)	0.31 (0.27, 0.36)	0.23 (0.22, 0.24)	0.23 (0.20, 0.26)
Public housing age based on recent data (years)	34.70 (34.25, 35.16)	34.70 (33.77, 35.63)	32.46 (31.98, 32.95)	32.52 (31.48, 33.56)
Public housing height based on recent data (m)	38.12 (37.65, 38.59)	38.12 (37.16, 39.09)	34.55 (34.29, 34.81)	34.61 (34.07, 35.15)
Public housing price per m^2^ (S$)	4780.67 (4729.12, 4832.23)	5998.10 (5882.23, 6113.96)	4286.37 (4252.37, 4320.37)	5512.64 (5451.75, 5573.53)
Length of drainage network within spatial unit (m)	84.77 (77.73, 91.80)	84.77 (70.38, 99.16)	81.17 (74.28, 88.06)	82.92 (68.15, 97.70)
Forest %	0.01 (0.01, 0.02)	0.01 (0.01, 0.02)	0.00 (0.00, 0.00)	0.00 (0.00, 0.00)
Grass %	0.00 (0.00, 0.00)	0.00 (0.00, 0.00)	0.00 (0.00, 0.00)	0.00 (0.00, 0.00)
% of total vegetation	0.02 (0.02, 0.02)	0.02 (0.02, 0.02)	0.03 (0.02, 0.03)	0.02 (0.02, 0.03)
Daily total precipitation (mm)	6.51 (6.27, 6.74)	8.08 (7.55, 8.61)	6.13 (5.92, 6.34)	7.39 (6.87, 7.91)
Relative humidity (%)	80.96 (80.83, 81.08)	81.84 (81.48, 82.19)	80.92 (80.80, 81.03)	81.98 (81.64, 82.32)

Pre-intervention is dated back to January 2014. Statistics presented for the intervention group were based on their release and non-release periods. For the control group, intervention period was from 2022 August onwards until end of the trial. The overlapping 95% confidence intervals between control and intervention sites suggest that the difference in characteristics between the two arms was not statistically significant.

m: metres; °C: degrees Celsius; mm: millimetres; km/h: kilometres per hour.

To examine the effect of the intervention for every 3 months since the intervention began, we binned our estimates of intervention efficacy to 3-month intervals. Here, *g* denotes the bin of periods relative to start of *Wolbachia-Aedes* treatment. The null hypothesis was that there was no intervention efficacy in dengue incidence between intervention and control clusters, in the intervention period. Intervention efficacy per 3-month interval since the intervention began was calculated as $100\times \left(1-\exp\left({\hat{v}}_{g}\right)\right)$ (See Supplementary Information for derivation and SI Table 1). Standard errors were clustered at the cluster level and small-sample corrections were applied to the cluster-robust variance covariance matrix considering both effective number of parameters and cluster count. Confidence intervals were then based on a student's t distribution with degree of freedom as number of clusters minus one.

Raw cases averted were calculated as the difference in incidence rates between intervention and control arms, multiplied by the intervention population size. Incidence rate was defined as the number of cases divided by the corresponding population size. The standard error of the estimated cases averted was calculated from the square root of the sum of variances in intervention and control arms multiplied by the intervention population size, where the variance for each arm was approximated as the number of cases divided by the square of the population size. The 95% confidence intervals were then estimated using the normal approximation as the estimated cases averted ± 1.96 times the standard error.

A large battery of robustness checks was conducted to examine the internal validity of our analytical framework. We (**1**) examined if estimated intervention efficacies would differ, by inclusion or exclusion of time-varying covariates (i.e., monthly mean temperature, monthly mean wind speed, monthly mean daily total precipitation, monthly mean relative humidity, public housing price per m^2^), (**2**) used the Goodman-Bacon decomposition to examine if the staggered adoption setting led to problematic comparisons in our differences-in-differences identification setup.^18^ (**3**) We conducted in-time placebo tests. We examined the estimated intervention efficacies in the time before interventions began using models with and without covariates, to see if our analytical framework results in spurious detection of intervention efficacies. (**4**) We conducted in-space placebo tests. We reassigned the intervention allocation to control units while removing the actual intervention clusters from the analysis. We iteratively took 1, 2 or 3 control clusters to be allocated to a placebo intervention arm and used the remaining 6, 5 or 4 control clusters as the remaining control arm. We repeated all analytical procedures to obtain the average treatment effect and intervention efficacy, for each time-period since intervention. (**5**) We conducted parallel trends tests to ascertain that parallel trends were observed in the pre-intervention period. All analyses were repeated for total, linked and sporadic dengue incidence rates, with the exception of the in-space placebo analysis for sporadic dengue incidence due to low case counts.

### Role of funders

The funders had no role in the study design, data collection, analysis of the data, interpretation, in the preparation or approval of the manuscript, or in the decision to submit the manuscript for publication.

## Results

**Fig. 1:**
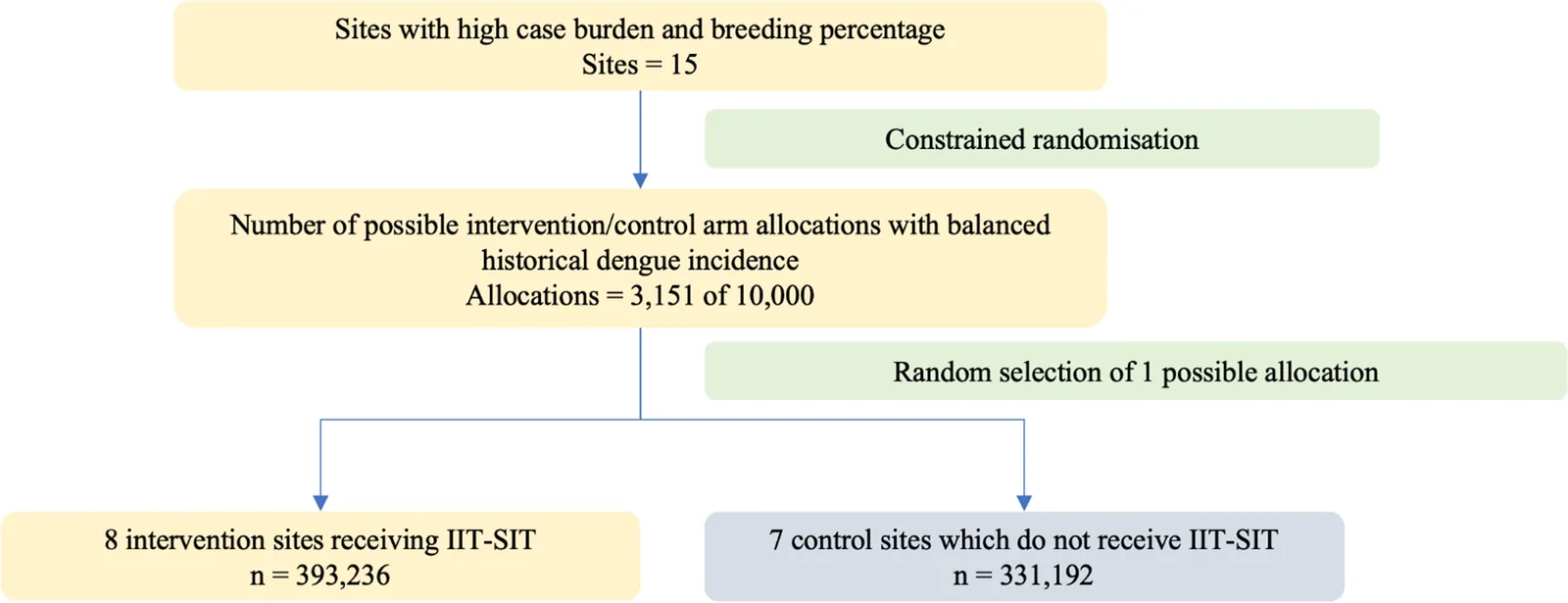
Trial cluster flowchart. Breeding percentage is the number of *Aedes aegypti*–positive habitats over the total number of *Aedes*-positive habitats.

Eight clusters were randomly assigned to receive open-label *Wolbachia-Aedes* deployments (intervention clusters), and 7 clusters were assigned to receive no deployments (control clusters). No placebo was used in control clusters. All clusters practiced baseline vector control measures (Fig. 1). A total of 393,236 and 331,192 residents were allocated to the intervention and control arms respectively and included in statistical analysis (Fig. 1).

### Suppression of *Aedes aegypti* populations and dengue incidence rates in intervention clusters

**Fig. 2:**
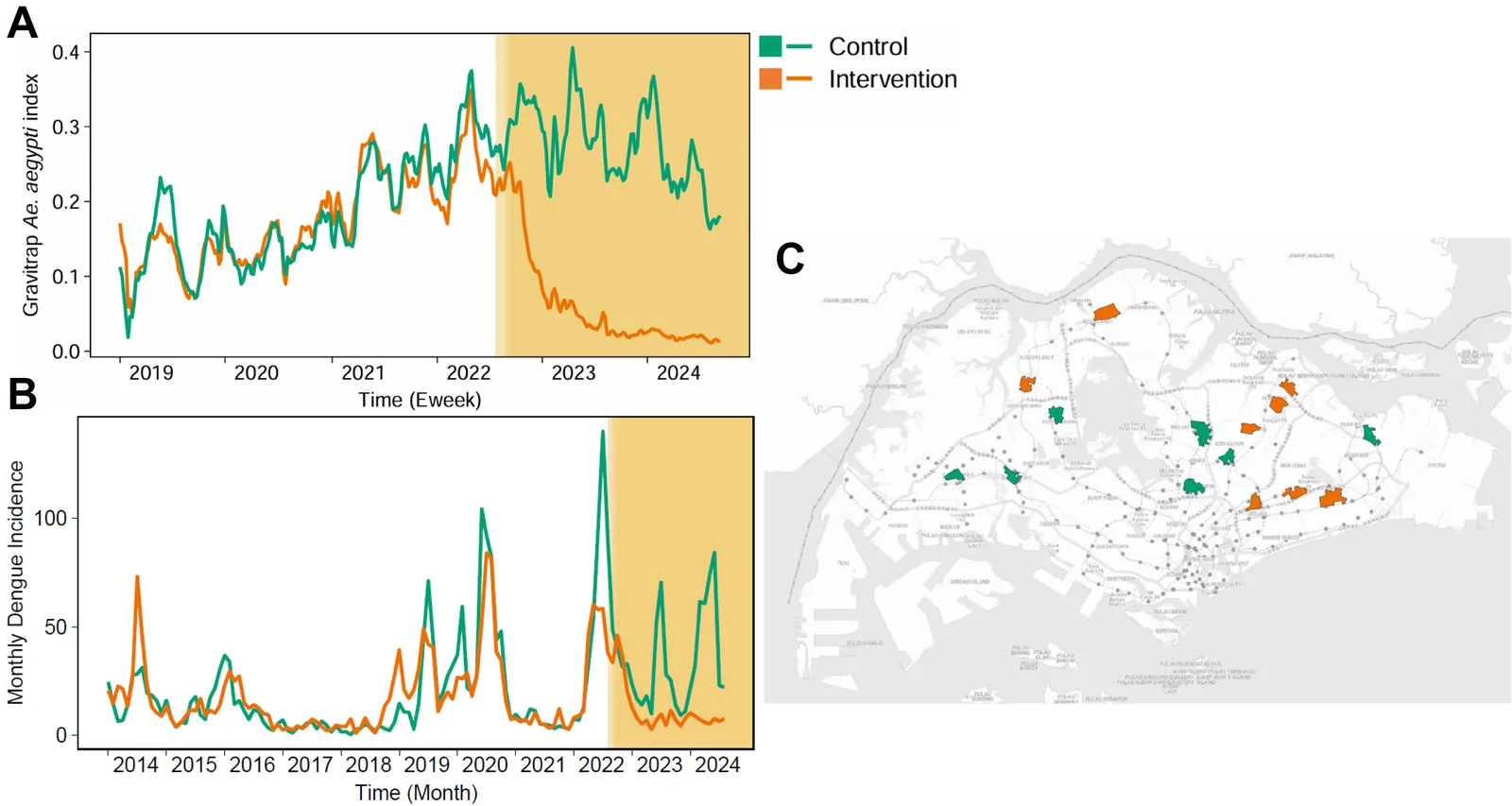
(A) Gravitrap *Aedes aegypti* index, which proxies for adult *Aedes aegypti* abundance, for intervention and control arms, in the pre- and during *Wolbachia-Aedes* release periods (B) Monthly dengue incidence rates per 100,000 individuals for intervention and control arms, in the pre- and during *Wolbachia-Aedes* release periods. Highlighted portions denote the trial study period in panels (A) and (B). (C) Spatial visualisation of RCT intervention and control clusters. Panel (A) and (C) are also shown in *Lim* et al.^8^

Suppression of adult wild-type *Ae. aegypti* populations was demonstrated across all intervention clusters, with the Gravitrap *Ae. aegypti* Index (GAI) drastically reduced across time. Pre-intervention GAI levels averaged 0.19 in intervention and control clusters, but from 3 months after intervention implementation to the end of the study period, the average intervention cluster GAI plunged to 0.041, while the control site GAI was at an average of 0.277 (Fig. 2A). Monthly dengue incidence rates matched closely between arms historically from 2014 to 2022, with pre-intervention average of 18.14 and 18.63 cases per 100,000 residents in intervention and control clusters respectively (Fig. 2B, Table 1). After intervention implementation, incidence in intervention clusters declined to 11.31 cases per 100,000 residents and remained suppressed throughout the study period, while control cluster incidence rose to 36.57 cases per 100,000 residents per month (Fig. 2B, Table 1).

### Baseline trial characteristics

Prior to the trial, historical dengue incidence was well-matched. There were no major differences in other baseline characteristics between arms, pre- and during the intervention (Table 1). In both arms, the sex and age distribution of reported cases were similar between the pre-intervention and intervention periods, and the population demographics were comparable across clusters (SI Tables 2–4, and SI Fig. 1).

### Suppression of dengue incidence rates over time

**Fig. 3:**
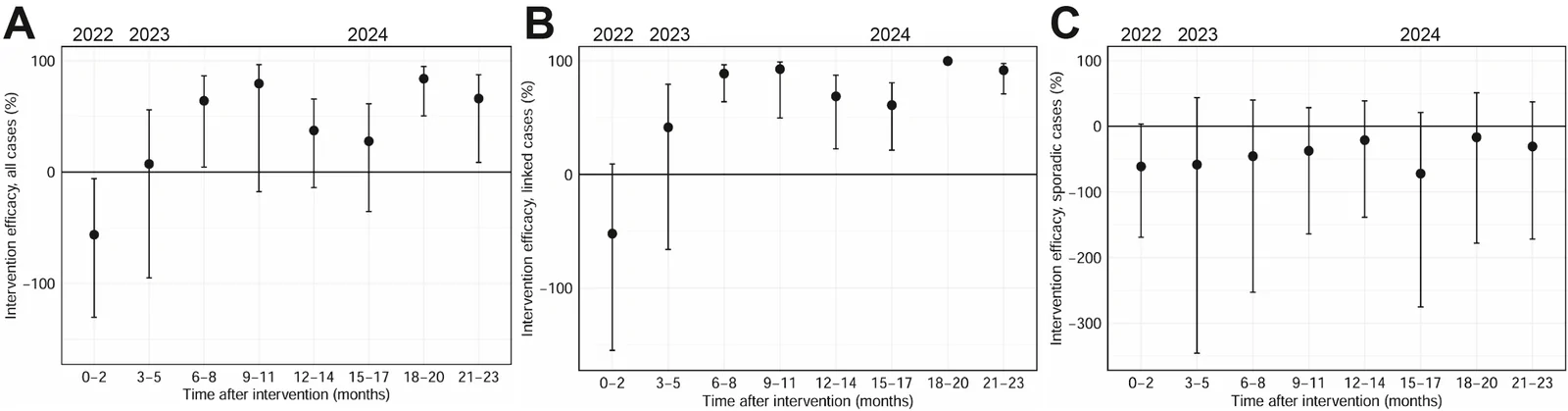
Intervention efficacy of release of *Wolbachia-Aedes* on (A) dengue incidence rates (B) epidemiologically-linked dengue incidence rates (C) sporadic dengue incidence rates over each 3-month period since the intervention began. Intervention efficacy was defined as the percentage reduction in dengue incidence and calculated as $100\times \left(1-\exp\left({\hat{v}}_{g}\right)\right)$ (See Supplementary Information for derivation). Points represent point estimates and error bars represent 95% confidence interval. The study included 7 control clusters and 8 intervention clusters.

Intervention clusters which received *Wolbachia-Aedes* intervention showed increasing reductions in dengue incidence rates when subject to longer periods of intervention (Fig. 3). In the 0–5 months following release, intervention efficacies were not positive or significant. Whereas 6 months after intervention implementation, reductions in dengue incidence in intervention clusters ranged from 28% to 84%, though not all estimates were statistically significant (Fig. 3A: 6–8 months: 63.9% (95% CI: 4.5%–86.4%), 9–11 months: 79.4% (95% CI: −17.6% to 96.4%), 12–14 months: 37.3% (95% CI: −13.9% to 65.5%), 15–17 months: 27.6% (95% CI: −35.4% to 61.3%), 18–20 months: 83.8% (95% CI: 50.4%–94.7%), 21+ months: 65.9% (95% CI: 8.6%–87.3%).

Intervention efficacies for epidemiologically-linked dengue cases were more pronounced than that of total dengue incidence rates. Linked dengue cases accounted for 76% of all dengue cases. After 6 months of releases, reductions in linked dengue cases ranged from 61% to 99% (Fig. 3B: 6–8 months: 88.7% (95% CI: 63.9%–96.5%), 9–11 months: 92.6% (95% CI: 49.6%–98.9%), 12–14 months: 68.6% (95% CI: 22.6%–87.3%), 15–17 months: 60.9% (95% CI: 21.3%–80.6%), 18–20 months: 99.8% (95% CI: 99.2%–99.9%), 21+ months: 91.6% (95% CI: 70.8%–97.6%). There was an increase, though not significant, in sporadic cases over the study period in intervention clusters (Fig. 3C). A dip in intervention efficacy across dengue case classifications was apparent 12–17 months after intervention implementation (Fig. 3) coincided with a period of low transmission in 2023 (See Fig. 2B).

We also computed the raw difference in dengue incidence rates between intervention and control clusters, and normalised this difference by the population size in intervention clusters to obtain the total number of cases averted. Overall, we estimated that around 2351 cases (95% CI: 1968–2735) were averted over the period of the trial, relative to 1037 dengue cases reported in the intervention arm in the study period (Table 2).

**Table 2:** Raw reductions in dengue cases, epidemiologically-linked dengue cases, and sporadic dengue cases over each 3-month period since the intervention began in intervention clusters.

Exposure period	Total cases averted (95% CI)	Linked cases averted (95% CI)	Sporadic cases averted (95% CI)
0–2 months	**205.85 (137.39, 274.31)**	**201.48 (136.64, 266.32)**	4.37 (−17.59, 26.32)
3–5 months	**213.89 (169.82, 257.96)**	**217.40 (178.29, 256.51)**	−3.51 (−23.82, 16.80)
6–8 months	**112.98 (81.31, 144.64)**	**99.98 (76.03, 123.94)**	12.99 (−7.72, 33.70)
9–11 months	**564.41 (506.62, 622.19)**	**571.98 (518.49, 625.47)**	−7.57 (−29.43, 14.29)
12–14 months	**225.58 (184.75, 266.42)**	**207.34 (173.59, 241.09)**	18.24 (−4.75, 41.23)
15–17 months	**57.79 (23.08, 92.50)**	**52.36 (27.52, 77.20)**	5.43 (−18.82, 29.67)
18–20 months	**437.37 (386.38, 488.36)**	**435.06 (387.95, 482.17)**	2.31 (−17.19, 21.81)
21–23 months	**533.79 (479.04, 588.54)**	**512.55 (462.74, 562.36)**	21.24 (−1.50, 43.98)
Total	**2351.66 (1968.39, 2734.92)**	**2298.16 (1961.26, 2635.06)**	53.50 (−120.82, 227.81)

The intervention period in the control arm started from the earliest intervention start date.

Cases averted is calculated as the (case incidence rate in control arm - case incidence rate in treatment arm) ∗ population size in treatment arm.

Bolded figures denote estimates where 95% CIs do not cross 0.

### Robustness checks

A large battery of robustness checks was conducted to ascertain the validity of intervention efficacy estimates. (**1**) Incorporation or removal of covariates from our specification did not significantly affect intervention efficacy estimates (SI Table 5) (**2**) Goodman-Bacon decompositions indicated a small number of problematic comparisons when using the standard two-way-fixed effects difference-in-difference design, which justified further the use of the interaction-weighted estimator to obtain intervention efficacy estimates (SI Table 6). (**3**) In-time placebo checks demonstrated that no intervention efficacies were estimated in the pre-intervention period in intervention clusters for total and linked cases (SI Table 7). However, it produced some, mostly negative, spurious intervention effects for sporadic cases as an outcome, in the pre-intervention period (SI Table 7). This may be due to the small number of sporadic cases observed across the pre-intervention period and the multiple pre-intervention periods for which the outcome was tested against. (**4**) In-space placebo checks demonstrated that estimated placebo intervention efficacies were mostly centred around 0 (SI Figs. 2–5). (**5**) We detected parallel trends in dengue incidence in intervention and control arms in the pre-intervention period based on pre-intervention intervention efficacy (SI Figs. 6–8). See Supplementary Information for full results.

## Discussion

In this cluster-randomised controlled trial comprising over 700,000 residents, we assessed the efficacy of *Wolbachia-Aedes* to reduce dengue incidence rates. Analysis indicates that mosquito abundance was suppressed in the *Wolbachia-Aedes* release sites due to cytoplasmic incompatibility, resulting in reductions in dengue incidence rates over time. From 6-months after intervention started, reductions in dengue incidence rates in intervention clusters ranged from 27.6% to 83.8% across different time intervals. High intervention efficacy coincided with the epidemic season and the efficacy attenuated between 12 and 17 months after release, overlapping with 2023 inter-epidemic period (Fig. 2B).

Reductions in epidemiologically-linked dengue incidence rates were far greater (60.9%–99.8%) than total dengue incidence rates. Epidemiologically linked cases are defined by geographical proximity and symptom onset dates, based on local vector control operational criteria, therefore they should be interpreted as a proxy, rather than a confirmed measure of linked transmission as cases meeting these criteria may belong to separate transmission chains that happen in close residential proximity and a study incorporating virus genetic data suggests that transmission in Singapore can occur over longer distance.^19^ As the cases are tagged to residential addresses, sporadic cases are more likely associated with infections acquired outside of the case's residence, for example in the workplace, during travel, or through other activities elsewhere. The improved efficacies derived without sporadic cases suggest that potential imports of dengue cases into intervention clusters may be diluting estimated intervention efficacies of total dengue incidence rates. Future studies could investigate how the release of *Wolbachia-Aedes* changes the spatial transmission dynamics in Singapore. In totality, estimated intervention efficacies highlight that suppressing the main vector species of dengue through *Wolbachia-Aedes* can lead to substantial decrease in dengue incidence.

Our study has several strengths. Firstly, we employed a constrained-randomisation protocol to ensure historical balance in dengue incidence between intervention and control arms, and analogously, close temporal trends between the two arms (Fig. 1, Table 1). Secondly, we leveraged the high capture rate of dengue cases in Singapore using the national dengue disease surveillance system which confirmed the majority of cases based on highly sensitive and specific dengue tests, alleviating potential misclassification error. This system comprehensively covered all intervention and control clusters pre- and during the intervention to evaluate intervention efficacy, and also provided sufficient historical information to internally validate our analytical framework. Thirdly, by re-defining our outcomes based on epidemiologically-linked or sporadic dengue incidence rates, we could account for potential importation of cases into intervention clusters and triangulate the underestimation of intervention efficacies using total dengue incidence rates. The consistently low or negative intervention efficacies estimated for sporadic cases suggests that a proportion of people might have acquired the disease elsewhere. It also implies that estimated intervention efficacies under total dengue incidence rates were conservative and efficacies could further improve when the programme expands to more areas. Fourthly, our analytical framework to obtain intervention efficacy estimates alleviates many of the key issues regarding the use of differences-in-differences (DiD) under our trial setup (staggered adoption setting) by removing problematic comparisons which can potentially be made under the two-way fixed effects DiD framework. Namely, treatment effects can be time-varying when evaluating *Wolbachia-Aedes* interventions, due to the time required to induce mosquito suppression, or when the trial encompasses both national epidemic and inter-epidemic periods of transmission. This may lead to biases when using the conventional DiD setup, as comparisons will be made between earlier and later treated units to obtain the average treatment effect of *Wolbachia-Aedes*. By being able to also estimate intervention effects by time-since-intervention, we also provided valuable information on the time required to induce a high level of reduction in dengue incidence rates in intervention clusters, and the time-varying effect of the intervention across interepidemic and epidemic periods of transmission. Lastly, we employed a large battery of robustness checks also allay concerns that our inferential framework leads to spurious intervention efficacy estimates.

Nevertheless, we note that there may be some limitations associated with the study. Firstly, while the local surveillance system for dengue is extensive, with similar levels of test-positive dengue patients and incidence rates in the pre-intervention period, case under-ascertainment cannot be ruled out given its reliance on passive surveillance. Differences in medical care-seeking behaviour, testing practices across clusters may have introduced ascertainment bias. Evaluation of the first primary endpoint addressed this concern through the use of a test-negative design.^8^ Secondly, epidemiologically-linked dengue cases defined using local operational criteria were used to approximate infections occurring within the study areas. However, these classifications serve only as proxies for infections acquired at the place of residence and may be susceptible to measurement error. Future studies can incorporate finer-scale human mobility data to more accurately attribute infections to study areas and better estimate the true efficacy of *Wolbachia-Aedes* in study areas. Thirdly, population sizes were held constant at their 2022 level rather than dynamic over the study period as it was derived from the number of dwelling units in each cluster. Although this may not fully capture year-to-year demographic changes, these changes were expected to be small and therefore unlikely to qualitatively affect the findings. Fourthly, the study duration was 24 months following the power calculation for the first primary endpoint, rather than the 31 months specified in the study protocol.^9^ Post-hoc power calculations indicated that a 24-month duration is adequately powered to detect larger intervention efficacies, with powers of 0.53, 0.71, 0.86, 0.95 (n = 1000, *p* < 0.05) for intervention efficacies of 60%, 70%, 80% and 85%, respectively. Lastly, while our analysis comprised a sizeable number of clusters and controlled for a large number of potential anthropogenic and environmental confounders as well as historical dengue incidence, chance imbalances in unobserved confounders between arms cannot be ruled out.

Our efficacy results are consistent with the large body of work on the use of *Wolbachia-Aedes* for mosquito suppression and reducing dengue incidence. Prior entomological studies in Singapore demonstrated that *Wolbachia-Aedes* can suppress *Ae. aegypti* abundance by around 90% with 18 or more months of release,^3^ which concomitantly led to large reductions in both the risk and incidence of dengue in directly treated^5^, ^6^, ^7^ and nearby sites.^4^ Our work also aligns with results on the large reductions in dengue risk (∼72%) conferred from *Wolbachia-Aedes*, as assessed using test-negative data from the same cRCT.^8^ We contribute to this body of work by demonstrating that *Wolbachia-Aedes* can confer significant protective efficacies against dengue incidence over large spatial scales, in a prospective RCT setup.

Suppression using male *Wolbachia-Aedes* can be an effective tool to reduce the incidence of dengue. The strategy is likely to be enhanced by a comprehensive vector-control programme that effectively targets other stages of the mosquito lifecycle, such as source reduction efforts and community engagement. Prior to large scale implementation of *Wolbachia-Aedes* suppression technology in Singapore, a risk assessment concluded that the risks associated with releases of male *Wolbachia-Aedes* mosquitoes are low or negligible.^20^^,^^21^ Two considerations warrant monitoring as part of long-term programme strategies: (1) the potential for *Aedes albopictus* to expand into niches previously dominated by *Ae. aegypti* populations, which could have implications for Zika or chikungunya transmission, but evidence of this in Singapore remains limited,^22^ and (2) a decline in community and stakeholder vigilance in source reduction, which could create breeding habitats for *Ae. albopictus*. These ecological and behavioural considerations should be factored into the design and evaluation of long-term programme strategies.

## Contributors

Conceptualisation and design of study: JTL, CSC, KV, SS, CHT, NLC, VL. Data analysis: JTL, CC, DM, YZ. Interpretation of data: JTL, CC. Visualisation: JTL, CC, LYT, GC. Data collection: YN, LYT, LD, CL, GC, MFZ, KV, SS, JWKL, BD, CSC. Project administration: CSC, CC, YN, BD, MLC, SS, CHT, LCN. Funding acquisition: SS, CHT, LCN, MLC. Writing (original draft): JTL, CC. All authors have read, edited and approved the final version of the manuscript. CC and DM have accessed and verified the data.

## Data sharing statement

Permission to access dengue case data should be obtained from the Communicable Diseases Agency, Singapore. Request for permission of use should be directed to Chang_chia-chen@nea.gov.sg.

## Declaration of interests

The authors declare no competing interests.

## Appendix A Supplementary data

Supplementary Material

Protocol

## References

[1] Cattarino L., Rodriguez-Barraquer I., Imai N., Cummings D.A.T., Ferguson N.M. (2020). Mapping global variation in dengue transmission intensity. Sci Transl Med.

[2] Kittayapong P., Ninphanomchai S., Limohpasmanee W., Chansang C., Chansang U., Mongkalangoon P. (2019). Combined sterile insect technique and incompatible insect technique: the first proof-of-concept to suppress Aedes aegypti vector populations in semi-rural settings in Thailand. PLoS Negl Trop Dis.

[3] Bansal S., Lim J.T., Chong C.-S. (2024). Effectiveness of Wolbachia-mediated sterility coupled with sterile insect technique to suppress adult Aedes aegypti populations in Singapore: a synthetic control study. Lancet Planet Health.

[4] Lim J.T., Mailepessov D., Chong C.S. (2025). Adjacent spillover efficacy of Wolbachia for control of dengue: emulation of a cluster randomised target trial. BMC Med.

[5] Lim J.T., Mailepessov D., Chong C.-S. (2024). Assessing Wolbachia-mediated sterility for dengue control: emulation of a cluster-randomized target trial in Singapore. J Trav Med.

[6] Lim J.T., Bansal S., Chong C.S. (2024). Efficacy of *Wolbachia*-mediated sterility to reduce the incidence of dengue: a synthetic control study in Singapore. Lancet Microbe.

[7] Chow J.Y., Geng L., Bansal S. (2024). Evaluating quasi-experimental approaches for estimating epidemiological efficacy of non-randomised field trials: applications in Wolbachia interventions for dengue. BMC Med Res Methodol.

[8] Lim J.T., Chong C.-S., Chang C.-C. (2026). Dengue suppression by Male wolbachia-infected mosquitoes. N Engl J Med.

[9] Ong J., Ho S.H., Soh S.X.H. (2022). Assessing the efficacy of male Wolbachia-infected mosquito deployments to reduce dengue incidence in Singapore: study protocol for a cluster-randomized controlled trial. Trials.

[10] Lim J.T., Mailepessov D., Chong C.-S. (2024). Update to: assessing the efficacy of male Wolbachia-infected mosquito deployments to reduce dengue incidence in Singapore. Trials.

[11] Dengue - National Centre for Infectious Diseases https://www.ncid.sg/Health-Professionals/Diseases-and-Conditions/Pages/Dengue.aspx.

[12] Pok K.-Y., Lai Y.-L., Sng J., Ng L.-C. (2010). Evaluation of nonstructural 1 antigen assays for the diagnosis and surveillance of dengue in Singapore. Vector Borne Zoonotic Dis.

[13] Lai Y.-L., Chung Y.-K., Tan H.-C. (2007). Cost-effective real-time reverse transcriptase PCR (RT-PCR) to screen for Dengue virus followed by rapid single-tube multiplex RT-PCR for serotyping of the virus. J Clin Microbiol.

[14] Gan V.C., Tan L.-K., Lye D.C. (2014). Diagnosing dengue at the Point-of-Care: utility of a rapid combined diagnostic kit in Singapore. PLoS ONE.

[15] Wang S.M., Sekaran S.D. (2010). Early diagnosis of dengue infection using a commercial dengue duo rapid test kit for the detection of NS1, IGM, and IGG. Am J Trop Med Hyg.

[16] Tan L.K., Low S.L., Sun H. (2019). Force of infection and true infection rate of dengue in Singapore: implications for dengue control and management. Am J Epidemiol.

[17] Dengue clusters https://www.nea.gov.sg/dengue-zika/dengue/dengue-clusters.

[18] Goodman-Bacon A. (2021). Difference-in-differences with variation in treatment timing. J Econom.

[19] Yeo Z.Y., Hapuarachchi H.C., Ong J. (2025). Inferring the hidden and long-range dengue transmission routes in Singapore. Mach Learn: Health.

[20] National Environment Agency Wolbachia is safe and natural. https://www.nea.gov.sg/corporate-functions/resources/research/environmental_health_institute/wolbachia-aedes-mosquito-suppression-strategy/wolbachia-is-safe.

[21] Ng L.C., Liew C., Gutierrez R. (2017). How safe is Wolbachia for Aedes control? A risk assessment for the use of male Wolbachia-carrying Aedes aegypti for suppression of the Aedes aegypti mosquito population. Epidemiol News Bull.

[22] Wong W.J., Tan C.H., Verkaik M.G., Ng L.C., Hoffmann A.A., Chong C.-S. (2025). Suppression of Aedes aegypti may not affect sympatric Aedes albopictus populations: findings from two years of entomological surveillance in Singapore. Sci Rep.

