# Supplementary material for: Impact of Wolbachia-mediated incompatible and sterile insect techniques on dengue incidence: a large-scale cluster randomised controlled trial
Source: eBioMedicine. 2026 Sep 10;132:106476. doi: 10.1016/j.ebiom.2026.106476 (PMC13586744; doi:10.1016/j.ebiom.2026.106476)
Supplement: Supplementary file 1 [file mmc2.pdf]

This supplement contains the following items:

1. Original protocol and statistical analysis plan, final protocol and statistical analysis plan and summary of changes.

Final protocol and statistical analysis plan

<u>Protocol and statistical analysis plan</u>

Title: Assessing the efficacy of male *Wolbachia*-infected mosquito deployments to reduce dengue incidence in Singapore

Version: 1.2

Trial registration identifier: This protocol was registered on ClinicalTrials.gov as NCT05505682

Protocol Contributors:

Lee Ching Ng	Environmental Health Institute, National Environment Agency, Singapore
Shuzhen Sim	Environmental Health Institute, National Environment Agency, Singapore
Tan Cheong Huat	Environmental Health Institute, National Environment Agency, Singapore
Chee Seng Chong	Environmental Health Institute, National Environment Agency, Singapore
Judith Chui Ching Wong	Environmental Health Institute, National Environment Agency, Singapore
Chia-chen Chang	Environmental Health Institute, National Environment Agency, Singapore
Yee Ling Lai	Environmental Health Institute, National Environment Agency, Singapore
Kathryn Vasquez	Environmental Health Institute, National Environment Agency, Singapore
Youming Ng	Environmental Health Institute, National Environment Agency, Singapore
Yvonne Wong	Environmental Health Institute, National Environment Agency, Singapore
Yin Xiang Setoh	Environmental Health Institute, National Environment Agency, Singapore
Janet Ong	Environmental Health Institute, National Environment Agency, Singapore
Caleb Lee	Environmental Health Institute, National Environment Agency, Singapore
Lu Deng	Environmental Health Institute, National Environment Agency, Singapore
Li Yun Tan	Environmental Health Institute, National Environment Agency, Singapore
Soon Hoe Ho	Environmental Health Institute, National Environment Agency, Singapore
Grace Chian	Environmental Health Institute, National Environment Agency, Singapore
Lim Jue Tao	Lee Kong Chian School of Medicine, Nanyang Technological University, Singapore<br>Environmental Health Institute, National Environment Agency, Singapore
Borame Sue Dickens	Saw Swee Hock School of Public Health, National University of Singapore, Singapore
Dengue Expert Advisory Panel	
Duane J. Gubler	Duke-NUS Medical School, National University of Singapore, Singapore
Ary Hoffmann	Monash University, Australia
Christl Donnelly	Imperial College London, United Kingdom
Chia Kee Seng	Saw Swee Hock School of Public Health, National University of Singapore, Singapore
Neil Ferguson	Imperial College London, United Kingdom
Tim Barkham	National Centre for Infectious Diseases, Singapore
Vernon J Lee	Ministry of Health, Singapore

Trial Sponsor: Environmental Health Institute, National Environment Agency, Singapore

Synopsis

Primary registry and trial identification number	This protocol was registered on ClinicalTrials.gov as NCT05505682
Date of registration in primary registry	August 18 2022
Financial support	Singapore Ministry of Finance, Ministry of Sustainability, and the National Environment Agency, and the Singapore National Robotics Program.
Sponsor	Environmental Health Institute, National Environment Agency, Singapore
Title	Assessing the efficacy of male Wolbachia-infected mosquito deployments to reduce dengue incidence in Singapore
Study setting	Singapore, Singapore
Health condition(s) study	Dengue
Intervention	Intervention arm: Deployment of male Wolbachia-infected Aedes aegypti mosquitoes, in addition to standard practice dengue control activities.<br><br>Comparison arm: Standard practice dengue control activities.
Primary endpoints	1. Symptomatic, virologically-confirmed dengue virus (DENV) infection of any severity.<br><br>2. Township-level dengue incidence rate
Secondary endpoints	1. Prevalence of *Ae. aegypti/Ae. albopictus* mosquitoes<br><br>2. Public Attitudes, Perceptions & Knowledge (APK) of *Wolbachia* and other vector control interventions
Study design	Study type: intervention study with test-negative design<br>Allocation: cluster randomised<br>Assignment: parallel, 8 intervention, 7 control arms<br>Masking: non-blinded<br>Primary purpose: prevention
Study duration	24 months, with possibility of extension of up to 3 years
Target sample size	First primary endpoint: 1600 test-negative controls and 400 dengue-positive cases will be needed to detect a 50% reduction in dengue incidence with 80% power.<br><br>Second primary endpoint: At least 31 months would be required post-intervention to detect a 50% reduction in dengue incidence rates at 80% power.
Analysis	Logistic regression model will be used to estimate the odds ratio of dengue in Wolbachia treated versus untreated clusters.<br><br>The intention-to-treat analysis will consider *Wolbachia* exposure as binary depending on the allocation of the cluster of residence.

Updates

Update date: 14 June 2025

**Section**	Details
Data management and governance	Addition on details of trial steering committee members

Update date: 1 March 2024

**Section**	Details
Data	Supplemental environmental and anthropogenic data to control for baseline dengue risk in intervention and control arms
Statistical analysis: primary endpoints	Addition of additional steps required to correct for potential imbalance in environmental and anthropogenic characteristics between arms
Statistical analysis: secondary endpoints	Addition of additional steps required to correct for potential imbalance in environmental and anthropogenic characteristics between arms
Selection of patients	Addition of exposure criteria based on number of months a tested individual was exposed to *Wolbachia-Aedes* interventions

Background

In recent years, outbreaks of arboviruses transmitted by *Aedes* mosquitoes, such as dengue, Zika, and yellow fever, have become increasingly common around the world[1]. Key factors for the rise in incidence include increased urbanization, globalization, and long-distance travel, which encourage the spread and endemicity of the viruses and their vectors, particularly the peri-domestic *Aedes aegypti* species[1,2] At present, dengue is the most widespread arboviral disease worldwide and is endemic throughout the tropical belt[3,4]. An estimated 105 million dengue infections occur annually with associated costs of around USD8.9 billion[3,5]. Other arboviruses that have dominated headlines include Zika and chikungunya: Zika caused a global epidemic in 2016 with more than 1.5 million infections in over 70 countries[6,7], with transmission still recorded by more than 80 countries in 2019 alone[7]. Meanwhile, chikungunya outbreaks have occurred frequently since the first major outbreak in 2004, which spread from Kenya to Reunion Island, followed by South and Southeast Asia by 2008, and are estimated to have caused an average yearly loss of over 106,000 DALYs globally from 2010 to 2019[8,9].

Current treatments for arboviral infections attempt to alleviate the symptoms of infection but do not address the root cause[10]. With the exception of yellow fever and, more recently, dengue, no suitable vaccine is available for arboviruses borne by *Ae. aegypti*[11]. However, vaccination rates for yellow fever are decreasing[12], and the sole licensed dengue vaccine at present, Sanofi-Pasteur's CYD-TDV, also known as Dengvaxia®, is plagued with safety concerns among immunologically naïve individuals[13]. As such, vector control remains the primary tool for mitigating the spread of arboviruses. Conventional vector control measures include source reduction[14], space spray, and targeted indoor residual spraying with insecticides to kill adult mosquitoes and chemical treatment of containers to kill larvae[15]. These are complemented with enforcement and legislation such as regular home inspections for potential breeding sites, educational campaigns, and community engagement for better environmental management[14,16]. A major drawback of conventional vector control efforts is the need for thorough and sustained implementation in order to successfully bring down the vector populations[15]. Moreover, vector resistance to insecticides may arise with prolonged use[17]. A recent meta-analysis of conventional vector control measures against dengue vectors showed that their efficacy is limited in some cases[18]. Therefore, there is a pertinent need for novel vector control strategies to mitigate the spread of arboviral diseases.

One novel approach is the incompatible insect technique (IIT), which involves the release of male mosquitoes infected with the maternally inherited intracellular bacterium *Wolbachia*[19–21]. Mating between infected males and uninfected females results in non-viable offspring through cytoplasmic incompatibility (CI), leading to suppression of the mosquito population[20,21]. *Wolbachia*-mediated IIT as a method of vector control has been tested in China, the USA, Thailand, and Singapore, the country of focus for this study protocol[22–25]. Singapore, among other programs that also attempt to control the *Ae. aegypti* population, has coupled IIT with sterile insect technique (SIT), using irradiation to sterilize residual females (due to imperfect sex sorting) before releases of *Wolbachia*-infected males. IIT-SIT reduces the likelihood of establishment of the released *Wolbachia* strain in field mosquito populations, which would render CI ineffective.

Singapore is an equatorial city-state in Southeast Asia where *Ae. aegypti* is ubiquitous and dengue is hyperendemic[26,27]. Besides dengue, the country has also reported sporadic chikungunya infections since 2013, following an outbreak in 2008 and 2013[28,29] and experienced a Zika outbreak in 2016[30,31]. Decades of conventional vector control efforts have contributed to falling dengue seroprevalence rates which has rendered the population vulnerable to dengue outbreaks despite a low *Ae. aegypti* population[32,33]. The most recent outbreak in 2020 was the largest on record with an all-time high of 1792 weekly reported cases[34], which was partially attributable to non-pharmaceutical interventions motivated by the concurrent SARS-CoV-2 outbreak[35–37].

Singapore has since 2016 been conducting phased field trials to evaluate the use of IIT-SIT to suppress the local *Ae. aegypti* population. IIT-SIT was chosen over an alternative *Wolbachia* dengue control strategy involving introgression of the bacterium into field mosquito populations to reduce their ability to transmit dengue. This latter approach, which requires release of both male and female *Wolbachia*-infected mosquitoes, has shown promise in trials in Australia and Indonesia[38,39]. However, IIT-SIT harmonizes with Singapore's decades-long vector control program which focuses on suppressing the local mosquito population. It received greater social acceptance since it does not release biting female mosquitoes and has negligible ecological impact[40]. Additionally, IIT-SIT is not subject to the risk of dengue mutants escaping the inhibitory effect of *Wolbachia*[41]. The trials have thus far demonstrated reductions of *Ae. aegypti* populations and dengue incidences by 98% and 88%, respectively[24,42]. Further analyses using quasi-experimental methods including 2 additional field trial sites and observational duration demonstrated that the intervention can lead to reductions of up to 78% and 99% respectively for dengue and *Ae. aegypti* populations respectively[43–45]. A modeling study has promisingly found that a *Wolbachia*-mediated IIT strategy that was conservatively 40% efficacious would have retrospectively averted an estimated USD330 million in economic costs over the ten-year period between 2010 and 2020[46].

Building on the success of the phased field studies, the efficacy of IIT-SIT in reducing dengue incidence will next be tested in a national level cluster-randomized (CR) controlled trial by adapting the methods of Anders et al.,2018[47]. Here, we present the protocol of the planned study which, to our knowledge, would be the first CR controlled trial to experimentally measure the efficacy of IIT-SIT in reducing dengue transmission.

<u>Key study characteristics</u>

Research Question Does large-scale deployment of male *Wolbachia*-infected *Ae. aegypti* (*Wolbachia-Aedes*) mosquitoes lead to a significant reduction in dengue incidence in people living in release areas, compared to control areas without release?

Rationale *Wolbachia-Aedes* deployments mosquitoes have previously been demonstrated to suppress field *Aedes aegypti* and *albopictus* populations[24,43–45]. Previous field studies have also retrospectively demonstrated the protective effect of *Wolbachia-Aedes* deployments on dengue**.**

Th aim of the study outlined in this protocol, is to experimentally ascertain the efficacy of large-scale *Wolbachia-Aedes* deployments in reducing dengue virus transmission in field conditions. Cluster randomised trials (cRCT) represent the gold standard design to provide evidence on the efficacy of a field-based intervention. The value of estimating the epidemiological impact of *Wolbachia* using a cRCT approach is as follows: (**1**) RCTs are the gold standard and preferred method for estimating the benefit/harm of public health interventions. (**2**) RCTs have highest weight in shaping policy and practice in public health compared to observational studies and this is likely to be true for the *Wolbachia-Aedes* technology.

Overview The aim of this study is to determine whether large-scale deployment of *Wolbachia*-infected male *Ae. aegypti* mosquitoes (the intervention, thereafter referred to as male *Wolbachia-Aedes*) can reduce the incidence of dengue in individuals living in intervention (treatment) clusters, compared to individuals living in non-intervention clusters. The two primary endpoints are (1) odds ratio of *Wolbachia* exposure distribution (i.e., probability of living in an intervention cluster) among laboratory-confirmed reported dengue cases compared to test-negative controls, and (2) laboratory-confirmed reported dengue case counts normalized by population size in intervention versus non-intervention clusters. Secondary endpoints include the efficacy of male *Wolbachia-Aedes* deployment in reducing *Ae. aegypti* mosquito populations, the impact of male *Wolbachia-Aedes* deployment on *Ae. albopictus* mosquito populations, and the pre-post trial community attitudes/knowledge and acceptance of male *Wolbachia-Aedes* and other vector control practices. Exploratory endpoints include the impact of male *Wolbachia-Aedes* deployment on the circulation of dengue cases of specific serotypes and on secondary infections.

Study Objectives

Primary objectives:

- To assess the efficacy of large scale *Wolbachia-Aedes* deployments in reducing the risk of symptomatic, virologically-confirmed dengue cases of any severity in Singapore in release areas, relative to non-release areas.
- To assess the efficacy of large scale *Wolbachia-Aedes* deployments in reducing the incidence of symptomatic, virologically-confirmed dengue cases of any severity in Singapore in release areas, relative to non-release areas.

Secondary objectives:

- To assess the efficacy of large scale *Wolbachia-Aedes* deployments in reducing the abundance of *Aedes aegypti* in Singapore in release areas, relative to non-release areas.
- To assess the effect of large scale *Wolbachia-Aedes* deployments on the abundance of *Aedes albopictus* in Singapore in release areas, relative to non-release areas.
- To assess the changes in Public Attitudes, Perceptions & Knowledge (APK) of *Wolbachia* and other vector control interventions in release and non-release areas.

Study design

Type of study This is a parallel two-arm non-blinded cluster randomised controlled trial which will be conducted in a single site in Singapore, an equatorial city-state. The study site will be subdivided into 17 non-contiguous clusters, approximately 0.79km^2 in size (range 0.53km^2-1.33km^2) and 114 high-rise public apartment blocks (range 70–173). Clusters will be randomly allocated to receive *Wolbachia-Aedes* deployments or no intervention, such that 8 clusters receive *Wolbachia* deployments and 7 receive no intervention.

Where possible, manmade or natural borders such as major roads, highways, and water bodies will be used to delineate cluster boundaries to limit spillover of male *Wolbachia-Aedes* from intervention to non-intervention clusters, as well as migration of wildtype mosquitoes into clusters. In the absence of such borders, adjacent areas within a 300m radius were designated as bufer release areas, should the cluster be designated for intervention; clusters (inclusive of buffer areas) were kept at least 700 m apart. Conventional vector control activities by public health agencies, such as media engagement, home inspections, breeding site destruction, and space spray in response to dengue cluster, will continue as per routine practice across the study area and duration in both intervention and non-intervention clusters.

The impact of *Wolbachia-Aedes* deployments on dengue risk will be assessed by comparing the exposure distribution (probability of living in a *Wolbachia-Aedes* treated area) among test-positive dengue cases presenting to any public hospital, polyclinic or general practitioner for febrile illness, against the exposure distribution among patients with febrile illness presenting to the same network of public healthcare institution. Dengue test-positives and test-negatives will be sampled concurrently from the population of patients presenting with febrile illness to the primary healthcare network, with case or control status retrospectively identified based on the results of laboratory diagnostic testing.

The distribution of *Wolbachia* exposure in the sampled test-negative controls will reflect the distribution of *Wolbachia* exposure in the underlying source population that gave rise to test-positive cases. This will be met, so long that the core that the relative propensity to seek healthcare for febrile illness at a primary healthcare insitution in intervention versus untreated arms is the same for dengue cases as other febrile illness test-negative controls. This should be upheld if dengue cases and other febrile illness controls are clinically indistinguishable until laboratory diagnosis. The concurrent sampling of test-positives and negatives mean that the odds of *Wolbachia-Aedes* exposure among sampled dengue test-positive cases relative to test-negative controls (odds ratio), is an unbiased estimate of the relative incidence of dengue in *Wolbachia*-intervened versus non-intervened clusters, and can thereby be used to directly estimate the protective efficacy of *Wolbachia-Aedes*. The null hypothesis is that the relative incidence of virologically-confirmed dengue in *Wolbachia-Aedes* treated and untreated areas is one (i.e the computed odds ratio equals 1). If *Wolbachia-Aedes* has a protective effect against dengue, the incidence rate ratio for virologically-confirmed dengue in *Wolbachia*-treated areas versus untreated areas will be less than one.

Justification of study design cRCTs are the gold standard study design to provide evidence on the efficacy of a field-based intervention, and involves allocating the intervention or control to clusters. In this case, *Wolbachia-Aedes* deployments are randomly allocated to intervention clusters spatially and control clusters receive no intervention. The traditional implementation of cRCT under an endpoint of infection/disease incidence would be measured using a prospective cohort of individuals residing in treated/untreated clusters, and the measure of effect would be a risk or rate ratio in treated/untreated arms. Given the historically low force-of-infection for dengue and number of cases due to successful vector-control in the study setting[32], an untenably large cohort of individuals followed for a long duration would be required to detect the requisite number of dengue infections for sufficient power.

Therefore, we employed an alternative, cluster-randomized test-negative design to measure the efficacy of *Wolbachia-Aedes* deployments, to concurrently sample test-positive dengue cases and test-negative controls reporting from other febrile illness from the study population to derive the estimate of the incidence rate ratio. By leveraging on nationally representative dengue surveillance and testing databases comprising all public hospitals, polyclinics and general practitioners, we could obtain information on individuals whom present to these insitutions with febrile illness, with dengue test-positives classified as cases and test-negatives classified as controls. Here, the controls are drawn from the same source population as the cases, thus avoiding the common limitation of control selection in traditional case-control studies that can introduce selection bias[48]. Thereafter, computation of the odds ratio would provide an unbiased estimate of the rate ratio in the source population over the period of participant enrolment, without requiring any rare disease assumptions[49,50]. This test-negative design has the advantage of being more efficient, cost effective, and logistically simpler to achieve than a large prospective cohort, and can be conducted easily using observational information from nationally-representative testing databases.

Number of participants The study area comprises of approximately 722,204 residents in 15 non-contiguous clusters comprising high-rise public apartment blocks. It is estimated that 1,600 test-negative controls and 400 dengue-positive cases will be needed to detect a 50% reduction in dengue incidence with 80% power. Sample size requirements will be re-estimated using observed data after attaining 50% of the target data collection. We follow[47,51,52] to estimate the sample size for the proposed study design. We used the comparison of exposure odds among test-positive cases vs. test-negative controls for data aggregated across all clusters, with the null hypothesis that the odds of residence in a treatment cluster is the same among test-positive cases as test-negative controls[47,51,52]. This provides an unbiased estimate of the relative risk between groups as demonstrated previously[47,51,52]. We performed simulations (n = 1,000) to estimate the power to detect a 50% intervention efect, assuming eight clusters per arm, and using available historical dengue testing data in the 15 study clusters. We randomly allocated eight clusters to receive the intervention; this random allocation was repeated 10,000 times, and allocations were only kept when constrained randomization criterion were met (n = 3,151 possible balanced allocations). In each iteration of the simulation, we randomly selected one distinct intervention allocation and a 2-year time frame from the historical dengue testing data and simulated a 50% intervention efect in the intervention arm in the latter 12 months. The simulated data was analyzed using logistic regression to assess the signifcance of intervention efect. These were repeated for various dengue test sample sizes. We computed the proportion of signifcant results obtained in each scenario. This provided an estimate for the power of our study and the sample size required to obtain the respective power[47].

Expected study duration Deployment of male Wolbachia-Aedes and data collection from diagnostic laboratories is expected to commence in July 2022 (see Table 1). Data collection will continue for least 24 months or longer if needed to reach the minimum sample size required for intention-to-treat analysis. To account for year-to-year variability in dengue cases, data collection will continue for at least 24 months even if the estimated minimum sample size is attained sooner.

Key Activities	Period
Randomization	Feb 2022
Engage healthcare institutions for data collection	March 2022–June 2022
Community Engagement	June 2022 onwards
Start of RCT	July 2022
Midterm review	July 2023 onwards
End of RCT	September 2024
Final Analysis	September 2024–December 2024
Publication of results	December 2024

Table 1: Trial timeline

Primary outcomes: dengue The primary outcome measure will be virologically confirmed dengue virus infection in patients reporting febrile illness. All dengue-suspected patients will be classified as dengue cases using an internally controlled RTqPCR assay, dengue non-structural protein 1 (NS1) or IgM as diagnostic assays to detect dengue virus[47,53,54]. Dengue cases are patients with virologically confrmed DENV infection through RTqPCR, testing positive for NS1 antigen or IgM, according to Ministry of Health, Singapore (MOH) criteria. A positive test for any of the three assays would classify the patient as a dengue case. Controls are patients with negative test results for DENV through RT-qPCR, NS1 antigen ELISA, or DENV IgM.

Secondary outcome: *Aedes aegypti* and *albopictus* The secondary outcome measure will be *Aedes aegypti* and *Aedes albopictus* abundance. This will be measured using the Gravitrap index. Gravitraps are simple, hay infusion-filled cylindrical traps with a sticky lining on the inner surface designed to lure and trap gravid female *Aedes* developed by the National Environment Agency, Singapore[55]. Adult *Aedes* populations in intervention and non-intervention clusters will be monitored weekly using an average of 6 to 9 Gravitraps per high-rise apartment block[24]. Details on entomological monitoring are provided below.

Study setting The study will be conducted in high-rise public housing areas in Singapore. Singapore has an area of 728 km^2 and a population of approximately 5.7 million as of 2020[56]. The study sites cover 10.9 km^2 and have an estimated total population of 722,204, with an average population density of approximately 66,257 persons per km^2 .

Annually, the number of reported dengue cases in Singapore ranged between 51 and 621 cases per 100,000 individuals from 2010 to 2020[56,57]. Fifteen clusters were identifed as locations at high risk of dengue transmission, each with an average area of 0.79 km2 (range 0.53–1.33 km2) and 114 high-rise public apartment blocks[58] (range 70–173). 8 clusters will be randomly allocated intervention and 7 clusters will be randomly allocated as control sites which do not receive intervention.

Study intervention

Randomisation method Due to the small number of clusters available for randomization, selection of clusters will rely on constrained randomization to prevent chance imbalances in baseline historical dengue risk between intervention and non-intervention arms. The proportion of positive to negative dengue samples over 2016 to 2019 will be used as the constraining variable. We will generate a large number (*n* = 10,000) of potential random cluster allocations in 8:7 intervention/non-intervention ratio. For each allocation, the value of the constraining variable will be calculated in each study arm using the aggregate arm-level value. Each potential random allocation will be evaluated against the pre-defined balancing criteria (i.e., no statistically significant difference in the proportion of positive to negative dengue samples between the two arms) and removed as a potential random allocation if they were not met. All potential allocations that satisfy the balancing criteria were kept (*n* = 3151, exceeding the threshold of 100–150 allocations recommended in the literature), and a single allocation will be randomly selected from within the restricted list of balanced allocations. Finally, a single random draw was used to determine which of the two study arms was to receive male *Wolbachia-Aedes* releases. Randomization will be followed by extensive community engagement to seek the support of local politicians and grassroot leaders of each of the eight intervention clusters.

***Wolbachia-Aedes* deployment** Low-dose (~40 Gy or lower) X-ray-irradiated male *w*AlbB-infected *Ae. aegypti* (male *Wolbachia-Aedes*) will be released in designated public locations in high-rise housing estates in the intervention clusters[24]. Releases may commence across all intervention clusters simultaneously or, if operationally more feasible, be staggered between three lots across two months apart. Releases will be conducted twice a week, during periods of highest *Ae. aegypti* activity (weekdays between 0630–1100h and 1300–1800h)[24]. To facilitate even distribution of mosquitoes, releases will be conducted in equally spaced release locations per apartment block, on the ground, middle (levels 5–8) and high floors (≥ level 9). Based on previous studies[24], we expect to release 1–6 male mosquitoes per resident, with the release numbers adaptively guided by the Gravitrap index at each location each release week. Mosquito monitoring will continue throughout the study period. Male *Wolbachia-Aedes* will also be released twice a week on the ground floors of high- and low- rise housing estates within the designated buffer areas[59]. Where releases in buffer areas cannot be performed (e.g., schools), releases will be conducted at the boundary between such areas and the intervention sites. This is to counteract immigration of wild-type *Ae. aegypti* females from contiguous non-release areas.

***Wolbachia-Aedes* production** Eggs will be submerged in hatching broth (0.071g baker's yeast (MP Biomedicals, France), 0.357g nutrient media (CM0001 Nutrient broth, Oxoid, England) or 0.536g nutrient media (Liofilchem, Italy) and 1000ml reverse osmosis (RO) water) to induce hatching. After two hours in hatching broth aged overnight, larvae will be counted using an automated larvae counter (Orinno Technology Pte Ltd, Singapore) and transferred to rearing trays measuring 103 x 63 x 3 cm at a density of 4.7 larvae per ml of RO water, respectively. Larvae will be fed with ground ALTech Arthropod Larvae Feed (ALTech, Singapore) in slurry form. Six days post-hatching, male pupae will be separated from female pupae and larvae using the Pupae Separation System (Orinno Technology Pte Ltd, Singapore) prior irradiation for IIT-SIT releases. Pupae will be allowed to eclose inside release containers.

Irradiation will be performed with RS2400V irradiators (Rad Source Technologies Inc., USA). For IIT-SIT irradiation (female sterilization), pupae will be irradiated in plastic containers measuring 6.5 cm in diameter and 7.0 cm in height (for release). For SIT irradiation (male sterilization), pupae will be irradiated in petri dishes with diameter 9.4 cm. Petri dishes or containers will be placed at the center of the irradiation chamber of the X-ray irradiator. The effective dose will be 30 Gy to 40 Gy, depending on pupae density, irradiator model and desired sterility.

Entomological monitoring strategy Gravitraps are simple, hay infusion-filled cylindrical traps with a sticky lining on the inner surface designed to lure and trap gravid female *Aedes* developed by the National Environment Agency, Singapore[55]. Adult *Aedes* populations in intervention and non-intervention clusters will be monitored weekly using an average of 6 to 9 Gravitraps per high-rise apartment block[24]. Gravitraps are placed in public spaces along corridors of public housing apartments in all sites, at the ratio of 1 trap for every 20 households. This trap-to-household ratio and deployment was based on logistic considerations for long term monitoring, and it provides an assessment of the density as well as vertical distribution of the mosquito population. Mosquito data is collected from all Gravitraps on a bi-weekly basis, and mosquito samples were sexed and morphologically identified. Female *Aedes* mosquitoes that cannot be speciated morphologically will be identified using a multiplex *Ae. aegypti*- and *Ae. albopictus*-specific real-time qPCR assay targeting the COI gene (*Aedes* forward 5'-TCC CGC CTT CRG TGC GCG G-3', *Aedes* reverse 5'-CGC GGG ATG TAY TCA TCA ACC-3', probe AEG Cy5 – 5'-TAG TCA GAC GTG GTG GTG ACA CAC C-3'– BHQ2, and ALBO HEX – 5'-ACG GTG GCC GGC GTG CCA GTC GT-3' – BHQ1). The multiplex qPCR reactions were performed in 20µl total volume containing 1X SensiFAST No-Rox Probe Mastermix (Bioline, USA); 0.15µM and 0.05µM COI primers and probes, respectively; 0.4 µM and 0.15µM wsp primers and probes, respectively; and 2µl. Cycling will be carried out in a Rotorgene Q (Qiagen, Germany) using the following conditions: 95°C for 5 min, 40 cycles at 95°C for 10s, and 60°C for 30s.

Furthermore, screening for the presence of *Wolbachia* in trapped female *Aedes aegypti* may be carried out to monitor for *Wolbachia* establishment. While irradiation greatly reduces the risk of establishment, this is a precautionary measure for early detection in the unlikely event that a sufficient number of fertile females are released to result in establishment. If *Wolbachia* becomes established in an intervention cluster, male *Wolbachia-Aedes* irradiated for male sterility at a higher X-ray dose (≥ 40 Gy) or male *Aedes aegypti* infected with another incompatible *Wolbachia* strain may be released to mitigate the establishment, which is expected to hamper mosquito population suppression.

Selection of patients

Selection of patients No enrolment of patients will conducted. The trial will rely on collection of data from dengue-suspected patients, whose blood samples are sent for dengue tests, to all diagnostic laboratories that support private clinics, public polyclinics, or public/private hospitals. This network is nationally representative and comprises tests of all individuals reporting for testing in public/private hospitals and public polyclinics. Dengue-suspected patients are identified by clinicians through symptoms such as high fever, body aches, and rashes, coupled with absence of respiratory symptoms. Cases and test-negative controls will be retrospectively classified through laboratory test results. In addition, we will also obtain spatially resolved counts of laboratory-confirmed dengue cases reported to the Ministry of Health. Approval from the Director of Medical Services has been obtained to collect data for the purpose of the trial.

The original protocol did not explicitly define the inclusion criteria of residents based on the duration of *Wolbachia* exposure at the point of dengue testing. Preliminary field trial data from sites prior to the commencement of the cRCT demonstrated that at least 3–12 months was required for the intervention to demonstrate noticeable suppression in *Aedes aegypti* abundance For the first primary endpoint we aimed to examine the intervention efficacy of *Wolbachia-Aedes* interventions to reduce the risk of contracting dengue using a test-negative design. To understand any potential relationship between the duration of intervention and the risk of contracting dengue at the point of testing, we will consider participants to be exposed/unexposed to *Wolbachia-Aedes* in the study based on whether they were subject to 0, 3 or 6, or more months of intervention or residing in a control site respectively at the time of testing in the post-intervention period. Higher exposure durations will be considered if sample size is sufficient for analyses. Exposed and unexposed participants from the same exposure duration will be compared. Similarly, for the second primary endpoint of examining the efficacy of *Wolbachia-Aedes* to reduce town-level dengue case counts normalized by cluster population size, we will re-aggregate intervention efficacies based on 0, 3 or 6 or more months of intervention for intervention sites to compare the reduction in dengue incidence versus controls in the post-intervention period. A further re-aggregation based on calendar time would also be explored following previous work in the study setting showing that intervention efficacies may be confounded by whether it was a national inter-epidemic or epidemic year, and to remove temporal confounding effects from analyses. For the secondary endpoint of examining the efficacy of Wolbachia to reduce town-level *Aedes aegypti* abundance, we will re-aggregate intervention efficacies based on 0, 3 or 6 or more months of intervention for intervention sites to compare the reduction in Aedes abundance versus controls in the post-intervention period.

Inclusion criteria All patients who report to testing in public/private hospitals and public polyclinics who have dengue-suspected illness.

Exclusion criteria If test data from more than one episode of febrile illness is reported for a single individual, the data will only be included if the test episodes are more than 4 weeks apart.

Data collection procedures

For epidemiologic monitoring Identifiable individual-level data on laboratory-confirmed dengue cases and dengue test-negative controls (including residential postal codes, basic socio-demographic information, and details about dengue test status) will be obtained from the national surveillance network of diagnostic laboratories which receive samples for dengue testing from private clinics, public polyclinics, and public/private hospitals. Individual-level data will first be cleaned up and checked for duplicates by selected analysts approved to access and handle identifiable data. If test data from more than one episode of febrile illness is reported for a single individual, the data will only be included if the episodes are more than 4 weeks apart. Data will then be de-identified before further analysis. In addition, we will also obtain spatially resolved counts of laboratory-confirmed dengue cases reported to the Ministry of Health. Regular data monitoring will be conducted to identify biases in response and/or missing data.

For entomologic monitoring, Gravitraps will be used. Gravitraps are simple, hay infusion-filled cylindrical traps with a sticky lining on the inner surface designed to lure and trap gravid female *Aedes* developed by the National Environment Agency, Singapore[55]. Adult *Aedes* populations in intervention and non-intervention clusters will be monitored weekly using an average of 6 to 9 Gravitraps per high-rise apartment block[24]. Gravitraps are placed in public spaces along corridors of public housing apartments in all sites, at the ratio of 1 trap for every 20 households. This trap-to-household ratio and deployment was based on logistic considerations for long term monitoring, and it provides an assessment of the density as well as vertical distribution of the mosquito population. Mosquito data is collected from all Gravitraps on a bi-weekly basis, and mosquito samples were sexed and morphologically identified. Female *Aedes* mosquitoes that cannot be speciated morphologically will be identified using a multiplex *Ae. aegypti*- and *Ae. albopictus*-specific real-time qPCR assay targeting the COI gene (*Aedes* forward 5'-TCC CGC CTT CRG TGC GCG G-3', *Aedes* reverse 5'-CGC GGG ATG TAY TCA TCA ACC-3', probe AEG Cy5 – 5'-TAG TCA GAC GTG GTG GTG ACA CAC C-3'– BHQ2, and ALBO HEX – 5'-ACG GTG GCC GGC GTG CCA GTC GT-3' – BHQ1). The multiplex qPCR reactions were performed in 20µl total volume containing 1X SensiFAST No-Rox Probe Mastermix (Bioline, USA); 0.15µM and 0.05µM COI primers and probes, respectively; 0.4 µM and 0.15µM wsp primers and probes, respectively; and 2µl. Cycling was carried out in a Rotorgene Q (Qiagen, Germany) using the following conditions: 95°C for 5 min, 40 cycles at 95°C for 10s, and 60°C for 30s.

Supplemental environmental and anthropogenic data to control for baseline dengue risk in intervention and control arms We additionally extracted a set of spatially and temporally explicit variables to represent environmental heterogeneity across the study sites which was previously not available to the study team. The data would be used to explore and adjust for balances in variables which may potential confound historical dengue risk, using propensity scoring models as described in revised statistical analysis below. Several of these factors were previously found to be associated to Aegypti abundance and dengue transmission from previous baseline studies in the same study setting[60–62] . (1) A 10m vegetation map[63] with areas classified across multiple vegetation types including grass, forest and managed vegetation based on Sentinel-2 satellite data, was utilised to signify the availability of natural breeding sites and nectar availability for mosquito males. The percentage cover of each vegetation type was calculated within each sector as mosquitoes often show preferential areas to breed and rest. Similarly, the averaged Landsat Normalized Difference Vegetation Index per sector was also utilised for this purpose[64]. (2) To represent both host density and urban breeding habitat availability, data on the locations of Housing and Development Board (HDB) public housing estates where over 80% of Singapore's resident population reside was obtained from Onemap[65]. Utilising the HDB location and HDB resale data, the average age of HDB buildings was collected as older age is a well-established risk factor for higher Gravitrap indices[60,62] . This is due to building deterioration providing additional breeding habitats in cracks and design features such as laundry poles which are no longer built due to the pooling of water within the supports. Average

HDB house price from 2015–2022, a proxy for household income and socioeconomic status, was calculated based on an XGBoost model previously employed[66]. Building height, which has also been correlated to Gravitrap indices, was calculated according to the number of floors and average height of each level of 3m. The number of condominiums and landed properties was additionally collected within each sector representing additional hosts being available. The percentage cover of built area was calculated as a sum of all residential, commercial and industrial buildings, representing the level of urbanicity, which has been associated with *Ae. aegypti* presence[67]. The major open drainage network for Singapore was obtained from the Public Utilities Board as a key breeding site for mosquitoes around HDBs. The average distance of each HDB block within a sector to a drain was measured as well as the length of the network within the sector6 .

(3) For meteorological data, well-established variables which are known to affect mosquito survival or fecundity were collected. These included daily mean, maximum, and minimum temperature, total rainfall, maximum rainfall falling within a 30-minute, 60-minute and 120-minute window, and wind speed, which were obtained from a total of 21 weather stations installed by the National Environment Agency. We created daily complete raster maps through inverse distance weighting interpolation, which was carried out using cross validation of leave-one-out for the fitting of the inverse distancing power to minimise the error in observation on the raster surface of the test point. Hourly dewpoint and ambient ground air temperature were taken from ERA5, published by ECMWF[68], to estimate relative humidity over the time period using standard formula. These values were aggregated at a weekly level to correspond with the dengue case data.

Data management and governance Access to trial data will require a restricted VPN connection and a data access key only shared with specific individuals authorized by senior management and the trial Principal Investigator. In addition, data can only be accessed by study personnel who have completed all required cyber/data-security training. All patient data, including dengue test status and residential postal code, clinical, and sociodemographic information will be kept strictly confidential. Periodic reviews by data monitors (internal to the National Environment Agency but who are not involved in the trial) will be conducted to ensure adherence to data governance measures.

Trial data and study information will only be released under prior written approval of the Trial Steering Committee. Patient data will not be released without written permission from respective patients, except for monitoring by ethical review board or regulatory agencies in de-identified form. Reporting of study results will not permit identification or the place of residence of individual patients.

Members of Trial Steering Committee	
Lee Ching Ng (co-chair)	Environmental Health Institute, National Environment Agency, Singapore
Vernon J Lee (co-chair)	Ministry of Helath, Singapore
Shuzhen Sim	Environmental Health Institute, National Environment Agency, Singapore
Tan Cheong Huat	Environmental Health Institute, National Environment Agency, Singapore
Judith Chui Ching Wong	Environmental Health Institute, National Environment Agency, Singapore
Low Wai Leong	Environmental Health Institute, National Environment Agency, Singapore

Statistical methods

Power calculations—first primary endpoint It is estimated that 1,600 test-negative controls and 400 dengue-positive cases will be needed to detect a 50% reduction in dengue incidence with 80% power. Sample size requirements will be re-estimated using observed data after attaining 50% of the target data collection. We follow[47,51,52] to estimate the sample size for the proposed study design. We used the comparison of exposure odds among test-positive cases vs. test-negative controls for data aggregated across all clusters, with the null hypothesis that the odds of residence in a treatment cluster is the same among test-positive cases as test-negative controls[47,51,52]. This provides an unbiased estimate of the relative risk between groups as demonstrated previously[47,51,52].

We performed simulations (n = 1,000) to estimate the power to detect a 50% intervention effect, assuming eight/seven clusters per arm, and using available historical dengue testing data in the 15 study clusters. We randomly allocated eight clusters to receive the intervention; this random allocation was repeated 10,000 times, and allocations were only kept when constrained randomization criterion were met (n = 3,151 possible balanced allocations). In each iteration of the simulation, we randomly selected one distinct intervention allocation and a 2-year time frame from the historical dengue testing data and simulated a 50% intervention effect in the intervention arm in the latter 12 months. The simulated data was analyzed using logistic regression to assess the significance of intervention effect. These were repeated for various dengue test sample sizes. We computed the proportion of significant results obtained in each scenario. This provided an estimate for the power of our study and the sample size required to obtain the respective power[47].

Power calculations—second primary endpoint Intervention efficacy in the second primary endpoint from the difference-in-differences strategy as described below, laboratory-confirmed dengue case counts normalized by cluster population size (incidence rate), which is aggregated at the cluster level and measured across months and across clusters. We followed Indriani et al., 2020[69] to estimate the required post-intervention duration required to detect a 50% reduction in dengue incidence rates with 80% power.

The power calculation was conducted by performing simulations (n = 1,000) to estimate the power to detect a 50% intervention effect, assuming eight and seven clusters in the intervention and non-intervention arm respectively, and using historical dengue incidence rate data (2015–2021) in the 15 study clusters. These clusters were constrained randomized to obtain balanced intervention/non-intervention arms as delineated in the preceding sections. In each iteration of the simulation, we randomly selected one distinct intervention allocation and 0.5, 1, 1.5, 2, 2.5, and 3 year time frames post-intervention, with pre-intervention period fixed at 4 years and simulated a 50% intervention effect in the intervention arm in the following intervention. The simulated data was analyzed using the difference-in-differences specification (see below) to assess the significance of intervention effect. We then computed the proportion of significant results obtained in each scenario. This provided an estimate for the power of our study and the measurement duration required to obtain the respective power. We estimate that at least 31 months would be required post-intervention to detect a 50% reduction in dengue incidence rates at 80% power.

Pre-post trial attitudes, acceptance, and knowledge survey on *Wolbachia-Aedes* interventions and other vector control practices To assess efficacy of the various engagement strategies employed, an independent consultancy firm will be engaged to conduct door-to-door household perception surveys. These surveys will be used to measure (1) the effectiveness of different community engagement strategies and (2) each strategy's efficacy in shaping community attitudes, perception, and knowledge pre- and post-intervention. Surveyors will be trained before data collection to ensure their understanding of the objectives, methodology, expectations, and questionnaires. The surveys will be conducted in three waves, in households equally selected from the intervention and non-intervention clusters. The first survey will commence three months before and up until the

beginning of *Wolbachia-Aedes* deployments. The second and third surveys will begin 3 months and 1 year following the commencement of *Wolbachia-Aedes* deployments respectively. Each survey wave will be split into three arms, (i) non-intervention clusters with no community engagement, (ii) intervention clusters with standard community engagement practices, and (iii) intervention clusters with alternate community engagement strategies. Households will be selected via stratified systematic sampling. Households will be stratified according to ethnic group denomination and minority socio-demographic factors and systemically sampled. Minority groups will be accounted for in higher proportions to reflect adequate representation of these groups. To accurately reflect the general sentiments of the working and non-working populations, surveys will be administered during the evenings of the working days and weekends. The sampled list of households across the different timepoints will be mutually exclusive to prevent contamination of questions across waves. Survey questionnaires used will be tailored to collect specific information across various timepoints and study arms. For each survey session, the surveyor will approach one respondent from each household to interview, after obtaining informed consent to participate. In the event of (i) resident refuses to participate and (ii) resident is not home, the surveyor will note down these households and arrange for a second visit, before approaching the next household on the list. In the event of a language barrier, surveyors will note the language requirements of the household and a second visit with a surveyor equipped with the necessary language skills will return to the household to conduct the survey. A maximum of one revisit to the same household will be attempted to accurately capture ground sentiments, while remaining prudent with resources.

The National Environment Agency's (NEA) public feedback portal will also be used as an additional data source, to collect residents' feedback submissions related to *Wolbachia-Aedes* deployment in all clusters. Submissions will be classified as positive or negative and tracked through the duration of the study to supplement understanding of residents' attitudes, knowledge, and perceptions of the intervention.

Statistical analysis: primary endpoint—impact of male *Wolbachia-Aedes* deployments on dengue incidence, measured by odds ratio of *Wolbachia* exposure distribution among laboratory-confirmed reported dengue cases compared to test-negative controls In this analysis, male *Wolbachia-Aedes* exposure will be considered as a binary classification based on whether a patient's residence is in an intervention cluster or a non-intervention cluster, where residence is defined as the primary place where the patient resided at reporting date. Logistic regression will be used to assess the intervention effect of male *Wolbachia-Aedes* by estimating the aggregate odds ratio, which compares the exposure odds among test-positive cases versus test-negative controls. The null hypothesis is that the odds of residing in the intervention clusters are the same among test-positive cases as test-negative controls.

A secondary cluster-level analysis will be performed using the cluster-level summary measure of the proportion of dengue test-positive individuals among all individuals tested for dengue. The difference in proportions between the intervention/non-intervention clusters will be used to test the null hypothesis of no intervention effect based on the hypothesis tests described in Jewell et al,.2019[52]. The average proportions between treatment arms can then be used to infer the relative risk (RR) of dengue in intervention versus non-intervention arms following Anders et al.,2018[47].

To account for any residual imbalance in anthropogenic and environmental characteristics, we will explore the utilisation of either inverse-probability weighting, the doubly-robust logistic regression or overlap weighting to account for post-hoc imbalance in aforementioned spatio-temporal characteristics. Secondary cluster-level analysis would also now consider the computation of weighted odds ratios to understand the reduction in odds of being dengue-test positive by residing in a Wolbachia intervention site for a specified amount of time, with inference either being conducted using the permutation inference or cluster bootstrap approach[70]. We will similarly explore utilising either inverse-probability weighting or overlap weighting to account for post-hoc

imbalance in aforementioned spatio-temporal characteristics. Standardised mean differences will be used to compare balance in unweighted and weighted characteristics in the pre-intervention period in intervention and control arms.

Statistical analysis: primary endpoint—impact of male *Wolbachia-Aedes* deployments on dengue incidence, measured by laboratory-confirmed reported dengue case counts normalized by population size in intervention versus non-intervention clusters We will monitor trends in reported laboratory-confirmed dengue case counts in intervention and non-intervention clusters, before, during, and after male *Wolbachia-Aedes* deployments take place. We aim to use a quasi-experimental approach to examine the impact of male *Wolbachia-Aedes* deployment on routine dengue case notifications normalized by cluster population size. Namely, we will use regression models to model the monthly, normalized dengue case counts across sites; we will control for population size and seasonal variability through weather station observations and autocorrelation between sites by inclusion of lag terms. To estimate the impact of male *Wolbachia-Aedes* deployments on routine dengue case counts, the regression specification will include a binary group variable to denote whether a cluster is an intervention/non-intervention cluster, and a binary treatment variable denoted 1 will be included to indicate when interventions occur within that cluster[69]. Following similar difference-in-difference strategies[71], the interaction term between the group and treatment variables will then yield a coefficient, which provides an estimate of the intervention effect of male *Wolbachia-Aedes* deployments on monthly reported case counts. This approach ensures that extraneous factors and selection bias, such as level changes in dengue case counts in either intervention/non-intervention arms post-intervention are removed[71]. Should pre-trends assumptions in dengue case counts between intervention/non-intervention clusters be violated under this quasi-experimental approach, we will instead use synthetic control methods which can more appropriately identify the impact of male *Wolbachia-Aedes* deployments on routine dengue case notifications.

In both primary endpoints, intention-to-treat analysis will classify all individuals/clusters respectively allocated to the intervention arm in the randomization stage as such, notwithstanding any non-treatment of the intervention clusters. In per-protocol analysis, any dropouts of the intervention clusters will be removed from the analysis, and only intervention clusters receiving intervention treatment as well as the allocated controls will be analyzed.

Incorporation of anthropogenic and environmental characteristics will also be explored. Weighted DiD would be explored as an alternative analytical approach if town-level anthropogenic and environmental characteristics were not well-balanced prior to intervention. Weights will potentially be estimated in the pre-intervention period either using inverse-probability weighting or overlap weighting at the town level to balance anthropogenic and environmental characteristics. The weights will then be included in the regression models prior to estimation of intervention efficacies. Standardised mean differences will also be used to compare balance in unweighted and weighted characteristics in the pre-intervention period at the town-level between intervention and control arms.

Statistical analysis: secondary endpoint—impact of male *Wolbachia-Aedes* deployments on the prevalence of *Ae. aegypti/Ae. albopictus* mosquitoes Similarly, we will test whether male *Wolbachia-Aedes* deployments will effectively suppress *Ae. aegypti* mosquito populations as well as whether they have spillover impact on *Ae. albopictus* mosquito populations. Using the nationally representative network of Gravitraps as described above, we will employ a similar quasi-experimental approach to examine the impact of male *Wolbachia-Aedes* deployment on routinely collected *Ae. aegypti/Ae. albopictus* mosquito population data. Namely, we will use regression models with the normal link to model the mean weekly captured *Ae. aegypti/Ae. albopictus* per trap across clusters and control for seasonal variability through weather station observations and autocorrelation between clusters by inclusion of lagged mosquito population terms. To estimate the impact of male *Wolbachia-Aedes* deployments on *Ae. aegypti/Ae. albopictus* mosquito populations, the regression

specification will include a binary group treatment variable to denote whether a site is an intervention/non-intervention cluster, and a binary treatment variable denoted 1 will be included to indicate when interventions occur within that cluster[69]. Following other difference-in-difference strategies[71], the intervention term between the group and treatment variables will then yield a coefficient, which provides an estimate of the intervention effect of *Wolbachia-Aedes* interventions on *Ae. aegypti/Ae. albopictus* mosquito populations weekly. This approach ensures that extraneous factors and selection bias, such as level changes in *Ae. aegypti/Ae. albopictus* mosquito populations in either intervention/non-intervention arms post-intervention are removed. Should pre-trends assumptions in *Ae. aegypti/Ae. albopictus* mosquito populations between intervention/non-intervention clusters be violated under this quasi-experimental approach, we will instead use synthetic control methods which can more appropriately identify the impact of male *Wolbachia-Aedes* deployment on the dependent variable of interest.

Incorporation of anthropogenic and environmental characteristics will also be explored. Weighted DiD would be explored as an alternative analytical approach if town-level anthropogenic and environmental characteristics were not well-balanced prior to intervention. Weights will potentially be estimated in the pre-intervention period either using inverse-probability weighting or overlap weighting at the town level to balance anthropogenic and environmental characteristics. The weights will then be included in the regression models prior to estimation of intervention efficacies. Standardised mean differences will also be used to compare balance in unweighted and weighted characteristics in the pre-intervention period at the town-level between intervention and control arms.

Statistical analysis: secondary endpoint—impact of male *Wolbachia-Aedes* deployments on community attitudes/knowledge and acceptance Using prospectively collected household survey data as described in preceding sections, we will test for differences in engagement and awareness across engagement arms in male *Wolbachia-Aedes* deployment clusters, across 3 sentiment surveys, after which differences and changes in residents' knowledge attitudes and perceptions on male *Wolbachia-Aedes* deployment and vector control practices will be evaluated across intervention and engagement arms. Responses from all survey waves across intervention/non-intervention sites will first be hypothesis tested for differences in the captured socio-demographic characteristics. If there are between-wave differences in socio-demographic characteristics, propensity score matching will be conducted and/or synthetic weights assigned to each observation to mirror the national level socio-demographic composition. To examine the impact of the trial on community endpoints, logistic/multinomial regressions with site- and trial-specific fixed effects will be used, with socio-demographic controls added. An interaction term between site and trial indicator variables will be added to test overall outcome differences over time between intervention and non-intervention clusters and across engagement arms. Additionally, comparisons across engagement arms will be conducted to determine efficacies of engagement strategies. These analyses will then yield coefficients which allow computation of the odds and marginal odds ratios for each corresponding factor on the community attitude/knowledge/acceptance outcome measure referenced from baseline. Lastly, differences in number of positive and negative feedback submissions through NEA's public feedback portal will be compared across intervention and non-intervention clusters and across the various engagement arms.

Statistical analysis: exploratory endpoint—impact of male *Wolbachia-Aedes* deployments on the circulation of dengue serotypes and diagnosed secondary infections Dengue test samples sent to the Environmental Health Institute will be further sent for serotyping and diagnosis of secondary infections as part of routine dengue surveillance based on the laboratory procedures described in the preceding sections. We will monitor trends in the circulation of specific dengue serotypes and secondary infection cases in intervention and non-intervention clusters, before, during, and after male *Wolbachia-Aedes* deployments have taken place. For assessing the serotype-specific efficacy of *Wolbachia-Aedes* deployments, we will employ a similar logistic regression strategy to estimate the intervention effect of male *Wolbachia-Aedes* by calculating the aggregate

odds ratio, which compares the exposure odds among serotype-specific test-positive cases versus test-negative controls (see above).

For assessing the efficacy of *Wolbachia-Aedes* deployments on preventing secondary dengue infection, we will employ the logistic regression to estimate the intervention effect of male *Wolbachia-Aedes* by calculating the aggregate odds ratio, which compares the exposure odds among test-positive cases who are also flagged as secondary infections versus test-negative controls (see above).

Trial governance and safety

The principal investigator, together with a Trial Steering Committee (TSC) comprising members from NEA, Singapore, and the Ministry of Health, Singapore, will be responsible for ensuring the study is performed in compliance with the approved protocol and the principles of Good Clinical Practice, and will also oversee the coordination of the trial process and data analysis for the trial. The TSC will meet quarterly or as needed throughout the trial. Day-to-day support for the trial, including production, releases, data management, and data analysis, will be overseen by a project team within NEA's Environmental Health Institute, which meets monthly.

A Review Committee (RC) will be constituted from local and international experts external to the institutions involved in the trial. Its primary role is to ensure the safety and efficacy of the intervention during the trial, as well as overall compliance and conduct of the trial. The RC can provide recommendations to the TSC on continuing/discontinuing the trial and may also make recommendations to the TSC relating to trial procedures, protocol and data management, and quality control as well as analysis. Any proposed major changes to trial protocol will be reviewed by the RC, and approval for a protocol amendment will be sought from the relevant institutional review boards (IRBs) prior to their implementation. Responsibilities and terms of reference will be set out in an RC agreement and agreed to by all RC members prior to study commencement. The RC will meet at study initiation, at 6 months following the commencement of data collection, and at attainment of 50% of the estimated minimum required number of dengue tests (n = 1000), as well as any other time at the request of the TSC.

A Data Monitoring Committee (DMC) is not constituted due to the low-risk nature of the intervention. The trial is not considered human biological research, as advised by the Ministry of Health; all laboratory tests will be performed for clinically directed reasons; and data from these tests is routinely collected as part of routine dengue surveillance under the Infectious Diseases Act[72].

Interim analyses and stopping rules

An interim analysis of the primary endpoints will be conducted when data collection reaches 25%, 50%, and 75% of the estimated minimum required number of dengue tests (*n* = 500, 1,000, 1,500). Upon interim analysis at the 50% mark, results will be communicated to the RC, and RC may recommend modification/termination of the study if data analysis shows that exposure to male *Wolbachia-Aedes* confers a reduced risk of dengue in the primary endpoints. We follow Anders et al.,2018[47] and will employ the $p < 0.01$ cutoff at interim analysis to be used as guidance for considering early termination. RC may also recommend trial termination if preliminary results at the 50% mark suggest that male *Wolbachia-Aedes* deployments are associated with excess dengue incidence/cases. A less conservative $p < 0.05$ cutoff will be used as guidance for the latter, if results show that the association is in the direction of harm. Termination or modification may also be recommended for any other operational reason (e.g., data collection rates), perceived safety concerns, or external factors. The final   to terminate or modify the study rests with the TSC.

References

1. Wilder-Smith, A. et al. Epidemic arboviral diseases: priorities for research and public health. The Lancet Infectious Diseases 17, e101–e106 (2017).
2. Gubler, D. J. Dengue, Urbanization and Globalization: The Unholy Trinity of the 21st Century. Trop Med Health 39, 3–11 (2011).
3. Cattarino, L., Rodriguez-Barraquer, I., Imai, N., Cummings, D. A. T. & Ferguson, N. M. Mapping global variation in dengue transmission intensity. Science Translational Medicine 12, eaax4144 (2020).
4. Messina, J. P. et al. Global spread of dengue virus types: mapping the 70 year history. Trends in Microbiology 22, 138–146 (2014).
5. Shepard, D. S., Undurraga, E. A., Halasa, Y. A. & Stanaway, J. D. The global economic burden of dengue: a systematic analysis. The Lancet Infectious Diseases 16, 935–941 (2016).
6. Musso, D. & Gubler, D. J. Zika Virus. Clinical Microbiology Reviews 29, 487–524 (2016).
7. Pielnaa, P. et al. Zika virus-spread, epidemiology, genome, transmission cycle, clinical manifestation, associated challenges, vaccine and antiviral drug development. Virology 543, 34–42 (2020).
8. Ng, L. C. & Hapuarachchi, H. C. Tracing the path of Chikungunya virus—Evolution and adaptation. Infection, Genetics and Evolution 10, 876–885 (2010).
9. Puntasecca, C. J., King, C. H. & LaBeaud, A. D. Measuring the global burden of chikungunya and Zika viruses: A systematic review. PLOS Neglected Tropical Diseases 15, e0009055 (2021).
10. Goh, V. S. L., Mok, C.-K. & Chu, J. J. H. Antiviral Natural Products for Arbovirus Infections. Molecules 25, 2796 (2020).
11. Londono-Renteria, B., Troupin, A. & Colpitts, T. M. Arbovirosis and potential transmission blocking vaccines. Parasites Vectors 9, 516 (2016).
12. Douam, F. & Ploss, A. Yellow Fever Virus: Knowledge Gaps Impeding the Fight Against an Old Foe. Trends in Microbiology 26, 913–928 (2018).
13. Wilder-Smith, A. et al. Deliberations of the Strategic Advisory Group of Experts on Immunization on the use of CYD-TDV dengue vaccine. The Lancet Infectious Diseases 19, e31–e38 (2019).

14. Sim, S., Ng, L. C., Lindsay, S. W. & Wilson, A. L. A greener vision for vector control: The example of the Singapore dengue control programme. PLOS Neglected Tropical Diseases 14, e0008428 (2020).
15. Achee, N. L. et al. A Critical Assessment of Vector Control for Dengue Prevention. PLOS Neglected Tropical Diseases 9, e0003655 (2015).
16. Vanlerberghe, V. et al. Community involvement in dengue vector control: cluster randomised trial. BMJ 338, b1959 (2009).
17. Moyes, C. L. et al. Contemporary status of insecticide resistance in the major Aedes vectors of arboviruses infecting humans. PLOS Neglected Tropical Diseases 11, e0005625 (2017).
18. Bowman, L. R., Donegan, S. & McCall, P. J. Is Dengue Vector Control Deficient in Effectiveness or Evidence?: Systematic Review and Meta-analysis. PLOS Neglected Tropical Diseases 10, e0004551 (2016).
19. Bourtzis, K. et al. Harnessing mosquito–Wolbachia symbiosis for vector and disease control. Acta Tropica 132, S150–S163 (2014).
20. Wang, G.-H. et al. Combating mosquito-borne diseases using genetic control technologies. Nat Commun 12, 4388 (2021).
21. Werren, J. H. BIOLOGY OF WOLBACHIA. Annual Review of Entomology 42, 587–609 (1997).
22. Crawford, J. E. et al. Efficient production of male Wolbachia-infected Aedes aegypti mosquitoes enables large-scale suppression of wild populations. Nat Biotechnol 38, 482–492 (2020).
23. Kittayapong, P. et al. Combined sterile insect technique and incompatible insect technique: The first proof-of-concept to suppress Aedes aegypti vector populations in semi-rural settings in Thailand. PLOS Neglected Tropical Diseases 13, e0007771 (2019).
24. Consortium, T. P. W.-S. & Ching, N. L. Wolbachia-mediated sterility suppresses Aedes aegypti populations in the urban tropics. 2021.06.16.21257922 Preprint at https://doi.org/10.1101/2021.06.16.21257922 (2021).
25. Zheng, X. et al. Incompatible and sterile insect techniques combined eliminate mosquitoes. Nature 572, 56–61 (2019).
26. Lim, J. T. et al. Hyperendemicity associated with increased dengue burden. Journal of The Royal Society Interface 18, 20210565 (2021).
27. Ong, J. et al. A novel entomological index, Aedes aegypti Breeding Percentage, reveals the geographical spread of the dengue vector in Singapore and serves as a spatial risk indicator for dengue. Parasites Vectors 12, 17 (2019).
28. Hapuarachchi, H. C. et al. Transient transmission of Chikungunya virus in Singapore exemplifies successful mitigation of severe epidemics in a vulnerable population. International Journal of Infectious Diseases 110, 417–425 (2021).
29. Ng, L.-C. et al. Entomologic and Virologic Investigation of Chikungunya, Singapore - Volume 15, Number 8—August 2009 - Emerging Infectious Diseases journal - CDC. doi:10.3201/eid1508.081486.
30. Fisher, D. & Cutter, J. The inevitable colonisation of Singapore by Zika virus. BMC Med 14, 188 (2016).

31. Ho, Z. J. M. et al. Outbreak of Zika virus infection in Singapore: an epidemiological, entomological, virological, and clinical analysis. The Lancet Infectious Diseases 17, 813–821 (2017).
32. Tan, L. K. et al. Force of Infection and True Infection Rate of Dengue in Singapore: Implications for Dengue Control and Management. American Journal of Epidemiology 188, 1529–1538 (2019).
33. Rajarethinam, J. et al. Dengue in Singapore from 2004 to 2016: Cyclical Epidemic Patterns Dominated by Serotypes 1 and 2. The American Journal of Tropical Medicine and Hygiene 99, 204–210 (2018).
34. News Releases. https://www.nea.gov.sg/media/news/news/index/weekly-dengue-cases-at-the-lowest-in-2020-as-community-rallied-to-fight-dengue-in-historic-outbreak-year.
35. Lim, J. T. et al. Increased Dengue Transmissions in Singapore Attributable to SARS-CoV-2 Social Distancing Measures. The Journal of Infectious Diseases 223, 399–402 (2021).
36. Lim, J. T. et al. Decreased dengue transmission in migrant worker populations in Singapore attributable to SARS-CoV-2 quarantine measures. Journal of Travel Medicine 28, taaa228 (2021).
37. Lim, J. T. et al. Impact of sars-cov-2 interventions on dengue transmission. PLOS Neglected Tropical Diseases 14, e0008719 (2020).
38. Ryan, P. A. et al. Establishment of wMel Wolbachia in Aedes aegypti mosquitoes and reduction of local dengue transmission in Cairns and surrounding locations in northern Queensland, Australia. Preprint at https://doi.org/10.12688/gatesopenres.13061.2 (2020).
39. Utarini Adi et al. Efficacy of Wolbachia-Infected Mosquito Deployments for the Control of Dengue. New England Journal of Medicine 384, 2177–2186 (2021).
40. enb-quarterly_jan-2017-vol-43-no-1-(final-version).pdf.
41. Bull, J. J. & Turelli, M. Wolbachia versus dengue: Evolutionary forecasts. Evolution, Medicine, and Public Health 2013, 197–207 (2013).
42. Phase 5 Field Study. https://www.nea.gov.sg/corporate-functions/resources/research/wolbachia-aedes-mosquito-suppression-strategy/phase-5-field-study.
43. Lim, J. T. et al. Efficacy of Wolbachia-mediated sterility to reduce the incidence of dengue: a synthetic control study in Singapore. Lancet Microbe S2666-5247(23)00397-X (2024) doi:10.1016/S2666-5247(23)00397-X.
44. Lim, J. T. et al. Efficacy of Wolbachia-mediated sterility for control of dengue: emulation of a cluster randomized target trial. 2023.11.29.23299172 Preprint at https://doi.org/10.1101/2023.11.29.23299172 (2023).
45. Bansal, S. et al. Efficacy of Wolbachia-mediated sterility to suppress adult Aedes aegypti populations. 2023.11.24.23298996 Preprint at https://doi.org/10.1101/2023.11.24.23298996 (2023).
46. Soh, S. et al. Economic impact of dengue in Singapore from 2010 to 2020 and the cost-effectiveness of Wolbachia interventions. PLOS Global Public Health 1, e0000024 (2021).
47. Anders, K. L. et al. The AWED trial (Applying Wolbachia to Eliminate Dengue) to assess the efficacy of Wolbachia-infected mosquito deployments to reduce dengue incidence in Yogyakarta, Indonesia: study protocol for a cluster randomised controlled trial. Trials 19, 302 (2018).

48. De Serres, G., Skowronski, D. M., Wu, X. W. & Ambrose, C. S. The test-negative design: validity, accuracy and precision of vaccine efficacy estimates compared to the gold standard of randomised placebo-controlled clinical trials. Euro Surveill 18, 20585 (2013).
49. Vandenbroucke, J. P. & Pearce, N. Case-control studies: basic concepts. Int J Epidemiol 41, 1480–1489 (2012).
50. Greenland, S. & Thomas, D. C. On the need for the rare disease assumption in case-control studies. Am J Epidemiol 116, 547–553 (1982).
51. Anders, K. L. et al. Cluster-Randomized Test-Negative Design Trials: A Novel and Efficient Method to Assess the Efficacy of Community-Level Dengue Interventions. American Journal of Epidemiology 187, 2021–2028 (2018).
52. Jewell, N. P., Dufault, S., Cutcher, Z., Simmons, C. P. & Anders, K. L. Analysis of cluster-randomized test-negative designs: cluster-level methods. Biostatistics 20, 332–346 (2019).
53. Lai, Y.-L. et al. Cost-Effective Real-Time Reverse Transcriptase PCR (RT-PCR) To Screen for Dengue Virus followed by Rapid Single-Tube Multiplex RT-PCR for Serotyping of the Virus. Journal of Clinical Microbiology 45, 935–941 (2007).
54. Pok, K.-Y., Lai, Y.-L., Sng, J. & Ng, L.-C. Evaluation of Nonstructural 1 Antigen Assays for the Diagnosis and Surveillance of Dengue in Singapore. Vector-Borne and Zoonotic Diseases 10, 1009–1016 (2010).
55. Lee, C. et al. Gravitraps for Management of Dengue Clusters in Singapore. The American Journal of Tropical Medicine and Hygiene 88, 888–892 (2013).
56. Singapore Department of Statistics (DOS). Base http://www.singstat.gov.sg/.
57. MOH | Weekly Infectious Diseases Bulletin. https://www.moh.gov.sg/resources-statistics/infectious-disease-statistics/2021/weekly-infectious-diseases-bulletin.
58. Ong, J. et al. Mapping dengue risk in Singapore using Random Forest. PLOS Neglected Tropical Diseases 12, e0006587 (2018).
59. Phase 1 Small-Scale Field Study. https://www.nea.gov.sg/corporate-functions/resources/research/wolbachia-aedes-mosquito-suppression-strategy/phase-1-small-scale-field-study.
60. Tewari, P. et al. Associations between Dengue Incidence, Ecological Factors, and Anthropogenic Factors in Singapore. Viruses 15, 1917 (2023).
61. Fernandez, S. A. et al. Features of the urban environment associated with Aedes aegypti abundance in high-rise public apartments in Singapore: An environmental case-control study. PLOS Neglected Tropical Diseases 17, e0011075 (2023).
62. Sun, H. et al. Spatio-temporal analysis of the main dengue vector populations in Singapore. Parasites & Vectors 14, 41 (2021).
63. Gaw, L. Y.-F., Yee, A. T. K. & Richards, D. R. A High-Resolution Map of Singapore's Terrestrial Ecosystems. Data 4, 116 (2019).

64. Landsat Normalized Difference Vegetation Index | U.S. Geological Survey. https://www.usgs.gov/landsat-missions/landsat-normalized-difference-vegetation-index.
65. OneMap. OneMap http://www.onemap.gov.sg.
66. Park, S. H. et al. Ethnicity, Neighborhood and Individual Socioeconomic Status, and Obesity: The Singapore Multiethnic Cohort. Obesity (Silver Spring) 28, 2405–2413 (2020).
67. Kolimenakis, A. et al. The role of urbanisation in the spread of Aedes mosquitoes and the diseases they transmit—A systematic review. PLOS Neglected Tropical Diseases 15, e0009631 (2021).
68. ECMWF | Advancing global NWP through international collaboration. https://www.ecmwf.int/.
69. Indriani, C. et al. Reduced dengue incidence following deployments of Wolbachia-infected Aedes aegypti in Yogyakarta, Indonesia: a quasi-experimental trial using controlled interrupted time series analysis. Preprint at https://doi.org/10.12688/gatesopenres.13122.1 (2020).
70. Deen, M. & de Rooij, M. ClusterBootstrap: An R package for the analysis of hierarchical data using generalized linear models with the cluster bootstrap. Behav Res Methods 52, 572–590 (2020).
71. Wing, C., Simon, K. & Bello-Gomez, R. A. Designing Difference in Difference Studies: Best Practices for Public Health Policy Research. Annual Review of Public Health 39, 453–469 (2018).
72. Infectious Diseases Act 1976 - Singapore Statutes Online. https://sso.agc.gov.sg:5443/Act/IDA1976.

Initial protocol and statistical analysis plan

<u>Protocol and statistical analysis plan</u>

Title: Assessing the efficacy of male *Wolbachia*-infected mosquito deployments to reduce dengue incidence in Singapore

Version: 1.0

Trial registration identifier: This protocol was registered on ClinicalTrials.gov as NCT05505682

Protocol Contributors:

Lee Ching Ng	Environmental Health Institute, National Environment Agency, Singapore
Shuzhen Sim	Environmental Health Institute, National Environment Agency, Singapore
Tan Cheong Huat	Environmental Health Institute, National Environment Agency, Singapore
Chee Seng Chong	Environmental Health Institute, National Environment Agency, Singapore
Judith Chui Ching Wong	Environmental Health Institute, National Environment Agency, Singapore
Yee Ling Lai	Environmental Health Institute, National Environment Agency, Singapore
Kathryn Vasquez	Environmental Health Institute, National Environment Agency, Singapore
Youming Ng	Environmental Health Institute, National Environment Agency, Singapore
Yvonne Wong	Environmental Health Institute, National Environment Agency, Singapore
Yin Xiang Setoh	Environmental Health Institute, National Environment Agency, Singapore
Janet Ong	Environmental Health Institute, National Environment Agency, Singapore
Caleb Lee	Environmental Health Institute, National Environment Agency, Singapore
Lu Deng	Environmental Health Institute, National Environment Agency, Singapore
Li Yun Tan	Environmental Health Institute, National Environment Agency, Singapore
Soon Hoe Ho	Environmental Health Institute, National Environment Agency, Singapore
Grace Chian	Environmental Health Institute, National Environment Agency, Singapore
Lim Jue Tao	Lee Kong Chian School of Medicine, Nanyang Technological University, Singapore<br>Environmental Health Institute, National Environment Agency, Singapore
Dengue Expert Advisory Panel	
Duane J. Gubler	Duke-NUS Medical School, National University of Singapore, Singapore
Ary Hoffmann	Monash University, Australia
Christl Donnelly	Imperial College London, United Kingdom
Chia Kee Seng	Saw Swee Hock School of Public Health, National University of Singapore, Singapore
Neil Ferguson	Imperial College London, United Kingdom
Tim Barkham	National Centre for Infectious Diseases, Singapore
Vernon J Lee	Ministry of Health, Singapore

Trial Sponsor: Environmental Health Institute, National Environment Agency, Singapore

Synopsis

Primary registry and trial identification number	This protocol was registered on ClinicalTrials.gov as NCT05505682
Date of registration in primary registry	August 18 2022
Financial support	Singapore Ministry of Finance, Ministry of Sustainability, and the National Environment Agency, and the Singapore National Robotics Program.
Sponsor	Environmental Health Institute, National Environment Agency, Singapore
Title	Assessing the efficacy of male Wolbachia-infected mosquito deployments to reduce dengue incidence in Singapore
Study setting	Singapore, Singapore
Health condition(s) study	Dengue
Intervention	Intervention arm: Deployment of male Wolbachia-infected Aedes aegypti mosquitoes, in addition to standard practice dengue control activities.<br><br>Comparison arm: Standard practice dengue control activities.
Primary endpoints	3. Symptomatic, virologically-confirmed dengue virus (DENV) infection of any severity.<br><br>4. Township-level dengue incidence rate
Secondary endpoints	3. Prevalence of *Ae. aegypti/Ae. albopictus* mosquitoes<br><br>4. Public Attitudes, Perceptions & Knowledge (APK) of *Wolbachia* and other vector control interventions
Study design	Study type: intervention study with test-negative design<br>Allocation: cluster randomised<br>Assignment: parallel, 8 intervention, 7 control arms<br>Masking: non-blinded<br>Primary purpose: prevention
Study duration	24 months, with possibility of extension of up to 3 years
Target sample size	First primary endpoint: 1600 test-negative controls and 400 dengue-positive cases will be needed to detect a 50% reduction in dengue incidence with 80% power.<br><br>Second primary endpoint: At least 31 months would be required post-intervention to detect a 50% reduction in dengue incidence rates at 80% power.
Analysis	Logistic regression model will be used to estimate the odds ratio of dengue in Wolbachia treated versus untreated clusters.<br><br>The intention-to-treat analysis will consider *Wolbachia* exposure as binary depending on the allocation of the cluster of residence.

Background

In recent years, outbreaks of arboviruses transmitted by *Aedes* mosquitoes, such as dengue, Zika, and yellow fever, have become increasingly common around the world[1]. Key factors for the rise in incidence include increased urbanization, globalization, and long-distance travel, which encourage the spread and endemicity of the viruses and their vectors, particularly the peri-domestic *Aedes aegypti* species[1,2] At present, dengue is the most widespread arboviral disease worldwide and is endemic throughout the tropical belt[3,4]. An estimated 105 million dengue infections occur annually with associated costs of around USD8.9 billion[3,5]. Other arboviruses that have dominated headlines include Zika and chikungunya: Zika caused a global epidemic in 2016 with more than 1.5 million infections in over 70 countries[6,7], with transmission still recorded by more than 80 countries in 2019 alone[7]. Meanwhile, chikungunya outbreaks have occurred frequently since the first major outbreak in 2004, which spread from Kenya to Reunion Island, followed by South and Southeast Asia by 2008, and are estimated to have caused an average yearly loss of over 106,000 DALYs globally from 2010 to 2019[8,9].

Current treatments for arboviral infections attempt to alleviate the symptoms of infection but do not address the root cause[10]. With the exception of yellow fever and, more recently, dengue, no suitable vaccine is available for arboviruses borne by *Ae. aegypti*[11]. However, vaccination rates for yellow fever are decreasing[12], and the sole licensed dengue vaccine at present, Sanofi-Pasteur's CYD-TDV, also known as Dengvaxia®, is plagued with safety concerns among immunologically naïve individuals[13]. As such, vector control remains the primary tool for mitigating the spread of arboviruses. Conventional vector control measures include source reduction[14], space spray, and targeted indoor residual spraying with insecticides to kill adult mosquitoes and chemical treatment of containers to kill larvae[15]. These are complemented with enforcement and legislation such as regular home inspections for potential breeding sites, educational campaigns, and community engagement for better environmental management[14,16]. A major drawback of conventional vector control efforts is the need for thorough and sustained implementation in order to successfully bring down the vector populations[15]. Moreover, vector resistance to insecticides may arise with prolonged use[17]. A recent meta-analysis of conventional vector control measures against dengue vectors showed that their efficacy is limited in some cases[18]. Therefore, there is a pertinent need for novel vector control strategies to mitigate the spread of arboviral diseases.

One novel approach is the incompatible insect technique (IIT), which involves the release of male mosquitoes infected with the maternally inherited intracellular bacterium *Wolbachia*[19–21]. Mating between infected males and uninfected females results in non-viable offspring through cytoplasmic incompatibility (CI), leading to suppression of the mosquito population[20,21]. *Wolbachia*-mediated IIT as a method of vector control has been tested in China, the USA, Thailand, and Singapore, the country of focus for this study protocol[22–25]. Singapore, among other programs that also attempt to control the *Ae. aegypti* population, has coupled IIT with sterile insect technique (SIT), using irradiation to sterilize residual females (due to imperfect sex sorting) before releases of *Wolbachia*-infected males. IIT-SIT reduces the likelihood of establishment of the released *Wolbachia* strain in field mosquito populations, which would render CI ineffective.

Singapore is an equatorial city-state in Southeast Asia where *Ae. aegypti* is ubiquitous and dengue is hyperendemic[26,27]. Besides dengue, the country has also reported sporadic chikungunya infections since 2013, following an outbreak in 2008 and 2013[28,29] and experienced a Zika outbreak in 2016[30,31]. Decades of conventional vector control efforts have contributed to falling dengue seroprevalence rates which has rendered the population vulnerable to dengue outbreaks despite a low *Ae. aegypti* population[32,33]. The most recent outbreak in 2020 was the largest on record with an all-time high of 1792 weekly reported cases[34], which was partially attributable to non-pharmaceutical interventions motivated by the concurrent SARS-CoV-2 outbreak[35–37].

Singapore has since 2016 been conducting phased field trials to evaluate the use of IIT-SIT to suppress the local *Ae. aegypti* population. IIT-SIT was chosen over an alternative *Wolbachia* dengue control strategy involving introgression of the bacterium into field mosquito populations to reduce their ability to transmit dengue. This latter approach, which requires release of both male and female *Wolbachia*-infected mosquitoes, has shown promise in trials in Australia and Indonesia[38,39]. However, IIT-SIT harmonizes with Singapore's decades-long vector control program which focuses on suppressing the local mosquito population. It received greater social acceptance since it does not release biting female mosquitoes and has negligible ecological impact[40]. Additionally, IIT-SIT is not subject to the risk of dengue mutants

escaping the inhibitory effect of *Wolbachia*[41]. The trials have thus far demonstrated reductions of *Ae. aegypti* populations and dengue incidences by 98% and 88%, respectively[24,42]. A modeling study has promisingly found that a *Wolbachia*-mediated IIT strategy that was conservatively 40% efficacious would have retrospectively averted an estimated USD330 million in economic costs over the ten-year period between 2010 and 2020[43].

Building on the success of the phased field studies, the efficacy of IIT-SIT in reducing dengue incidence will next be tested in a national level cluster-randomized (CR) controlled trial by adapting the methods of Anders et al.,2018[44]. Here, we present the protocol of the planned study which, to our knowledge, would be the first CR controlled trial to experimentally measure the efficacy of IIT-SIT in reducing dengue transmission.

Key study characteristics

Research Question Does large-scale deployment of male *Wolbachia*-infected *Ae. aegypti* (*Wolbachia-Aedes*) mosquitoes lead to a significant reduction in dengue incidence in people living in release areas, compared to control areas without release?

Rationale *Wolbachia-Aedes* deployments mosquitoes have previously been demonstrated to suppress field *Aedes aegypti* and *albopictus* populations[24,45–47]. Previous field studies have also retrospectively demonstrated the protective effect of *Wolbachia-Aedes* deployments on dengue**.**

Th aim of the study outlined in this protocol, is to experimentally ascertain the efficacy of large-scale *Wolbachia-Aedes* deployments in reducing dengue virus transmission in field conditions. Cluster randomised trials (cRCT) represent the gold standard design to provide evidence on the efficacy of a field-based intervention. The value of estimating the epidemiological impact of *Wolbachia* using a cRCT approach is as follows: (**1**) RCTs are the gold standard and preferred method for estimating the benefit/harm of public health interventions. (**2**) RCTs have highest weight in shaping policy and practice in public health compared to observational studies and this is likely to be true for the *Wolbachia-Aedes* technology.

Overview The aim of this study is to determine whether large-scale deployment of *Wolbachia*-infected male *Ae. aegypti* mosquitoes (the intervention, thereafter referred to as male *Wolbachia-Aedes*) can reduce the incidence of dengue in individuals living in intervention (treatment) clusters, compared to individuals living in non-intervention clusters. The two primary endpoints are (1) odds ratio of *Wolbachia* exposure distribution (i.e., probability of living in an intervention cluster) among laboratory-confirmed reported dengue cases compared to test-negative controls, and (2) laboratory-confirmed reported dengue case counts normalized by population size in intervention versus non-intervention clusters. Secondary endpoints include the efficacy of male *Wolbachia-Aedes* deployment in reducing *Ae. aegypti* mosquito populations, the impact of male *Wolbachia-Aedes* deployment on *Ae. albopictus* mosquito populations, and the pre-post trial community attitudes/knowledge and acceptance of male *Wolbachia-Aedes* and other vector control practices. Exploratory endpoints include the impact of male *Wolbachia-Aedes* deployment on the circulation of dengue cases of specific serotypes and on secondary infections.

Study Objectives

Primary objectives:

- To assess the efficacy of large scale *Wolbachia-Aedes* deployments in reducing the risk of symptomatic, virologically-confirmed dengue cases of any severity in Singapore in release areas, relative to non-release areas.
- To assess the efficacy of large scale *Wolbachia-Aedes* deployments in reducing the incidence of symptomatic, virologically-confirmed dengue cases of any severity in Singapore in release areas, relative to non-release areas.

Secondary objectives:

- To assess the efficacy of large scale *Wolbachia-Aedes* deployments in reducing the abundance of *Aedes aegypti* in Singapore in release areas, relative to non-release areas.
- To assess the effect of large scale *Wolbachia-Aedes* deployments on the abundance of *Aedes albopictus* in Singapore in release areas, relative to non-release areas.
- To assess the changes in Public Attitudes, Perceptions & Knowledge (APK) of *Wolbachia* and other vector control interventions in release and non-release areas.

Study design

Type of study This is a parallel two-arm non-blinded cluster randomised controlled trial which will be conducted in a single site in Singapore, an equatorial city-state. The study site will be subdivided into 17 non-contiguous clusters, approximately 0.79km^2 in size (range 0.53km^2-1.33km^2) and 114 high-rise public apartment blocks (range 70–173).

Clusters will be randomly allocated to receive *Wolbachia-Aedes* deployments or no intervention, such that 8 clusters receive *Wolbachia* deployments and 7 receive no intervention.

Where possible, manmade or natural borders such as major roads, highways, and water bodies will be used to delineate cluster boundaries to limit spillover of male *Wolbachia-Aedes* from intervention to non-intervention clusters, as well as migration of wildtype mosquitoes into clusters. In the absence of such borders, adjacent areas within a 300m radius were designated as bufer release areas, should the cluster be designated for intervention; clusters (inclusive of buffer areas) were kept at least 700 m apart. Conventional vector control activities by public health agencies, such as media engagement, home inspections, breeding site destruction, and space spray in response to dengue cluster, will continue as per routine practice across the study area and duration in both intervention and non-intervention clusters.

The impact of *Wolbachia-Aedes* deployments on dengue risk will be assessed by comparing the exposure distribution (probability of living in a *Wolbachia-Aedes* treated area) among test-positive dengue cases presenting to any public hospital, polyclinic or general practitioner for febrile illness, against the exposure distribution among patients with febrile illness presenting to the same network of public healthcare institution. Dengue test-positives and test-negatives will be sampled concurrently from the population of patients presenting with febrile illness to the primary healthcare network, with case or control status retrospectively identified based on the results of laboratory diagnostic testing.

The distribution of *Wolbachia* exposure in the sampled test-negative controls will reflect the distribution of *Wolbachia* exposure in the underlying source population that gave rise to test-positive cases. This will be met, so long that the core that the relative propensity to seek healthcare for febrile illness at a primary healthcare insitution in intervention versus untreated arms is the same for dengue cases as other febrile illness test-negative controls. This should be upheld if dengue cases and other febrile illness controls are clinically indistinguishable until laboratory diagnosis. The concurrent sampling of test-positives and negatives mean that the odds of *Wolbachia-Aedes* exposure among sampled dengue test-positive cases relative to test-negative controls (odds ratio), is an unbiased estimate of the relative incidence of dengue in *Wolbachia*-intervened versus non-intervened clusters, and can thereby be used to directly estimate the protective efficacy of *Wolbachia-Aedes*. The null hypothesis is that the relative incidence of virologically-confirmed dengue in *Wolbachia-Aedes* treated and untreated areas is one (i.e the computed odds ratio equals 1). If *Wolbachia-Aedes* has a protective effect against dengue, the incidence rate ratio for virologically-confirmed dengue in *Wolbachia*-treated areas versus untreated areas will be less than one.

Justification of study design cRCTs are the gold standard study design to provide evidence on the efficacy of a field-based intervention, and involves allocating the intervention or control to clusters. In this case, *Wolbachia-Aedes* deployments are randomly allocated to intervention clusters spatially and control clusters receive no intervention. The traditional implementation of cRCT under an endpoint of infection/disease incidence would be measured using a prospective cohort of individuals residing in treated/untreated clusters, and the measure of effect would be a risk or rate ratio in treated/untreated arms. Given the historically low force-of-infection for dengue and number of cases due to successful vector-control in the study setting[32], an untenably large cohort of individuals followed for a long duration would be required to detect the requisite number of dengue infections for sufficient power.

Therefore, we employed an alternative, cluster-randomized test-negative design to measure the efficacy of *Wolbachia-Aedes* deployments, to concurrently sample test-positive dengue cases and test-negative controls reporting from other febrile illness from the study population to derive the estimate of the incidence rate ratio. By leveraging on nationally representative dengue surveillance and testing databases comprising all public hospitals, polyclinics and general practitioners, we could obtain information on individuals whom present to these insitutions with febrile illness, with dengue test-positives classified as cases and test-negatives classified as controls. Here, the controls are drawn from the same source population as the cases, thus avoiding the common limitation of control selection in traditional case-control studies that can introduce selection bias[48]. Thereafter, computation of the odds ratio would provide an unbiased estimate of the rate ratio in the source population over the period of participant enrolment, without requiring any rare disease assumptions[49,50]. This test-negative design has the advantage of being more efficient, cost effective, and logistically simpler to achieve than a large prospective cohort, and can be conducted easily using observational information from nationally-representative testing databases.

Number of participants The study area comprises of approximately 722,204 residents in 15 non-contiguous clusters comprising high-rise public apartment blocks. It is estimated that 1,600 test-negative controls and 400 dengue-positive cases will be needed to detect a 50% reduction in dengue incidence with 80% power. Sample size requirements will be re-estimated using observed data after attaining 50% of the target data collection. We follow[44,51,52] to estimate the sample size for the proposed study design. We used the comparison of exposure odds among test-positive cases vs. test-negative controls for data aggregated across all clusters, with the null hypothesis that the odds of residence in a treatment cluster is the same among test-positive cases as test-negative controls[44,51,52]. This provides an unbiased estimate of the relative risk between groups as demonstrated previously[44,51,52]. We performed simulations (n = 1,000) to estimate the power to detect a 50% intervention efect, assuming eight clusters per arm, and using available historical dengue testing data in the 15 study clusters. We randomly allocated eight clusters to receive the intervention; this random allocation was repeated 10,000 times, and allocations were only kept when constrained randomization criterion were met (n = 3,151 possible balanced allocations). In each iteration of the simulation, we randomly selected one distinct intervention allocation and a 2-year time frame from the historical dengue testing data and simulated a 50% intervention efect in the intervention arm in the latter 12 months. The simulated data was analyzed using logistic regression to assess the signifcance of intervention efect. These were repeated for various dengue test sample sizes. We computed the proportion of signifcant results obtained in each scenario. This provided an estimate for the power of our study and the sample size required to obtain the respective power[44].

Expected study duration Deployment of male *Wolbachia-Aedes* and data collection from diagnostic laboratories is expected to commence in July 2022 (see Table 1). Data collection will continue for least 24 months or longer if needed to reach the minimum sample size required for intention-to-treat analysis. To account for year-to-year variability in dengue cases, data collection will continue for at least 24 months even if the estimated minimum sample size is attained sooner.

Key Activities	Period
Randomization	Feb 2022
Engage healthcare institutions for data collection	March 2022–June 2022
Community Engagement	June 2022 onwards
Start of RCT	July 2022
Midterm review	July 2023 onwards
End of RCT	September 2024
Final Analysis	September 2024–December 2024
Publication of results	December 2024

Table 1: Trial timeline

Primary outcomes: dengue The primary outcome measure will be virologically confirmed dengue virus infection in patients reporting febrile illness. All dengue-suspected patients will be classified as dengue cases using an internally controlled RTqPCR assay, dengue non-structural protein 1 (NS1) or IgM as diagnostic assays to detect dengue virus[44,53,54]. Dengue cases are patients with virologically confrmed DENV infection through RTqPCR, testing positive for NS1 antigen or IgM, according to Ministry of Health, Singapore (MOH) criteria. A positive test for any of the three assays would classify the patient as a dengue case. Controls are patients with negative test results for DENV through RT-qPCR, NS1 antigen ELISA, or DENV IgM.

Secondary outcome: *Aedes aegypti* and *albopictus* The secondary outcome measure will be *Aedes aegypti* and *Aedes albopictus* abundance. This will be measured using the Gravitrap index. Gravitraps are simple, hay infusion-filled cylindrical traps with a sticky lining on the inner surface designed to lure and trap gravid female *Aedes* developed by the National Environment Agency, Singapore[55]. Adult *Aedes* populations in intervention and non-intervention clusters will be monitored weekly using an average of 6 to 9 Gravitraps per high-rise apartment block[24].

Study setting The study will be conducted in high-rise public housing areas in Singapore. Singapore has an area of 728 km^2 and a population of approximately 5.7 million as of 2020[56]. The study sites cover 10.9 km^2 and have an estimated total population of 722,204, with an average population density of approximately 66,257 persons per km^2 .

Annually, the number of reported dengue cases in Singapore ranged between 51 and 621 cases per 100,000 individuals from 2010 to 2020[56,57]. Fifteen clusters were identifed as locations at high risk of dengue transmission, each with an average area of 0.79 km2 (range 0.53–1.33 km2) and 114 high-rise public apartment blocks[58] (range 70–173). 8 clusters will be randomly allocated intervention and 7 clusters will be randomly allocated as control sites which do not receive intervention.

Study intervention

Randomisation method Due to the small number of clusters available for randomization, selection of clusters will rely on constrained randomization to prevent chance imbalances in baseline historical dengue risk between intervention and non-intervention arms. The proportion of positive to negative dengue samples over 2016 to 2019 will be used as the constraining variable. We will generate a large number (n = 10,000) of potential random cluster allocations in 8:7 intervention/non-intervention ratio. For each allocation, the value of the constraining variable will be calculated in each study arm using the aggregate arm-level value. Each potential random allocation will be evaluated against the pre-defined balancing criteria (i.e., no statistically significant difference in the proportion of positive to negative dengue samples between the two arms) and removed as a potential random allocation if they were not met. All potential allocations that satisfy the balancing criteria were kept (n = 3151, exceeding the threshold of 100–150 allocations recommended in the literature), and a single allocation will be randomly selected from within the restricted list of balanced allocations. Finally, a single random draw was used to determine which of the two study arms was to receive male *Wolbachia-Aedes* releases. Randomization will be followed by extensive community engagement to seek the support of local politicians and grassroot leaders of each of the eight intervention clusters.

***Wolbachia-Aedes* deployment** Low-dose (~40 Gy or lower) X-ray-irradiated male *w*AlbB-infected *Ae. aegypti* (male *Wolbachia-Aedes*) will be released in designated public locations in high-rise housing estates in the intervention clusters[24]. Releases may commence across all intervention clusters simultaneously or, if operationally more feasible, be staggered between three lots across two months apart. Releases will be conducted twice a week, during periods of highest *Ae. aegypti* activity (weekdays between 0630–1100h and 1300–1800h)[24]. To facilitate even distribution of mosquitoes, releases will be conducted in equally spaced release locations per apartment block, on the ground, middle (levels 5–8) and high floors (≥ level 9). Based on previous studies[24], we expect to release 1–6 male mosquitoes per resident, with the release numbers adaptively guided by the Gravitrap index at each location each release week. Mosquito monitoring will continue throughout the study period. Male *Wolbachia-Aedes* will also be released twice a week on the ground floors of high- and low- rise housing estates within the designated buffer areas[59]. Where releases in buffer areas cannot be performed (e.g., schools), releases will be conducted at the boundary between such areas and the intervention sites. This is to counteract immigration of wild-type *Ae. aegypti* females from contiguous non-release areas.

***Wolbachia-Aedes* production** Eggs will be submerged in hatching broth (0.071g baker's yeast (MP Biomedicals, France), 0.357g nutrient media (CM0001 Nutrient broth, Oxoid, England) or 0.536g nutrient media (Liofilchem, Italy) and 1000ml reverse osmosis (RO) water) to induce hatching. After two hours in hatching broth aged overnight, larvae will be counted using an automated larvae counter (Orinno Technology Pte Ltd, Singapore) and transferred to rearing trays measuring 103 x 63 x 3 cm at a density of 4.7 larvae per ml of RO water, respectively. Larvae will be fed with ground ALTech Arthropod Larvae Feed (ALTech, Singapore) in slurry form. Six days post-hatching, male pupae will be separated from female pupae and larvae using the Pupae Separation System (Orinno Technology Pte Ltd, Singapore) prior irradiation for IIT-SIT releases. Pupae will be allowed to eclose inside release containers.

Irradiation will be performed with RS2400V irradiators (Rad Source Technologies Inc., USA). For IIT-SIT irradiation (female sterilization), pupae will be irradiated in plastic containers measuring 6.5 cm in diameter and 7.0 cm in height (for release). For SIT irradiation (male sterilization), pupae will be irradiated in petri dishes with diameter 9.4 cm. Petri dishes or containers will be placed at the center of the irradiation chamber of the X-ray irradiator. The effective dose will be 30 Gy to 40 Gy, depending on pupae density, irradiator model and desired sterility.

Entomological monitoring strategy Gravitraps are simple, hay infusion-filled cylindrical traps with a sticky lining on the inner surface designed to lure and trap gravid female *Aedes* developed by the National Environment Agency, Singapore[55]. Adult *Aedes* populations in intervention and non-intervention clusters will be monitored weekly using an average of 6 to 9 Gravitraps per high-rise apartment block[24]. Gravitraps are placed in public spaces along corridors of public housing apartments in all sites, at the ratio of 1 trap for every 20 households. This trap-to-household ratio and deployment was based on logistic considerations for long term monitoring, and it provides an assessment of the density as well as vertical distribution of the mosquito population. Mosquito data is collected from all Gravitraps on a bi-weekly

basis, and mosquito samples were sexed and morphologically identified. Female *Aedes* mosquitoes that cannot be speciated morphologically will be identified using a multiplex *Ae. aegypti*- and *Ae. albopictus*-specific real-time qPCR assay targeting the COI gene (*Aedes* forward 5'-TCC CGC CTT CRG TGC GCG G-3', *Aedes* reverse 5'-CGC GGG ATG TAY TCA TCA ACC-3', probe AEG Cy5 – 5'-TAG TCA GAC GTG GTG GTG ACA CAC C-3'– BHQ2, and ALBO HEX – 5'-ACG GTG GCC GGC GTG CCA GTC GT-3' – BHQ1). The multiplex qPCR reactions were performed in 20µl total volume containing 1X SensiFAST No-Rox Probe Mastermix (Bioline, USA); 0.15µM and 0.05µM COI primers and probes, respectively; 0.4 µM and 0.15µM wsp primers and probes, respectively; and 2µl. Cycling will be carried out in a Rotorgene Q (Qiagen, Germany) using the following conditions: 95°C for 5 min, 40 cycles at 95°C for 10s, and 60°C for 30s.

Furthermore, screening for the presence of *Wolbachia* in trapped female *Aedes aegypti* may be carried out to monitor for *Wolbachia* establishment. While irradiation greatly reduces the risk of establishment, this is a precautionary measure for early detection in the unlikely event that a sufficient number of fertile females are released to result in establishment. If *Wolbachia* becomes established in an intervention cluster, male *Wolbachia-Aedes* irradiated for male sterility at a higher X-ray dose (≥ 40 Gy) or male *Aedes aegypti* infected with another incompatible *Wolbachia* strain may be released to mitigate the establishment, which is expected to hamper mosquito population suppression.

Selection of patients

Selection of patients No enrolment of patients will conducted. The trial will rely on collection of data from dengue-suspected patients, whose blood samples are sent for dengue tests, to all diagnostic laboratories that support private clinics, public polyclinics, or public/private hospitals. This network is nationally representative and comprises tests of all individuals reporting for testing in public/private hospitals and public polyclinics. Dengue-suspected patients are identified by clinicians through symptoms such as high fever, body aches, and rashes, coupled with absence of respiratory symptoms. Cases and test-negative controls will be retrospectively classified through laboratory test results. In addition, we will also obtain spatially resolved counts of laboratory-confirmed dengue cases reported to the Ministry of Health. Approval from the Director of Medical Services has been obtained to collect data for the purpose of the trial.

Inclusion criteria All patients who report to testing in public/private hospitals and public polyclinics who have dengue-suspected illness.

Exclusion criteria If test data from more than one episode of febrile illness is reported for a single individual, the data will only be included if the test episodes are more than 4 weeks apart.

Data collection procedures

For epidemiologic monitoring Identifiable individual-level data on laboratory-confirmed dengue cases and dengue test-negative controls (including residential postal codes, basic socio-demographic information, and details about dengue test status) will be obtained from the national surveillance network of diagnostic laboratories which receive samples for dengue testing from private clinics, public polyclinics, and public/private hospitals. Individual-level data will first be cleaned up and checked for duplicates by selected analysts approved to access and handle identifiable data. If test data from more than one episode of febrile illness is reported for a single individual, the data will only be included if the episodes are more than 4 weeks apart. Data will then be de-identified before further analysis. In addition, we will also obtain spatially resolved counts of laboratory-confirmed dengue cases reported to the Ministry of Health. Regular data monitoring will be conducted to identify biases in response and/or missing data.

For entomologic monitoring, Gravitraps will be used. Gravitraps are simple, hay infusion-filled cylindrical traps with a sticky lining on the inner surface designed to lure and trap gravid female *Aedes* developed by the National Environment Agency, Singapore[55]. Adult *Aedes* populations in intervention and non-intervention clusters will be monitored weekly using an average of 6 to 9 Gravitraps per high-rise apartment block[24]. Gravitraps are placed in public spaces along corridors of public housing apartments in all sites, at the ratio of 1 trap for every 20 households. This trap-to-household ratio and deployment was based on logistic considerations for long term monitoring, and it provides an assessment of the density as well as vertical distribution of the mosquito population. Mosquito data is collected from all Gravitraps on a bi-weekly basis, and mosquito samples were sexed and morphologically identified. Female *Aedes* mosquitoes that cannot be speciated morphologically will be identified using a multiplex *Ae. aegypti*- and *Ae. albopictus*-specific real-time qPCR assay targeting the COI gene (*Aedes* forward 5'-TCC CGC CTT CRG TGC GCG G-3', *Aedes* reverse 5'-CGC GGG ATG TAY TCA TCA ACC-3', probe AEG Cy5 – 5'-TAG TCA GAC GTG GTG GTG ACA CAC C-3'– BHQ2, and ALBO HEX – 5'-ACG GTG GCC GGC GTG CCA GTC GT-3' – BHQ1). The multiplex qPCR reactions were performed in 20µl total volume containing 1X SensiFAST No-Rox Probe Mastermix (Bioline, USA); 0.15µM and 0.05µM COI primers and probes, respectively; 0.4 µM and 0.15µM wsp primers and probes, respectively; and 2µl. Cycling was carried out in a Rotorgene Q (Qiagen, Germany) using the following conditions: 95°C for 5 min, 40 cycles at 95°C for 10s, and 60°C for 30s.

Data management and governance Access to trial data will require a restricted VPN connection and a data access key only shared with specific individuals authorized by senior management and the trial Principal Investigator. In addition, data can only be accessed by study personnel who have completed all required cyber/data-security training. All patient data, including dengue test status and residential postal code, clinical, and sociodemographic information will be kept strictly confidential. Periodic reviews by data monitors (internal to the National Environment Agency but who are not involved in the trial) will be conducted to ensure adherence to data governance measures.

Trial data and study information will only be released under prior written approval of the Trial Steering Committee. Patient data will not be released without written permission from respective patients, except for monitoring by ethical review board or regulatory agencies in de-identified form. Reporting of study results will not permit identification or the place of residence of individual patients.

Statistical methods – sample size estimation, analytical plan for primary/secondary endpoints.

Power calculations—first primary endpoint It is estimated that 1,600 test-negative controls and 400 dengue-positive cases will be needed to detect a 50% reduction in dengue incidence with 80% power. Sample size requirements will be re-estimated using observed data after attaining 50% of the target data collection. We follow[44,51,52] to estimate the sample size for the proposed study design. We used the comparison of exposure odds among test-positive cases vs. test-negative controls for data aggregated across all clusters, with the null hypothesis that the odds of residence in a treatment cluster is the same among test-positive cases as test-negative controls[44,51,52]. This provides an unbiased estimate of the relative risk between groups as demonstrated previously[44,51,52].

We performed simulations (n = 1,000) to estimate the power to detect a 50% intervention effect, assuming eight/seven clusters per arm, and using available historical dengue testing data in the 15 study clusters. We randomly allocated eight clusters to receive the intervention; this random allocation was repeated 10,000 times, and allocations were only kept when constrained randomization criterion were met (n = 3,151 possible balanced allocations). In each iteration of the simulation, we randomly selected one distinct intervention allocation and a 2-year time frame from the historical dengue testing data and simulated a 50% intervention effect in the intervention arm in the latter 12 months. The simulated data was analyzed using logistic regression to assess the significance of intervention effect. These were repeated for various dengue test sample sizes. We computed the proportion of significant results obtained in each scenario. This provided an estimate for the power of our study and the sample size required to obtain the respective power[44].

Power calculations—second primary endpoint Intervention efficacy in the second primary endpoint from the difference-in-differences strategy as described below, laboratory-confirmed dengue case counts normalized by cluster population size (incidence rate), which is aggregated at the cluster level and measured across months and across clusters. We followed Indriani et al., 2020[60] to estimate the required post-intervention duration required to detect a 50% reduction in dengue incidence rates with 80% power.

The power calculation was conducted by performing simulations (n = 1,000) to estimate the power to detect a 50% intervention effect, assuming eight and seven clusters in the intervention and non-intervention arm respectively, and using historical dengue incidence rate data (2015–2021) in the 15 study clusters. These clusters were constrained randomized to obtain balanced intervention/non-intervention arms as delineated in the preceding sections. In each iteration of the simulation, we randomly selected one distinct intervention allocation and 0.5, 1, 1.5, 2, 2.5, and 3 year time frames post-intervention, with pre-intervention period fixed at 4 years and simulated a 50% intervention effect in the intervention arm in the following intervention. The simulated data was analyzed using the difference-in-differences specification (see below) to assess the significance of intervention effect. We then computed the proportion of significant results obtained in each scenario. This provided an estimate for the power of our study and the measurement duration required to obtain the respective power. We estimate that at least 31 months would be required post-intervention to detect a 50% reduction in dengue incidence rates at 80% power.

Pre-post trial attitudes, acceptance, and knowledge survey on *Wolbachia-Aedes* interventions and other vector control practices To assess efficacy of the various engagement strategies employed, an independent consultancy firm will be engaged to conduct door-to-door household perception surveys. These surveys will be used to measure (1) the effectiveness of different community engagement strategies and (2) each strategy's efficacy in shaping community attitudes, perception, and knowledge pre- and post-intervention. Surveyors will be trained before data collection to ensure their understanding of the objectives, methodology, expectations, and questionnaires. The surveys will be conducted in three waves, in households equally selected from the intervention and non-intervention clusters. The first survey will commence three months before and up until the beginning of *Wolbachia-Aedes* deployments. The second and third surveys will begin 3 months and 1 year following the commencement of *Wolbachia-Aedes* deployments respectively. Each survey wave will be split into three arms, (i) non-intervention clusters with no community engagement, (ii) intervention clusters with standard community engagement practices, and (iii) intervention clusters with alternate community engagement strategies. Households will be selected via stratified systematic sampling. Households will be stratified according to ethnic group denomination and minority socio-demographic factors and systemically sampled. Minority groups will be accounted for in higher proportions to reflect adequate representation of these groups. To accurately reflect the general sentiments of the working and non-working populations, surveys will be

administered during the evenings of the working days and weekends. The sampled list of households across the different timepoints will be mutually exclusive to prevent contamination of questions across waves. Survey questionnaires used will be tailored to collect specific information across various timepoints and study arms. For each survey session, the surveyor will approach one respondent from each household to interview, after obtaining informed consent to participate. In the event of (i) resident refuses to participate and (ii) resident is not home, the surveyor will note down these households and arrange for a second visit, before approaching the next household on the list. In the event of a language barrier, surveyors will note the language requirements of the household and a second visit with a surveyor equipped with the necessary language skills will return to the household to conduct the survey. A maximum of one revisit to the same household will be attempted to accurately capture ground sentiments, while remaining prudent with resources.

The National Environment Agency's (NEA) public feedback portal will also be used as an additional data source, to collect residents' feedback submissions related to *Wolbachia-Aedes* deployment in all clusters. Submissions will be classified as positive or negative and tracked through the duration of the study to supplement understanding of residents' attitudes, knowledge, and perceptions of the intervention.

Statistical analysis: primary endpoint—impact of male *Wolbachia-Aedes* deployments on dengue incidence, measured by odds ratio of *Wolbachia* exposure distribution among laboratory-confirmed reported dengue cases compared to test-negative controls In this analysis, male *Wolbachia-Aedes* exposure will be considered as a binary classification based on whether a patient's residence is in an intervention cluster or a non-intervention cluster, where residence is defined as the primary place where the patient resided at reporting date. Logistic regression will be used to assess the intervention effect of male *Wolbachia-Aedes* by estimating the aggregate odds ratio, which compares the exposure odds among test-positive cases versus test-negative controls. The null hypothesis is that the odds of residing in the intervention clusters are the same among test-positive cases as test-negative controls.

A secondary cluster-level analysis will be performed using the cluster-level summary measure of the proportion of dengue test-positive individuals among all individuals tested for dengue. The difference in proportions between the intervention/non-intervention clusters will be used to test the null hypothesis of no intervention effect based on the hypothesis tests described in Jewell et al,.2019[52]. The average proportions between treatment arms can then be used to infer the relative risk (RR) of dengue in intervention versus non-intervention arms following Anders et al.,2018[44].

Statistical analysis: primary endpoint—impact of male *Wolbachia-Aedes* deployments on dengue incidence, measured by laboratory-confirmed reported dengue case counts normalized by population size in intervention versus non-intervention clusters We will monitor trends in reported laboratory-confirmed dengue case counts in intervention and non-intervention clusters, before, during, and after male *Wolbachia-Aedes* deployments take place. We aim to use a quasi-experimental approach to examine the impact of male *Wolbachia-Aedes* deployment on routine dengue case notifications normalized by cluster population size. Namely, we will use regression models to model the monthly, normalized dengue case counts across sites; we will control for population size and seasonal variability through weather station observations and autocorrelation between sites by inclusion of lag terms. To estimate the impact of male *Wolbachia-Aedes* deployments on routine dengue case counts, the regression specification will include a binary group variable to denote whether a cluster is an intervention/non-intervention cluster, and a binary treatment variable denoted 1 will be included to indicate when interventions occur within that cluster[60]. Following similar difference-in-difference strategies[61], the interaction term between the group and treatment variables will then yield a coefficient, which provides an estimate of the intervention effect of male *Wolbachia-Aedes* deployments on monthly reported case counts. This approach ensures that extraneous factors and selection bias, such as level changes in dengue case counts in either intervention/non-intervention arms post-intervention are removed[61]. Should pre-trends assumptions in dengue case counts between intervention/non-intervention clusters be violated under this quasi-experimental approach, we will instead use synthetic control methods which can more appropriately identify the impact of male *Wolbachia-Aedes* deployments on routine dengue case notifications.

In both primary endpoints, intention-to-treat analysis will classify all individuals/clusters respectively allocated to the intervention arm in the randomization stage as such, notwithstanding any non-treatment of the intervention clusters. In per-protocol analysis, any dropouts of the intervention clusters will be removed from the analysis, and only intervention clusters receiving intervention treatment as well as the allocated controls will be analyzed.

Statistical analysis: secondary endpoint—impact of male *Wolbachia-Aedes* deployments on the prevalence of *Ae. aegypti/Ae. albopictus* mosquitoes Similarly, we will test whether male *Wolbachia-Aedes* deployments will effectively suppress *Ae. aegypti* mosquito populations as well as whether they have spillover impact on *Ae. albopictus* mosquito populations. Using the nationally representative network of Gravitraps as described above, we will employ a similar quasi-experimental approach to examine the impact of male *Wolbachia-Aedes* deployment on routinely collected *Ae. aegypti/Ae. albopictus* mosquito population data. Namely, we will use regression models with the normal link to model the mean weekly captured *Ae. aegypti/Ae. albopictus* per trap across clusters and control for seasonal variability through weather station observations and autocorrelation between clusters by inclusion of lagged mosquito population terms. To estimate the impact of male *Wolbachia-Aedes* deployments on *Ae. aegypti/Ae. albopictus* mosquito populations, the regression specification will include a binary group treatment variable to denote whether a site is an intervention/non-intervention cluster, and a binary treatment variable denoted 1 will be included to indicate when interventions occur within that cluster[60]. Following other difference-in-difference strategies[61], the intervention term between the group and treatment variables will then yield a coefficient, which provides an estimate of the intervention effect of *Wolbachia-Aedes* interventions on *Ae. aegypti/Ae. albopictus* mosquito populations weekly. This approach ensures that extraneous factors and selection bias, such as level changes in *Ae. aegypti/Ae. albopictus* mosquito populations in either intervention/non-intervention arms post-intervention are removed. Should pre-trends assumptions in *Ae. aegypti/Ae. albopictus* mosquito populations between intervention/non-intervention clusters be violated under this quasi-experimental approach, we will instead use synthetic control methods which can more appropriately identify the impact of male *Wolbachia-Aedes* deployment on the dependent variable of interest.

Statistical analysis: secondary endpoint—impact of male *Wolbachia-Aedes* deployments on community attitudes/knowledge and acceptance Using prospectively collected household survey data as described in preceding sections, we will test for differences in engagement and awareness across engagement arms in male *Wolbachia-Aedes* deployment clusters, across 3 sentiment surveys, after which differences and changes in residents' knowledge attitudes and perceptions on male *Wolbachia-Aedes* deployment and vector control practices will be evaluated across intervention and engagement arms. Responses from all survey waves across intervention/non-intervention sites will first be hypothesis tested for differences in the captured socio-demographic characteristics. If there are between-wave differences in socio-demographic characteristics, propensity score matching will be conducted and/or synthetic weights assigned to each observation to mirror the national level socio-demographic composition. To examine the impact of the trial on community endpoints, logistic/multinomial regressions with site- and trial-specific fixed effects will be used, with socio-demographic controls added. An interaction term between site and trial indicator variables will be added to test overall outcome differences over time between intervention and non-intervention clusters and across engagement arms. Additionally, comparisons across engagement arms will be conducted to determine efficacies of engagement strategies. These analyses will then yield coefficients which allow computation of the odds and marginal odds ratios for each corresponding factor on the community attitude/knowledge/acceptance outcome measure referenced from baseline. Lastly, differences in number of positive and negative feedback submissions through NEA's public feedback portal will be compared across intervention and non-intervention clusters and across the various engagement arms.

Statistical analysis: exploratory endpoint—impact of male *Wolbachia-Aedes* deployments on the circulation of dengue serotypes and diagnosed secondary infections Dengue test samples sent to the Environmental Health Institute will be further sent for serotyping and diagnosis of secondary infections as part of routine dengue surveillance based on the laboratory procedures described in the preceding sections. We will monitor trends in the circulation of specific dengue serotypes and secondary infection cases in intervention and non-intervention clusters, before, during, and after male *Wolbachia-Aedes* deployments have taken place. For assessing the serotype-specific efficacy of *Wolbachia-Aedes* deployments, we will employ a similar logistic regression strategy to estimate the intervention effect of male *Wolbachia-Aedes* by calculating the aggregate odds ratio, which compares the exposure odds among serotype-specific test-positive cases versus test-negative controls (see above).

For assessing the efficacy of *Wolbachia-Aedes* deployments on preventing secondary dengue infection, we will employ the logistic regression to estimate the intervention effect of male *Wolbachia-Aedes* by calculating the aggregate odds ratio, which compares the exposure odds among test-positive cases who are also flagged as secondary infections versus test-negative controls (see above).

Trial governance and safety

The principal investigator, together with a Trial Steering Committee (TSC) comprising members from NEA, Singapore, and the Ministry of Health, Singapore, will be responsible for ensuring the study is performed in compliance with the approved protocol and the principles of Good Clinical Practice, and will also oversee the coordination of the trial process and data analysis for the trial. The TSC will meet quarterly or as needed throughout the trial. Day-to-day support for the trial, including production, releases, data management, and data analysis, will be overseen by a project team within NEA's Environmental Health Institute, which meets monthly.

A Review Committee (RC) will be constituted from local and international experts external to the institutions involved in the trial. Its primary role is to ensure the safety and efficacy of the intervention during the trial, as well as overall compliance and conduct of the trial. The RC can provide recommendations to the TSC on continuing/discontinuing the trial and may also make recommendations to the TSC relating to trial procedures, protocol and data management, and quality control as well as analysis. Any proposed major changes to trial protocol will be reviewed by the RC, and approval for a protocol amendment will be sought from the relevant institutional review boards (IRBs) prior to their implementation. Responsibilities and terms of reference will be set out in an RC agreement and agreed to by all RC members prior to study commencement. The RC will meet at study initiation, at 6 months following the commencement of data collection, and at attainment of 50% of the estimated minimum required number of dengue tests (n = 1000), as well as any other time at the request of the TSC.

A Data Monitoring Committee (DMC) is not constituted due to the low-risk nature of the intervention. The trial is not considered human biological research, as advised by the Ministry of Health; all laboratory tests will be performed for clinically directed reasons; and data from these tests is routinely collected as part of routine dengue surveillance under the Infectious Diseases Act[62].

Interim analyses and stopping rules

An interim analysis of the primary endpoints will be conducted when data collection reaches 25%, 50%, and 75% of the estimated minimum required number of dengue tests (n = 500, 1,000, 1,500). Upon interim analysis at the 50% mark, results will be communicated to the RC, and RC may recommend modification/termination of the study if data analysis shows that exposure to male *Wolbachia-Aedes* confers a reduced risk of dengue in the primary endpoints. We follow Anders et al.,2018[44] and will employ the $p < 0.01$ cutoff at interim analysis to be used as guidance for considering early termination. RC may also recommend trial termination if preliminary results at the 50% mark suggest that male *Wolbachia-Aedes* deployments are associated with excess dengue incidence/cases. A less conservative $p < 0.05$ cutoff will be used as guidance for the latter, if results show that the association is in the direction of harm. Termination or modification may also be recommended for any other operational reason (e.g., data collection rates), perceived safety concerns, or external factors. The final decision to terminate or modify the study rests with the TSC.

References

1. Wilder-Smith, A. *et al.* Epidemic arboviral diseases: priorities for research and public health. *The Lancet Infectious Diseases* **17**, e101–e106 (2017).
2. Gubler, D. J. Dengue, Urbanization and Globalization: The Unholy Trinity of the 21st Century. *Trop Med Health* **39**, 3–11 (2011).
3. Cattarino, L., Rodriguez-Barraquer, I., Imai, N., Cummings, D. A. T. & Ferguson, N. M. Mapping global variation in dengue transmission intensity. *Science Translational Medicine* **12**, eaax4144 (2020).
4. Messina, J. P. *et al.* Global spread of dengue virus types: mapping the 70 year history. *Trends in Microbiology* **22**, 138–146 (2014).
5. Shepard, D. S., Undurraga, E. A., Halasa, Y. A. & Stanaway, J. D. The global economic burden of dengue: a systematic analysis. *The Lancet Infectious Diseases* **16**, 935–941 (2016).
6. Musso, D. & Gubler, D. J. Zika Virus. *Clinical Microbiology Reviews* **29**, 487–524 (2016).
7. Pielnaa, P. *et al.* Zika virus-spread, epidemiology, genome, transmission cycle, clinical manifestation, associated challenges, vaccine and antiviral drug development. *Virology* **543**, 34–42 (2020).
8. Ng, L. C. & Hapuarachchi, H. C. Tracing the path of Chikungunya virus—Evolution and adaptation. *Infection, Genetics and Evolution* **10**, 876–885 (2010).
9. Puntasecca, C. J., King, C. H. & LaBeaud, A. D. Measuring the global burden of chikungunya and Zika viruses: A systematic review. *PLOS Neglected Tropical Diseases* **15**, e0009055 (2021).
10. Goh, V. S. L., Mok, C.-K. & Chu, J. J. H. Antiviral Natural Products for Arbovirus Infections. *Molecules* **25**, 2796 (2020).
11. Londono-Renteria, B., Troupin, A. & Colpitts, T. M. Arbovirosis and potential transmission blocking vaccines. *Parasites Vectors* **9**, 516 (2016).
12. Douam, F. & Ploss, A. Yellow Fever Virus: Knowledge Gaps Impeding the Fight Against an Old Foe. *Trends in Microbiology* **26**, 913–928 (2018).
13. Wilder-Smith, A. *et al.* Deliberations of the Strategic Advisory Group of Experts on Immunization on the use of CYD-TDV dengue vaccine. *The Lancet Infectious Diseases* **19**, e31–e38 (2019).
14. Sim, S., Ng, L. C., Lindsay, S. W. & Wilson, A. L. A greener vision for vector control: The example of the Singapore dengue control programme. *PLOS Neglected Tropical Diseases* **14**, e0008428 (2020).
15. Achee, N. L. *et al.* A Critical Assessment of Vector Control for Dengue Prevention. *PLOS Neglected Tropical Diseases* **9**, e0003655 (2015).

16. Vanlerberghe, V. *et al.* Community involvement in dengue vector control: cluster randomised trial. *BMJ* **338**, b1959 (2009).
17. Moyes, C. L. *et al.* Contemporary status of insecticide resistance in the major Aedes vectors of arboviruses infecting humans. *PLOS Neglected Tropical Diseases* **11**, e0005625 (2017).
18. Bowman, L. R., Donegan, S. & McCall, P. J. Is Dengue Vector Control Deficient in Effectiveness or Evidence?: Systematic Review and Meta-analysis. *PLOS Neglected Tropical Diseases* **10**, e0004551 (2016).
19. Bourtzis, K. *et al.* Harnessing mosquito–*Wolbachia* symbiosis for vector and disease control. *Acta Tropica* **132**, S150–S163 (2014).
20. Wang, G.-H. *et al.* Combating mosquito-borne diseases using genetic control technologies. *Nat Commun* **12**, 4388 (2021).
21. Werren, J. H. BIOLOGY OF WOLBACHIA. *Annual Review of Entomology* **42**, 587–609 (1997).
22. Crawford, J. E. *et al.* Efficient production of male Wolbachia-infected Aedes aegypti mosquitoes enables large-scale suppression of wild populations. *Nat Biotechnol* **38**, 482–492 (2020).
23. Kittayapong, P. *et al.* Combined sterile insect technique and incompatible insect technique: The first proof-of-concept to suppress Aedes aegypti vector populations in semi-rural settings in Thailand. *PLOS Neglected Tropical Diseases* **13**, e0007771 (2019).
24. Consortium, T. P. W.-S. & Ching, N. L. Wolbachia-mediated sterility suppresses Aedes aegypti populations in the urban tropics. 2021.06.16.21257922 Preprint at https://doi.org/10.1101/2021.06.16.21257922 (2021).
25. Zheng, X. *et al.* Incompatible and sterile insect techniques combined eliminate mosquitoes. *Nature* **572**, 56–61 (2019).
26. Lim, J. T. *et al.* Hyperendemicity associated with increased dengue burden. *Journal of The Royal Society Interface* **18**, 20210565 (2021).
27. Ong, J. *et al.* A novel entomological index, Aedes aegypti Breeding Percentage, reveals the geographical spread of the dengue vector in Singapore and serves as a spatial risk indicator for dengue. *Parasites Vectors* **12**, 17 (2019).
28. Hapuarachchi, H. C. *et al.* Transient transmission of Chikungunya virus in Singapore exemplifies successful mitigation of severe epidemics in a vulnerable population. *International Journal of Infectious Diseases* **110**, 417–425 (2021).
29. Ng, L.-C. *et al.* Entomologic and Virologic Investigation of Chikungunya, Singapore - Volume 15, Number 8—August 2009 - Emerging Infectious Diseases journal - CDC. doi:10.3201/eid1508.081486.
30. Fisher, D. & Cutter, J. The inevitable colonisation of Singapore by Zika virus. *BMC Med* **14**, 188 (2016).
31. Ho, Z. J. M. *et al.* Outbreak of Zika virus infection in Singapore: an epidemiological, entomological, virological, and clinical analysis. *The Lancet Infectious Diseases* **17**, 813–821 (2017).
32. Tan, L. K. *et al.* Force of Infection and True Infection Rate of Dengue in Singapore: Implications for Dengue Control and Management. *American Journal of Epidemiology* **188**, 1529–1538 (2019).
33. Rajarethinam, J. *et al.* Dengue in Singapore from 2004 to 2016: Cyclical Epidemic Patterns Dominated by Serotypes 1 and 2. *The American Journal of Tropical Medicine and Hygiene* **99**, 204–210 (2018).
34. News Releases. https://www.nea.gov.sg/media/news/news/index/weekly-dengue-cases-at-the-lowest-in-2020-as-community-rallied-to-fight-dengue-in-historic-outbreak-year.
35. Lim, J. T. *et al.* Increased Dengue Transmissions in Singapore Attributable to SARS-CoV-2 Social Distancing Measures. *The Journal of Infectious Diseases* **223**, 399–402 (2021).

36. Lim, J. T. *et al.* Decreased dengue transmission in migrant worker populations in Singapore attributable to SARS-CoV-2 quarantine measures. *Journal of Travel Medicine* **28**, taaa228 (2021).
37. Lim, J. T. *et al.* Impact of sars-cov-2 interventions on dengue transmission. *PLOS Neglected Tropical Diseases* **14**, e0008719 (2020).
38. Ryan, P. A. *et al.* Establishment of *w*Mel *Wolbachia* in *Aedes aegypti* mosquitoes and reduction of local dengue transmission in Cairns and surrounding locations in northern Queensland, Australia. Preprint at https://doi.org/10.12688/gatesopenres.13061.2 (2020).
39. Utarini Adi *et al.* Efficacy of Wolbachia-Infected Mosquito Deployments for the Control of Dengue. *New England Journal of Medicine* **384**, 2177–2186 (2021).
40. Ng *et al*. How safe is *Wolbachia* for *Aedes* control? A risk assessment for the use of male *Wolbachia*-carrying *Aedes aegypti*for suppression of the *Aedes aegypti* mosquito population. Epidemiol News Bull. 2017;43(1):8–16..
41. Bull, J. J. & Turelli, M. Wolbachia versus dengue: Evolutionary forecasts. *Evolution, Medicine, and Public Health* **2013**, 197–207 (2013).
42. Phase 5 Field Study. National Environment Agency Singapore. Available from:https://www.nea.gov.sg/corporate-functions/resources/research/wolbachia-aedes-mosquito-suppression-strategy/phase-5-field-study.
43. Soh, S. *et al.* Economic impact of dengue in Singapore from 2010 to 2020 and the cost-effectiveness of Wolbachia interventions. *PLOS Global Public Health* **1**, e0000024 (2021).
44. Anders, K. L. *et al.* The AWED trial (Applying Wolbachia to Eliminate Dengue) to assess the efficacy of Wolbachia-infected mosquito deployments to reduce dengue incidence in Yogyakarta, Indonesia: study protocol for a cluster randomised controlled trial. *Trials* **19**, 302 (2018).
45. Lim, J. T. *et al.* Efficacy of Wolbachia-mediated sterility to reduce the incidence of dengue: a synthetic control study in Singapore. *Lancet Microbe* S2666-5247(23)00397-X (2024) doi:10.1016/S2666-5247(23)00397-X.
46. Lim, J. T. *et al.* Efficacy of Wolbachia-mediated sterility for control of dengue: emulation of a cluster randomized target trial. 2023.11.29.23299172 Preprint at https://doi.org/10.1101/2023.11.29.23299172 (2023).
47. Bansal, S. *et al.* Efficacy of Wolbachia-mediated sterility to suppress adult Aedes aegypti populations. 2023.11.24.23298996 Preprint at https://doi.org/10.1101/2023.11.24.23298996 (2023).
48. De Serres, G., Skowronski, D. M., Wu, X. W. & Ambrose, C. S. The test-negative design: validity, accuracy and precision of vaccine efficacy estimates compared to the gold standard of randomised placebo-controlled clinical trials. *Euro Surveill* **18**, 20585 (2013).
49. Vandenbroucke, J. P. & Pearce, N. Case-control studies: basic concepts. *Int J Epidemiol* **41**, 1480–1489 (2012).
50. Greenland, S. & Thomas, D. C. On the need for the rare disease assumption in case-control studies. *Am J Epidemiol* **116**, 547–553 (1982).
51. Anders, K. L. *et al.* Cluster-Randomized Test-Negative Design Trials: A Novel and Efficient Method to Assess the Efficacy of Community-Level Dengue Interventions. *American Journal of Epidemiology* **187**, 2021–2028 (2018).
52. Jewell, N. P., Dufault, S., Cutcher, Z., Simmons, C. P. & Anders, K. L. Analysis of cluster-randomized test-negative designs: cluster-level methods. *Biostatistics* **20**, 332–346 (2019).
53. Lai, Y.-L. *et al.* Cost-Effective Real-Time Reverse Transcriptase PCR (RT-PCR) To Screen for Dengue Virus followed by Rapid Single-Tube Multiplex RT-PCR for Serotyping of the Virus. *Journal of Clinical Microbiology* **45**, 935–941 (2007).

54. Pok, K.-Y., Lai, Y.-L., Sng, J. & Ng, L.-C. Evaluation of Nonstructural 1 Antigen Assays for the Diagnosis and Surveillance of Dengue in Singapore. *Vector-Borne and Zoonotic Diseases* **10**, 1009–1016 (2010).
55. Lee, C. *et al.* Gravitraps for Management of Dengue Clusters in Singapore. *The American Journal of Tropical Medicine and Hygiene* **88**, 888–892 (2013).
56. Department of Statistics, Singapore. SingStat. 2021. Available from: https://www.singstat.gov.sg. Cited 2024 Apr 25
57. MOH | Weekly Infectious Diseases Bulletin. https://www.moh.gov.sg/resources-statistics/infectious-disease-statistics/2021/weekly-infectious-diseases-bulletin. Cited 2024 Apr 25

58. Ong, J. *et al.* Mapping dengue risk in Singapore using Random Forest. *PLOS Neglected Tropical Diseases* **12**, e0006587 (2018).
59. Phase 1 Small-Scale Field Study. https://www.nea.gov.sg/corporate-functions/resources/research/wolbachia-aedes-mosquito-suppression-strategy/phase-1-small-scale-field-study. National Environment Agency Singapore. Available from: https://www.nea.gov.sg/corporate-functions/resources/research/wolbachia-aedes-mosquito-suppression-strategy/phase-1-small-scale-field-study.
60. Indriani, C. *et al.* Reduced dengue incidence following deployments of *Wolbachia*-infected *Aedes aegypti* in Yogyakarta, Indonesia: a quasi-experimental trial using controlled interrupted time series analysis. Preprint at https://doi.org/10.12688/gatesopenres.13122.1 (2020).
61. Wing, C., Simon, K. & Bello-Gomez, R. A. Designing Difference in Difference Studies: Best Practices for Public Health Policy Research. *Annual Review of Public Health* **39**, 453–469 (2018).
62. Infectious Diseases Act 1976 - Singapore Statutes Online. https://sso.agc.gov.sg:5443/Act/IDA1976.

Summary of amendments

Updates

Update date: 14 June 2025

Section	Details
Data management and governance	Addition on details of trial steering committee members

Update date: 1 March 2024

Section	Details
Data	Supplemental environmental and anthropogenic data to control for baseline dengue risk in intervention and control arms
Statistical analysis: primary endpoints	Addition of additional steps required to correct for potential imbalance in environmental and anthropogenic characteristics between arms
Statistical analysis: secondary endpoints	Addition of additional steps required to correct for potential imbalance in environmental and anthropogenic characteristics between arms
Selection of patients	Addition of exposure criteria based on number of months a tested individual was exposed to *Wolbachia-Aedes* interventions
