# Supplementary material for: Impact of Wolbachia-mediated incompatible and sterile insect techniques on dengue incidence: a large-scale cluster randomised controlled trial
Source: eBioMedicine. 2026 Sep 10;132:106476. doi: 10.1016/j.ebiom.2026.106476 (PMC13586744; doi:10.1016/j.ebiom.2026.106476)
Supplement: Supplementary file 2 [file mmc1.docx]

**Supplementary Information:** **Impact of *Wolbachia-mediated* incompatible and sterile insect techniques on dengue incidence: a large-scale cluster randomised controlled trial**

Table of Contents

[Data	1](#_Toc211395214)

[Production	2](#_Toc211395215)

[Constrained randomiSation protocol	2](#_Toc211395216)

[Statistical Analysis	3](#_Toc211395217)

[Robustness checks (1 of 5) Examining the impact of covariates on estimated intervention efficacies	6](#_Toc211395218)

[Robustness checks (2 of 5) Goodman-Bacon decompositions	7](#_Toc211395219)

[Robustness checks (3 of 5) In-time placebos checks	8](#_Toc211395220)

[Robustness checks (4 of 5) In-space placebos	9](#_Toc211395221)

[Robustness checks (5 of 5) Parallel trends test	14](#_Toc211395222)

# **Data**

We extracted a set of spatially explicit variables to represent environmental heterogeneity across sectors.

**Vegetation data** Based on Sentinel-2 satellite data, a 10m vegetation map^1^ of multiple classified vegetation types including grass, forest and managed vegetation was utilised to signify the availability of nectar availability and breeding areas for male mosquitoes. For each sector, the percentage cover of each vegetation type was calculated as mosquitoes often show preferential areas to breed and rest. We also calculated the averaged Landsat Normalised Difference Vegetation Index (NDVI) per sector as another proxy^2^.

**Residential data**

We collated data on the locations of public housing estates named Housing Development Board (HDBs) where over 76% of Singapore’s resident population reside was obtained from OneMap^3^ to represent urban breeding habitat density and the number of available hosts. We additionally collected data on the average age of public housing estate buildings as buildings of older ages are at greater risk of having higher Gravitrap *Aedes aegypti* index^4,5^. Older buildings typically have structural deterioration which creates additional breeding habitats in cracks and fissures as well as old undesirable design features such as laundry poles holders. Water pooling is more likely to occur in such features. Data on the average public housing estate house prices from 2015–2022, a proxy for household income and socioeconomic status, was calculated based on a previously used XGBoost model^6^. Another variable, building height, that is correlated to Gravitrap *Ae. aegypti* index, was used to represent the number of available human hosts. We estimated the height as the number of floors at an average height of 3m per floor. Additional hosts from private housing including condominiums and houses were added in the calculations. Urbanicity was estimated as the percentage cover of built area inclusive of residential, commercial and industrial land use types, which has been previously associated with *Ae. aegypti* presence^7^. Lastly, data from the Public Utilities Board on the open drainage network was obtained to represent the availability of key breeding sites for mosquitoes around public housing estates. For each sector, the average distance of each public housing estate block to a drain was measured as well as the length of the network^8^.

**Meteorological data** Well-established variables known to affect mosquito survival or fecundity were collected. We calculated the daily mean, maximum, and minimum temperature, total rainfall, maximum rainfall falling within a 30-minute, 60-minute and 120-minute window, and wind speed, which were obtained from a total of 21 weather stations installed by the National Environment Agency. Rasters at a daily resolution were created through inverse distance weighting interpolation for full spatial coverage where cross validation of leave-one-out was used for the fitting of the power to minimise error. To estimate relative humidity, we utilised dewpoint and ambient ground air temperature from ERA5, published by ECMWF^9^ with standard formula, and aggregated the values to a weekly level to match dengue case data.

**Entomological data** Adult *Aedes aegypti* populations in release sectors were monitored weekly using an average of six Gravitraps^10^ per public housing apartment block. Gravitraps were placed in public spaces along corridors of public housing apartments in all intervention sites, at the ratio of 1 trap for every 20 households, i.e. two to three traps on each of three floors per apartment block: lower floor (2nd), mid floor (5th or 6th floor) and high floor (10th or 11th floor). This trap-to-household ratio and deployment was based on logistic considerations for long term monitoring, and it provided an assessment of the density as well as vertical distribution of the mosquito population. Mosquito data was collected from all Gravitraps on a bi-weekly basis, and mosquito samples were sexed and morphologically identified. Female *Aedes* mosquitoes that cannot be speciated morphologically were identified using a multiplex *Ae. aegypti*- and *Ae. albopictus*-specific real-time qPCR assay targeting the COI gene (*Aedes* forward 5’-TCC CGC CTT CRG TGC GCG G-3’, *Aedes* reverse 5’-CGC GGG ATG TAY TCA TCA ACC-3’, probe AEG Cy5 – 5’-TAG TCA GAC GTG GTG GTG ACA CAC C-3’– BHQ2, and ALBO HEX – 5’-ACG GTG GCC GGC GTG CCA GTC GT-3’ – BHQ1). The multiplex qPCR reactions were performed in 20µl total volume containing 1X SensiFAST No-Rox Probe Mastermix (Bioline, USA); 0.15µM and 0.05µM COI primers and probes, respectively; 0.4 µM and 0.15µM wsp primers and probes, respectively; and 2µl. Cycling was carried out in a Rotorgene Q (Qiagen, Germany) using the following conditions: 95°C for 5 min, 40 cycles at 95°C for 10s, and 60°C for 30s.

**Production**
Eggs were submerged in aged hatching broth to induce hatching. The broth (0.071g brewer’s yeast (MP Biomedicals, France), 0.535g nutrient broth (Liofilchem, Italy) and 1000ml reverse osmosis (RO) water) was prepared 15 hours before use. After induced hatching for 2 hours, larvae were counted using an automated larvae counter (Orinno Technology Pte Ltd, Singapore) and transferred to rearing trays that were placed in high-density rearing racks (Orinno Technology Pte Ltd, Singapore). Each rearing tray measured 103 x 63 x 3 cm and was filled with a density of four larvae per ml of RO water. Larvae were fed with a commercial arthropod larva feed (Altech Integrated, Singapore) in slurry form.

Six days post-hatching, the rearing trays were simultaneously tilted and the mixture of larvae and pupae was collected in a collection sieve. This mixture was first treated with 15% saltwater solution for 1 hour to kill larvae, followed by placing them in water to allow vertical separation of live pupae (surface) and dead larvae (bottom)^11^. Pupae were transferred to the Pupae Separation System (Orinno Technology Pte Ltd, Singapore) where male pupae were separated from female pupae. Male pupae were held for 30 minutes prior to irradiation.

Irradiation was performed using the RS2400V irradiator (Rad Source Technologies Inc., USA). Male pupae were irradiated in plastic containers measuring 6.5 cm in diameter and 7.0 cm in height. Containers were placed at the center of the irradiation chamber of the X-ray irradiator, and the effective dose was 40 Gy. Pupae were allowed to eclose inside release containers and a 20% sugar solution was provided ad libitum prior to releases.

# **Constrained randomisation protocol**

The randomisation protocol was described previously^12^**.** Randomisation was conducted in February 2022 in the presence of the National Environment Agency’s Dengue Expert Advisory Panel and representatives from the Ministry of Sustainability and the Environment. Eight out of 15 clusters were randomly selected to receive male *Wolbachia-Aedes* deployments (intervention clusters) and the rest designated as control clusters which did not receive the intervention.

Due to the small number of clusters available for randomisation, selection of clusters relied on a constrained randomisation strategy to prevent chance imbalances in baseline characteristics between intervention and non-intervention clusters. The proportion of positive to negative dengue samples was used as the constraining variable. A large number of potential random cluster allocations in 8:7 intervention/non-intervention ratio were generated (n = 10,000). For each allocation, the value of the constraining variable was calculated in each study arm using the aggregate arm-level value. Each potential random allocation was evaluated against the pre-defined balancing criteria (i.e., no statistically significant difference in the proportion of positive to negative dengue samples between the two arms) and removed as a potential random allocation if they were not met. All potential allocations that satisfy the balancing criteria were kept (n = 3,151, exceeding the threshold of 100–150 allocations recommended in the literature), and a single allocation was randomly selected from within the restricted list of balanced allocations. Finally, a single random draw was used to determine which of the two study arms was to receive male *Wolbachia-Aedes* releases. Randomisation was followed by extensive community engagement to seek the support of local politicians and grassroot leaders of each of the eight intervention clusters for implementing the intervention. This was followed by a media announcement to inform the public of the multi-site field study on 15 June 2022.

# **Statistical analysis**

The treatment was assigned in staggered adoption setting and the treatment effect was assumed to be invariant of calendar time (*t*). Cohorts of treatment time (*e*), consisting of sets of study areas (*i*) with the same treatment start time ($E_{i}$), were expected to share the same treatment effect after the same length of post-intervention period, referred to as the relative period (*l*). We applied a robust difference-in-difference (DiD) framework introduced by Sun and Abraham to estimate treatment effect on a relative-period basis^13^.

**SI Table 1. Intervention adoption dates among intervention clusters**

| **Intervention start time** | **Clusters** |
| --- | --- |
| 2022 E-week 30 (August) | Circuit Rd, Yew Tee |
| 2022 E-week 35 (September) | Bedok North Rd, Punggol |
| 2022 E-week 37 (October) | Bedok Reservoir Rd, Sengkang, Woodlands, Hougang Ave |

In this study, we estimated the temporal heterogeneous treatment effects of *Wolbachia* releases on dengue incidence rate $\frac{Y_{i,t}}{pop_{i}}$, where $Y_{i,t}$ denoted the dengue incidence at time *t* in study area *i* and $pop_{i}$ denoted the population at risk in study area *i*. The at-risk population in each study area was included as an offset to mitigate potential estimation bias due to unequal at-risk population sizes across study areas. Dengue incidence $Y_{i,t}$ is assumed to follow a Poisson distribution across study areas *i* for each monthly interval *t*. Poisson generalised linear model (GLM) with a log link and an offset on population was specified to model the incidence rate as:

$$Y_{i,t}\sim Poisson\left( \lambda\right), \log\left( \lambda\right)\sim\log\left( pop_{i} \right)+\beta\boldsymbol{X}$$

where *X* represented the design matrix and $\beta$ the corresponding coefficient vector. We considered two models, one included temporal environmental covariates $\boldsymbol{X}_{env}$ of the study area and another which excluded them. Cohort average treatment effects in treated study areas (CATT) $\delta_{e,l}$ were estimated by the coefficients of cohort-period indicator terms $1\left\{ E_{i}=e \right\}\cdot D_{i,t}^{l}$, where:

$$1\left\{ E_{i}=e \right\}\left\{ \begin{aligned} 1, if E_{i}=e \\ 0, otherwise \end{aligned} \right. and D_{i,t}^{l}=\left\{ \begin{aligned} 1, if l=t-E_{i} \\ 0, otherwise \end{aligned} \right.$$

A Poisson GLM without additional covariates was then expressed as:

$$\log\left( E\left( Y_{i,t} \right) \right)= \alpha_{i}+\theta_{t}+ \sum_{e\notin C} \sum_{l\neq-1} \delta_{e,l}\left( 1\left\{ E_{i}=e \right\}\cdot D_{i,t}^{l} \right)+\log\left( pop_{i} \right)$$

where $\alpha_{i}$ denoted unit fixed effects, $\theta_{t}$ denoted time fixed effects and $\delta_{e,l}$ was estimated using surveillance data that satisfied the assumption that study areas *i* belonged to treatment time cohort *e* and (2) the temporal records at time *t* corresponded to post-intervention period *l*. The Poisson GLM including temporal-environmental variables was expressed as:

$$\log\left( E\left( Y_{i,t} \right) \right)= \alpha_{i}+\theta_{t}+ \sum_{e\notin C} \sum_{l\neq-1} \delta_{e,l}\left( 1\left\{ E_{i}=e \right\}\cdot D_{i,t}^{l} \right)+\gamma\boldsymbol{X}_{env}+\log\left( pop_{i} \right)$$

where the additional coefficient vector $\gamma$ captured climate effects through temporal-environmental covariates. Cohort-averaged treatment effect for the untreated cohort ($E_{i}=\infty$) or the reference relative period (e.g. one time unit before treatment, $l= -1$) were not estimated due to perfect multicollinearity.

To obtain the interaction-weighted treatment effect $\nu_{g}$ on dengue incidence rates across a set of relative periods $g=\left\{ l_{1},l_{2},\ldots\right\}$, a weighted average of CATT was calculated. The weight of each CATT $\delta_{e,l}$ was based on sample share of its corresponding cohort *e* at relative period *l* among all cohorts with a treatment period of at least length *l*. The weighted estimates $\hat{\delta}_{l}$ was calculated as:

$$\hat{\delta}_{l}= \sum_{e} \hat{\delta}_{e,l}\Pr\left( E_{i}=e \right|E_{i}\in[-l,T-l])$$

which can be interpreted as average treatment effect on treated (ATT) after *l* treatment period*.* The interaction-weighted treatment effect $\nu_{g}$ for a relative period set $g$ was calculated as

$$\nu_{g}=\frac{1}{\left| g \right|} \sum_{l_{g}\in g} \hat{\delta}_{l_{g}} =\frac{1}{\left| g \right|} \sum_{l_{g}\in g} \sum_{e} \hat{\delta}_{e,l}\cdot\hat{\Pr}\left\{ E_{i}=e | E_{i}\in\left[ -l,T-l \right] \right\}$$

where $l_{g}$ represented an arbitrary relative period *l* in set *g.* The null hypothesis of $\nu_{g}$ was that the treatment effect was not statistically significant during the specific relative period set *g*. Sun and Abraham showed that $\nu_{g}$ is asymptotically normal with a derived asymptotic variance ^13^. To estimate intervention effectiveness for every 3-month interval since intervention onset, we defined the relative period treatment estimator $\hat{\delta}_{g}$ based on weighted estimates $\hat{\delta}_{l}$. As *g* represented a set of 3 monthly relative periods in the study, $\hat{\delta}_{g}$ would be instituted by one of the three corresponding weighted estimates $\hat{\delta}_{l_{g}}$ with $l_{g}\in g$, with no weightage over time. Thus, a valid estimation of $\hat{\delta}_{g}$ was a simple additive average of monthly ATTs $\hat{\delta}_{l_{g}}$. The Poisson GLM specification for dengue incidence in a specific relative period is:

$$\log\left( E\left( Y_{i,t_{g}} \right) \right)=\log\left( \lambda\right)= \alpha_{i}^{g}+ \theta_{t_{g}}^{g}+\hat{\delta}_{g}\cdot D\left( t-E_{i}\in g \right)+\log\left( pop \right)$$

where $\alpha_{i}^{g}$ and $\theta_{t_{g}}^{g}$ denoted unit and time fixed effects for relative period set *g.* The equation above omitted weighted estimates with $D(t-E_{i}\in g)=0$.

Intervention effectiveness (IE) was defined as the proportion reduction in dengue incidence. Therefore, the IE of a treated study area *i* and treatment time $t=E_{i}+l_{g}$ was derived as

$$IE=-\frac{E\left( Y_{g} \left( I_{d}=1 \right) \right)-E\left( Y_{g}\left( I_{d}=0 \right) \right)}{E\left( Y_{g}\left( I_{d}=0 \right) \right)}=- \frac{e^{\alpha_{i}^{g}+\theta_{t_{g}}^{g}+\hat{\delta}_{g}+\log\left( pop \right)}-e^{\alpha_{i}^{g}+\theta_{t_{g}}^{g}+\log\left( pop \right)}}{e^{\alpha_{i}^{g}+\theta_{t_{g}}^{g}+\log\left( pop \right)}}= 1- e^{\hat{\delta}_{g}}$$

where $Y_{g}$ represented outcomes over the relative period set *g*, with $I_{d}=1$ indicating intervention outcomes and $I_{d}=0$ control outcomes. $\alpha_{i}^{g}$ and $\theta_{t_{g}}^{g}$were unit and time fixed effects respectively for the GLM model with a log link over relative period set *g*. As discussed previously, the additive treatment effect could be estimated by the average of treatment effects during the relative period, therefore:

$$IE=1- e^{\hat{\delta}_{g}}=1-e^{\frac{1}{\left| g \right|}\sum_{l_{g}\in g} {\hat{\delta}_{l}}_{g}}=1-e^{\nu_{g}}$$

Average raw reduction on dengue incidence $R_{g}$ in relative period set $g$ was defined as the mean difference between observed intervention outcomes and counterfactual outcomes across intervened study areas:

$$R_{g}=E\left( Y_{g}\left( I_{d}=1 \right)-Y_{g}\left( I_{d}=0 \right) \right)=E\left( \sum_{l\in g} \left( Y_{i,t}(I_{d}=1)-Y_{i,t}\left( I_{d}=0 \right) \right) \right)=\sum_{l\in g} \left( Y_{i,t}\left( I_{d}=1 \right)-\hat{Y}_{i,t}\left( I_{d}=0 \right) \right)$$

where the counterfactual outcomes $\hat{Y}_{i,t}\left( I_{d}=0 \right)$ were estimated using the previous specific modelled GLM. Paired sample t-tests were conducted to construct 95% CI of $R_{g}$.

**SI Table 2. Sex and age distribution of population demographics and reported cases**

|  | **Control** | | | | **Intervention** | | | | **SMD^*^** |
| --- | --- | --- | --- | --- | --- | --- | --- | --- | --- |
|  | **Population** | **Pre-intervention** | **During intervention** | **SMD^*^ (pre- vs during)** | **Population** | **Pre-intervention** | **During intervention** | **SMD (pre- vs during)** | **Population (control vs intervention)** |
| Female (%) | 51.6 | 45.7 | 47.5 | 0.04 | 50.8 | 45.2 | 41.3 | 0.08 | 0.015 |
| Age 0-20 (%) | 15.0 | 14.3 | 13.6 | 0.01 | 15.7 | 14.9 | 12.1 | 0.07 | 0.02 |
| Age 21-64 (%) | 59.2 | 71.5 | 67.4 | 0.11 | 61.8 | 73.9 | 73.1 | 0.03 | 0.05 |
| Age 65 + (%) | 25.8 | 14.2 | 19.0 | 0.13 | 22.5 | 11.3 | 14.9 | 0.11 | 0.08 |

^*^Standardized mean differences calculated as $\left| \mathrm{SMD} \right|=\left| \frac{p_{1}-p_{0}}{\sqrt{\frac{p_{1}\left( 1-p_{1} \right)+p_{0}\left( 1-p_{0} \right)}{2}}} \right|$

**SI Table 3. Population size and reported cases by clusters**

| **Control clusters** | | | | **Intervention clusters** | | | |
| --- | --- | --- | --- | --- | --- | --- | --- |
|  | **Population** | **Pre-intervention (cases)** | **During intervention (cases)** |  | **Population** | **Pre-intervention (cases)** | **During intervention (cases)** |
| Ang Mo Kio | 83136 | 1623 | 313 | Bedok North Rd | 70708 | 889 | 155 |
| Bukit Panjang | 38872 | 943 | 585 | Bedok Reservoir Rd | 44232 | 1265 | 74 |
| Pasir Ris | 33328 | 864 | 221 | Circuit Rd | 38704 | 991 | 96 |
| Pioneer | 40140 | 700 | 385 | Hougang Ave | 37292 | 877 | 223 |
| Serangoon Central | 38064 | 703 | 283 | Punggol | 48480 | 360 | 66 |
| Tao Payoh | 67736 | 1027 | 841 | Sengkang | 50164 | 778 | 81 |
| Yuhua | 29916 | 405 | 226 | Woodlands | 64168 | 1699 | 269 |
|  |  |  |  | Yew Tee | 39488 | 457 | 73 |
| Total | 331192 | 6265 | 2854 | Total | 393236 | 7316 | 1037 |

**SI Figure 1. Age distribution of reported dengue cases and demographic composition, visualized by cluster, arm and pre- and post- intervention periods.** Age groups were stratified by children and adolescents (0-19), adults (20-64) and elderly (65+).

**SI Table 4. Estimated intervention efficacies for total, epidemiologically-linked, or sporadic dengue incidence rates using the interaction-weighted differences-in-differences estimator with covariates, with or without age adjustment in the outcome regression. Bolded** figure represents 95% CIs which do not cross 0.

|  | **Main results** | | | **Exploratory analysis: age adjustment** | | |
| --- | --- | --- | --- | --- | --- | --- |
|  | **All** | **Linked** | **Sporadic** | **All** | **Linked** | **Sporadic** |
| 0-2 months | -56.2 (-130.2, -5.9) | -52.2 (-154.9, 9.1) | -61.3 (-169.0, 3.3) | -53.5 (-125.1, -4.6) | -48.5 (-146.0, 10.3) | -58.4 (-158.4, 2.9) |
| 3-5 months | 7.3 (-94.9, 55.9) | 41.4 (-66.1, 79.3) | -58.4 (-345.6, 43.7) | 10.4 (-82.8, 56.1) | 45.6 (-47.1, 79.9) | -59.3 (-322.0, 39.9) |
| 6-8 months | **63.9 (4.5, 86.4)** | **88.7 (63.9, 96.5)** | -45.6 (-252.8, 39.9) | **63.8 (9.3, 85.6)** | **88.5 (65.7, 96.1)** | -47.2 (-240.0, 36.3) |
| 9-11 months | 79.4 (-17.6, 96.4) | **92.6 (49.6, 98.9)** | -37.4 (-163.9, 28.4) | 79.6 (-4.7, 96.0) | **92.7 (55.6, 98.8)** | -33.7 (-145.9, 27.4) |
| 12-14 months | 37.3 (-13.9, 65.5) | **68.6 (22.6, 87.3)** | -21.1 (-138.7, 38.5) | 37.0 (-7.8, 63.2) | **68.6 (27.1, 86.5)** | -22.5 (-137.9, 36.9) |
| 15-17 months | 27.6 (-35.4, 61.3) | **60.9 (21.3, 80.6)** | -72.1 (-275.3, 21.1) | 30.3 (-26.9, 61.8) | **62.7 (28.2, 80.6)** | -69.0 (-244.2, 17.1) |
| 18-20 months | **83.8 (50.4, 94.7)** | **99.8 (99.2, 99.9)** | -16.8 (-178.1, 50.9) | **84.9 (56.8, 94.7)** | **99.8 (99.2, 99.9)** | -14.7 (-155.2, 48.5) |
| 21-23 months | **65.9 (8.6, 87.3)** | **91.6 (70.8, 97.6)** | -30.8 (-171.9, 37.1) | **66.3 (14.0, 86.8)** | **91.6 (72.8, 97.4)** | -30.1 (-158.5, 34.6) |

**Robustness checks (1 of 5) Examining the impact of covariates on estimated intervention efficacies**

We examined the impact of including or excluding covariates on the estimated intervention efficacies on dengue incidence rates and epidemiologically-linked/sporadic dengue incidence rates. The estimated treatment effects were similar when excluding or including covariates from our analytical framework (**SI Table 5**).

**SI Table 5. Estimated intervention efficacies for total, epidemiologically-linked or sporadic dengue incidence rates using the interaction-weighted differences-in-differences estimator, with or without covariates.** **Bolded** figures represent 95% CIs which do not cross 0

|  | **No covariates**  **(all)** | **Covariates**  **(all)** | **No covariates**  **(linked)** | **Covariates**  **(linked)** | **No covariates**  **(sporadic)** | **Covariates**  **(sporadic)** |
| --- | --- | --- | --- | --- | --- | --- |
| 0-2 months | -41.0 (-90.2, -4.6) | -56.2 (-130.2, -5.9) | -32.3 (-95.5, 10.5) | -52.2 (-154.9, 9.1) | -58.2 (-165.4, 5.8) | -61.3 (-169.0, 3.3) |
| 3-5 months | 21.7 (-68.0, 63.5) | 7.3 (-94.9, 55.9) | 52.1 (-36.8, 83.2) | 41.4 (-66.1, 79.3) | -49.6 (-318.6, 46.5) | -58.4 (-345.6, 43.7) |
| 6-8 months | 61.2 (-7.6, 86.0) | 63.9 (4.5, 86.4) | 87.7 (58.5, 96.4) | 88.7 (63.9, 96.5) | -48.2 (-264.1, 39.7) | -45.6 (-252.8, 39.9) |
| 9-11 months | 77.2 (-47.9, 96.5) | 79.4 (-17.6, 96.4) | 91.9 (34.6, 99.0) | 92.6 (49.6, 98.9) | -44.3 (-174.9, 24.3) | -37.4 (-163.9, 28.4) |
| 12-14 months | 34.9 (-13.5, 62.7) | 37.3 (-13.9, 65.5) | 67.7 (24.0, 86.3) | 68.6 (22.6, 87.3) | -24.0 (-142.3, 36.6) | -21.1 (-138.7, 38.5) |
| 15-17 months | 37.6 (-14.5, 66.1) | 27.6 (-35.4, 61.3) | 67.1 (33.7, 83.7) | 60.9 (21.3, 80.6) | -64.4 (-243.3, 21.3) | -72.1 (-275.3, 21.1) |
| 18-20 months | 81.4 (41.7, 94.1) | 83.8 (50.4, 94.7) | 99.7 (99.0, 99.9) | 99.8 (99.2, 99.9) | -22.2 (-183.9, 47.4) | -16.8 (-178.1, 50.9) |
| 21-23 months | 62.1 (-14.7, 87.5) | 65.9 (8.6, 87.3) | 90.5 (59.5, 97.8) | 91.6 (70.8, 97.6) | -37.8 (-170.7, 29.8) | -30.8 (-171.9, 37.1) |

**Robustness checks (2 of 5) Goodman-Bacon decompositions**

We employed the Goodman-Bacon decomposition to examine if the staggered adoption setting led to problematic comparisons in our differences-in-differences identification setup^14^. We used the Goodman-Bacon decompositions to ascertain number of bad comparisons under the standard two-way fixed effects differences-in-differences framework. While a large number of comparisons were not problematic (i.e, treated vs. untreated), a small proportion comprised problematic comparisons between using earlier treated units as the control for later treated units, which biased estimated treatment effects downwards under the standard differences-in-differences setup (**SI Table 6**).

**SI Table 6. Goodman-Bacon decompositions using total, epidemiologically-linked or sporadic dengue incidence rates.** Comparisons pertain to whether intervention sites in earlier/later periods were compared to control sites in later/earlier periods. Comparisons between earlier treated units and using later treated units as controls were deemed problematic if the assumption of static intervention effects over time were not met.

| **Comparison** | **All cases** | | **Linked cases** | | **Sporadic cases** | |
| --- | --- | --- | --- | --- | --- | --- |
|  | **Weight** | **Average treatment effect estimate** | **Weight** | **Average treatment effect estimate** | **Weight** | **Average treatment effect estimate** |
| Earlier Control vs Later Treated | 0.02034 | -0.2454 | 0.02034 | -0.35637 | 0.02034 | 0.4924 |
| Later Control vs Earlier Treated | 0.00456 | 0.05593 | 0.00456 | 0.44599 | 0.00456 | 0.31011 |
| Treated vs Untreated | 0.9751 | -1.03688 | 0.9751 | -1.4464 | 0.9751 | -0.04688 |

**Robustness checks (3 of 5) In-time placebos checks**

We examined the estimated intervention efficacies in the time before interventions began (12 months prior and 24 months before) using models with and without covariates, to examine if our analytical framework results in spurious detection of intervention efficacies in the period prior to the intervention. There were no estimated intervention efficacies for total and epidemiologically-linked dengue incidence rates which had 95% CIs cross 0 in placebo-intervention periods starting from 12 to 24 months prior to the start of the actual intervention in intervention sites (**SI Table 7**). The period up till the start of the intervention was used to estimate placebo-intervention effects.

**SI Table 7. In-time placebo checks in the period starting from 12 or 24 months prior to the start of the actual intervention in intervention sites**. The same interaction-weighted differences-in-differences framework was used to estimate intervention efficacies in the placebo-intervention period, with and without covariates, for total, epidemiologically-linked and sporadic dengue incidence rates. **Bolded** numbers represent protective effects with 95% CIs which do not cross 0.

|  | **With covariates** | | | | | | **No covariates** | | | | | | |
| --- | --- | --- | --- | --- | --- | --- | --- | --- | --- | --- | --- | --- | --- |
|  | **All** | **All** | **Linked** | **Linked** | **Sporadic** | **Sporadic** | **All** | **All** | **Linked** | **Linked** | **Sporadic** | **Sporadic** |  |
| **Pseudo** | **12 months** | **24 months** | **12 months** | **24 months** | **12 months** | **24 months** | **12 months** | **24 months** | **12 months** | **24 months** | **12 months** | **24 months** |  |
| 0-2 months | 0.4 (-101.4, 50.7) | -0.8 (-81.6, 44.0) | -62.1 (-470.4, 54.0) | 7.2 (-91.5, 55.0) | 31.6 (-26.2, 62.9) | **-154.4 (-455.1, -16.6)** | -1.8 (-99.5, 48.1) | 13.9 (-31.7, 43.7) | -68.7 (-467.0, 49.8) | 23.2 (-33.8, 55.9) | 31.3 (-28.1, 63.2) | **-144.9 (-414.5, -16.5)** |  |
| 3-5 months | 14.9 (-103.8, 64.5) | 6.6 (-86.7, 53.2) | -6.9 (-406.0, 77.4) | -33.1 (-291.9, 54.8) | **97.5 (94.6, 98.9)** | -30.4 (-229.1, 48.3) | 19.2 (-100.3, 67.4) | 20.3 (-56.7, 59.5) | 0.4 (-384.1, 79.5) | -5.4 (-209.5, 64.1) | **97.6 (94.6, 98.9)** | -26.7 (-204.1, 47.2) |  |
| 6-8 months | 30.8 (-112.6, 77.5) | 9.3 (-133.8, 64.9) | 28.7 (-280.2, 86.6) | -6.8 (-357.6, 75.1) | 29.2 (-67.4, 70.1) | -92.2 (-311.5, 10.2) | 28.4 (-132.2, 77.9) | 6.9 (-131.9, 62.6) | 26.5 (-313.3, 86.9) | -9.4 (-364.5, 74.2) | 26.8 (-73.4, 69.1) | -96.5 (-313.4, 6.6) |  |
| 9-11 months | 61.6 (-32.4, 88.9) | 3.7 (-91.4, 51.5) | 59.8 (-144.6, 93.4) | 14.5 (-169.7, 72.9) | 35.9 (-55.7, 73.6) | **-129.0 (-401.2, -4.6)** | 61.4 (-34.5, 88.9) | 9.7 (-78.5, 54.3) | 59.8 (-141.0, 93.3) | 22.4 (-116.8, 72.2) | 34.7 (-62.3, 73.8) | **-127.8 (-372.0, -9.9)** |  |
| 12-14 months |  | -24.2 (-212.3, 50.6) |  | -71.6 (-637.8, 60.1) |  | -114.9 (-433.2, 13.4) |  | -22.8 (-164.6, 43.0) |  | -71.0 (-549.6, 55.0) |  | -114.8 (-404.4, 8.5) |  |
| 15-17 months |  | -11.0 (-61.8, 23.9) |  | -18.7 (-129.7, 38.6) |  | **92.0 (84.9, 95.8)** |  | -1.7 (-39.6, 25.9) |  | -5.7 (-90.8, 41.4) |  | **92.2 (85.5, 95.8)** |  |
| 18-20 months |  | 9.4 (-90.2, 56.9) |  | 20.6 (-79.4, 64.8) |  | **-129.5 (-308.6, -28.9)** |  | 9.7 (-99.7, 59.2) |  | 21.9 (-84.7, 67.0) |  | **-135.8 (-322.7, -31.6)** |  |
| 21-23 months |  | 49.8 (-36.4, 81.5) |  | 55.1 (-19.2, 83.1) |  | **-107.7 (-247.4, -24.2)** |  | 51.3 (-26.6, 81.3) |  | 57.3 (-18.8, 84.6) |  | **-110.4 (-247.0, -27.5)** |  |

**Robustness checks (4 of 5) In-space placebos**

We conducted the in-space placebo checks by randomly reassigning sites in the control arm to be intervention sites and re-running our analytical framework to estimate average treatment effects and intervention efficacies in the actual intervention period among the control sites. We reassigned the intervention allocation to control units while removing the actual intervention sites from the analysis. We iteratively took 1, 2 or 3 control sites to be allocated to a placebo intervention arm and used the remaining 6, 5 or 4 control sites as the remaining control arm. We repeated all analytical procedures to obtain the average treatment effect and intervention efficacy, for each time-period since intervention for total or epidemiologically-linked dengue incidence rates under the model with or without covariates. Estimated placebo intervention efficacies were on average 0, indicating that our analytical framework does not lead to spurious detection of intervention effects in the actual intervention period (**SI Figure 2–5**).

**SI Figure 2. Visualisation of intervention efficacies of release of *Wolbachia-Aedes* on total dengue incidence by time since treatment using the model without covariates, by allocating placebo interventions randomly to control sites and taking the remaining controls as the comparator group.** The same analytical framework was used as that of the main analysis. The vertical line is the median IE.

**SI Figure 3. Visualisation of intervention efficacies of release of *Wolbachia-Aedes* on epidemiologically-linked dengue incidence by time since treatment using the model without covariates, by allocating placebo interventions randomly to control sites and taking the remaining controls as the comparator group.** The same analytical framework was used as that of the main analysis. The vertical line is the median IE.

**SI Figure 4. Visualisation of intervention efficacies of release of *Wolbachia-Aedes* on total dengue incidence by time since treatment using the model with covariates, by allocating placebo interventions randomly to control sites and taking the remaining controls as the comparator group.** The same analytical framework was used as that of the main analysis. The vertical line is the median IE.

**SI Figure 5. Visualisation of intervention efficacies of release of *Wolbachia-Aedes* on epidemiologically-linked dengue incidence by time since treatment using the model with covariates, by allocating placebo interventions randomly to control sites and taking the remaining controls as the comparator group.** The same analytical framework was used as that of the main analysis.

**Robustness checks (5 of 5) Parallel trends test**

We reported pre-intervention intervention efficacy on total, epidemiologically-linked, and sporadic incidence rates. The 95% CI of estimated efficacies all crossed 0, indicating no significant difference in pre-treatment trends between arms.

**SI Figure 6. Visualisation of pre-intervention intervention efficacies of release of *Wolbachia-Aedes* on total dengue incidence.** Estimated intervention efficacy is shown in 3-month bins.

**SI Figure 7. Visualisation of pre-intervention intervention efficacies of release of *Wolbachia-Aedes* on epidemiologically-linked dengue incidence.** Estimated intervention efficacy is shown in 3-month bins.

**SI Figure 8. Visualisation of pre-intervention intervention efficacies of release of *Wolbachia-Aedes* on sporadic dengue incidence.** Estimated intervention efficacy is shown in 3-month bins.

**References**

1	Gaw LY-F, Yee ATK, Richards DR. A High-Resolution Map of Singapore’s Terrestrial Ecosystems. *Data* 2019; **4**: 116.

2	Landsat Normalized Difference Vegetation Index | U.S. Geological Survey. https://www.usgs.gov/landsat-missions/landsat-normalized-difference-vegetation-index (accessed Sept 26, 2024).

3	OneMap. OneMap. http://www.onemap.gov.sg (accessed Nov 9, 2023).

4	Sun H, Dickens BL, Richards D, *et al.* Spatio-temporal analysis of the main dengue vector populations in Singapore. *Parasites & Vectors* 2021; **14**: 41.

5	Tewari P, Guo P, Dickens B, Ma P, Bansal S, Lim JT. Associations between Dengue Incidence, Ecological Factors, and Anthropogenic Factors in Singapore. *Viruses* 2023; **15**: 1917.

6	Park SH, Nicolaou M, Dickens BSL, Yang Q, Tan KW, van Dam RM. Ethnicity, Neighborhood and Individual Socioeconomic Status, and Obesity: The Singapore Multiethnic Cohort. *Obesity (Silver Spring)* 2020; **28**: 2405–13.

7	Kolimenakis A, Heinz S, Wilson ML, *et al.* The role of urbanisation in the spread of Aedes mosquitoes and the diseases they transmit—A systematic review. *PLOS Neglected Tropical Diseases* 2021; **15**: e0009631.

8	Fernandez SA, Sun H, Dickens BL, Ng LC, Cook AR, Lim JT. Features of the urban environment associated with Aedes aegypti abundance in high-rise public apartments in Singapore: An environmental case-control study. *PLOS Neglected Tropical Diseases* 2023; **17**: e0011075.

9	ECMWF. ECMWF. 2023; published online Oct 3. https://www.ecmwf.int/ (accessed Nov 9, 2023).

10	Lee C, Vythilingam I, Chong C-S, *et al.* Gravitraps for Management of Dengue Clusters in Singapore. *Am J Trop Med Hyg* 2013; **88**: 888–92.

11	Chng KK, Liew JWK, Ang SC, *et al.* Enhanced mosquito sex separation using NaCl for optimised sterile insect technique and incompatible insect technique programmes. *Scientific Reports*.

12	Ong J, Ho SH, Soh SXH, *et al.* Assessing the efficacy of male Wolbachia-infected mosquito deployments to reduce dengue incidence in Singapore: study protocol for a cluster-randomized controlled trial. *Trials* 2022; **23**: 1023.

13	Sun L, Abraham S. Estimating dynamic treatment effects in event studies with heterogeneous treatment effects. *Journal of Econometrics* 2021; **225**: 175–99.

14	Goodman-Bacon A. Difference-in-differences with variation in treatment timing. *Journal of Econometrics* 2021; **225**: 254–77.
